# Medicinally Tuned Pyrimidine–Oxadiazole Hybrids: Synthetic Development, Enzyme-Targeted Evaluation, In Vivo Toxicological Assessment and Computational Investigations Against Diabetes Mellitus

**DOI:** 10.3390/ph19071085

**Published:** 2026-07-15

**Authors:** Shifa Felemban, M. M. Khowdiary

**Affiliations:** 1 Department of Chemistry, Faculty of Applied Science, University College-Al Leith, University of Umm Al-Qura, Makkah 21955, Saudi Arabia; 2 Department of Chemistry, Faculty of Science, Umm Al-Qura University, Makkah 21955, Saudi Arabia

**Keywords:** pyrimidine–oxadiazole derivatives, α-amylase inhibition, α-glucosidase inhibition, antidiabetic agents, structure–activity relationship, molecular docking, in vivo toxicity evaluation

## Abstract

**Backgroud:** The growing prevalence of diabetes mellitus necessitates the development of safe and effective inhibitors of carbohydrate-metabolizing enzymes, particularly α-amylase and α-glucosidase. **Methods:** In this study, a series of pyrimidine–oxadiazole derivatives (1–10) was synthesized and structurally characterized using elemental analysis, HREI-MS, and ^1^H/^13^C NMR spectroscopy. The compounds were evaluated for in vitro inhibitory activity against both enzymes, with acarbose as the reference drug. **Results:** IC_50_ values ranged from 6.70 ± 0.20 to 21.10 ± 0.10 μM for α-amylase and 7.10 ± 0.20 to 21.80 ± 0.40 μM for α-glucosidase. Compounds **2**, **3**, and **6** displayed superior dual inhibitory activity compared to acarbose (IC_50_ = 10.10 ± 0.20 and 10.50 ± 0.10 μM, respectively). Structure–activity relationship analysis revealed that electronic effects of aromatic substitutions significantly influenced enzyme inhibition. Molecular docking supported the experimental findings by demonstrating stable binding interactions within the enzyme active sites. Preliminary safety profiling in male Wistar rats showed no observable behavioral changes, hematological abnormalities, or hepatic and renal dysfunction following repeated administration of the lead compound. **Conclusions:** These results highlight pyrimidine–oxadiazole derivatives as promising and well-tolerated dual enzyme inhibitors for further antidiabetic drug development.

## 1. Introduction

Diabetes, a chronic disease, is mainly caused by improper insulin secretion by the pancreas. Various symptoms of diabetes include dysfunction, damage to internal organ as well as long term complications [1]. Insulin deficiency is caused by improper metabolism of lipids, proteins and carbohydrates which result in health disorders and in some cases may lead to death. According to the latest report from the International Diabetes Federation (IDF) Diabetes Atlas (11th edition, 2025), approximately 589 million adults aged 20–79 years are currently living with diabetes, representing nearly 11.1% of the global population (approximately one in nine adults). This number is projected to rise to 853 million by 2050, reflecting a substantial and ongoing increase in disease burden [2]. Patients suffering from diabetes have increased levels of glucose after eating a meal. This condition is known as postprandial hyperglycemia (PPHG). Diabetes mellitus also causes various other diseases including retinopathy, cardiovascular diseases, neuropathy, etc. [3]. This disease is mainly caused by α-amylase and α-glucosidase known as diabetic enzymes. α-amylase converts complex polysaccharides into maltose and α-glucosidase breaks down maltose into a simpler glucose unit. This conversion results in increased blood glucose level [4]. Different marketed drugs including acarbose, miglitol and voglibose are used as inhibitors of both diabetic enzymes but these drugs also cause various other diseases such as abdominal pain and diarrhea [5,6]. In this regard, it is necessary to design and synthesize such a drug which is more potent, cost effective and has minimal severe side effects.

Heterocyclic compounds are a class of organic compounds that contain biologically active moieties used to treat various diseases [7,8,9,10]. Among them sulfur- and nitrogen-containing moieties are biologically more active and are of great interest for drug designers and researchers [11,12]. One of the nitrogen-containing heterocyclic compounds with electron dense structure is pyrimidine which serves as a foundational building block in DNA and RNA. Naturally occurring in the human body, this molecule plays a vital role in genetic coding and biological processes [13]. Due to its ease of synthesis and adaptable structure, the pyrimidine molecular framework has become a key structure in drug development. Its versatile structure allows it to be used against various infectious diseases like malaria and viral infections to cancer, parasitic illnesses, and chronic issues such as inflammation, pain, seizures, high blood pressure, and oxidative stress. This adaptability highlights pyrimidine’s importance in creating therapies for diverse medical challenges [14,15,16,17,18,19,20,21]. Studies also suggest pyrimidine-based compounds are used for developing treatments targeting the central nervous system (CNS), regulating calcium channels, and addressing depression. Their diverse biological activity further solidifies their role in advancing therapies for neurological and psychological conditions [22,23].

In recent years, heterocyclic scaffolds such as pyrimidine and 1,3,4-oxadiazole have attracted considerable attention due to their broad pharmacological relevance and ability to interact with multiple biological targets involved in diabetes progression. Pyrimidine derivatives are well-documented as privileged structures in antidiabetic drug design, exhibiting inhibitory activity against key enzymes such as α-glucosidase, α-amylase, and DPP-4, and several pyrimidine-based molecules have advanced into clinical use, highlighting their therapeutic relevance [24]. Similarly, 1,3,4-oxadiazole derivatives have been widely explored as bioactive pharmacophores with notable enzyme inhibitory potential, including carbohydrate-hydrolyzing enzymes relevant to postprandial glucose regulation [25].

More recently, molecular hybridization strategies involving the combination of two or more bioactive pharmacophores have emerged as an effective approach to enhance binding affinity, selectivity, and pharmacokinetic properties compared to single-scaffold molecules. In particular, pyrimidine–oxadiazole hybrid systems have shown promising biological activities, including antimicrobial, anti-inflammatory, and antidiabetic potential, supported by docking and structure–activity relationship (SAR) studies [26].

**Figure 1:**
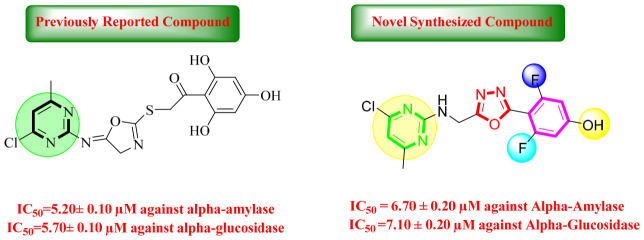
Rational design of pyrimidine–oxadiazole hybrid derivatives through pharmacophoric fusion in comparison to the previously reported compounds.

Despite these advancements, a clear research gap remains: most reported pyrimidine or oxadiazole-based inhibitors focus on single-target activity or lack integrated evaluation of dual enzyme inhibition relevant to diabetes management. Furthermore, limited studies have systematically explored the synergistic effect of pyrimidine–oxadiazole hybridization on α-amylase and α-glucosidase inhibition within a unified scaffold. Therefore, there is a strong need to design and develop novel hybrid molecules that can simultaneously target multiple enzymes involved in glucose metabolism while improving overall inhibitory efficiency. The present study focuses on the rational design of pyrimidine–oxadiazole hybrid derivatives to enhance enzyme inhibition through pharmacophoric fusion in comparison to the previously reported compounds [27] as shown in Figure 1.

In the present study, we report the design, synthesis, and biological evaluation of a novel series of pyrimidine–oxadiazole hybrid derivatives as potential dual inhibitors of carbohydrate-hydrolyzing enzymes. Although pyrimidine and 1,3,4-oxadiazole scaffolds have individually been reported as α-amylase and α-glucosidase inhibitors, the present study introduces a structurally distinct pyrimidine–oxadiazole hybrid framework featuring a rationally designed substitution pattern that has not been extensively explored in previous reports. Unlike earlier studies that primarily focused on single pharmacophores or limited substitution diversity, the current series incorporates strategically positioned electron-donating and electron-withdrawing groups to modulate electronic distribution and enhance enzyme–ligand interactions. This tailored substitution pattern is expected to influence binding affinity through improved hydrogen bonding, π–π stacking, and electrostatic interactions within the active sites of target enzymes. Furthermore, the synthesized compounds demonstrate promising dual enzyme inhibition along with a favorable preliminary safety profile, suggesting an advantage over previously reported analogs that often exhibit moderate activity or lack multitarget potential. Therefore, the novelty of this study lies not only in the hybrid scaffold design but also in the systematic exploration of substitution effects contributing to enhanced biological activity and potential therapeutic relevance. The study further integrates in silico docking analysis to rationalize binding interactions and support structure–activity relationships, aiming to identify lead candidates with enhanced antidiabetic potential.

## 2. Result and Discussion

### 2.1. Chemistry

**Scheme 1:**
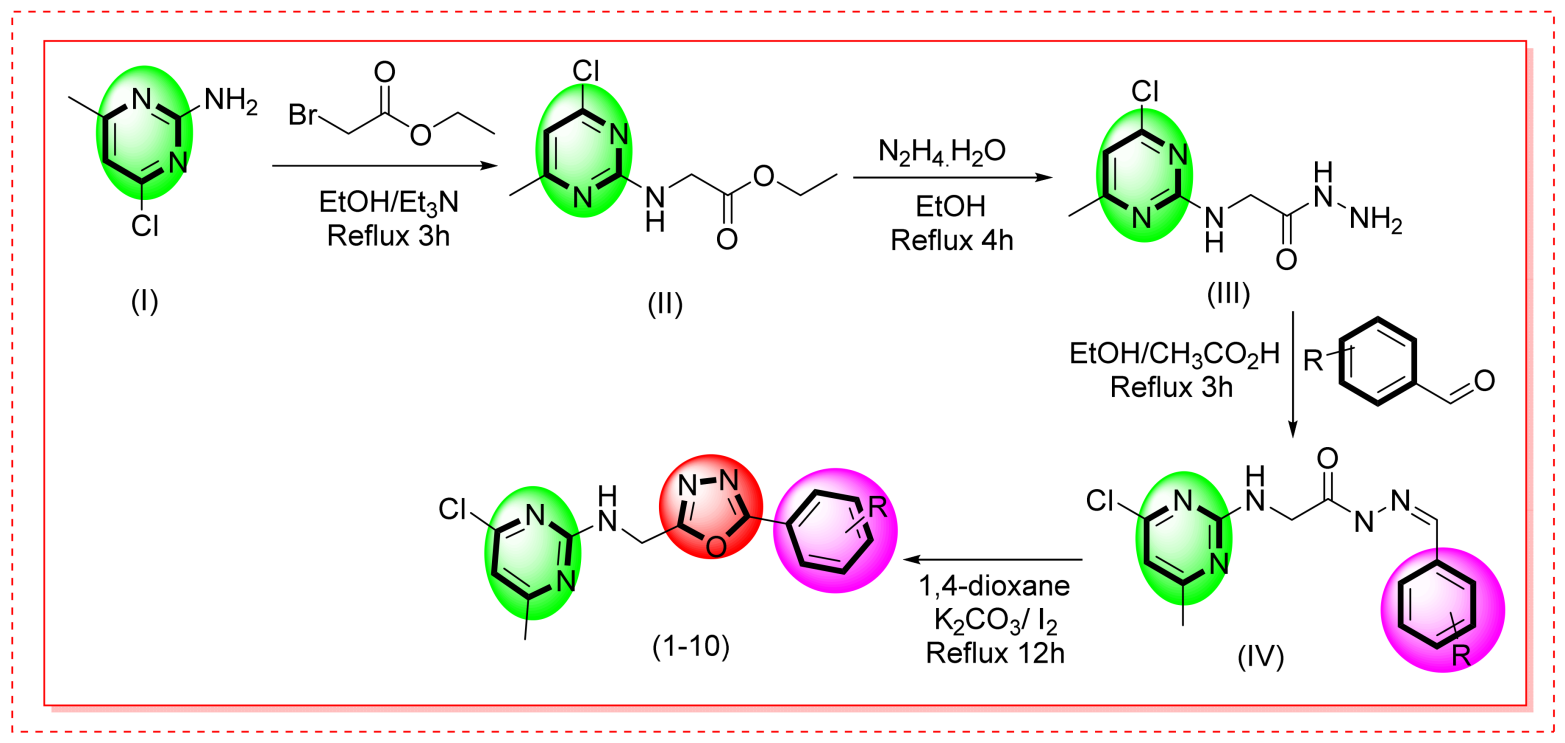
Route to synthesize pyrimidine-based oxadiazole derivatives (1–10).

The synthetic route adopted for the preparation of pyrimidine-based 1,3,4-oxadiazole derivatives (1–10) is outlined in Scheme 1. The synthesis commenced with 4-chloro-6-methylpyrimidin-2-amine (I), which was subjected to nucleophilic substitution with ethyl 2-bromoacetate in the presence of triethylamine to afford the corresponding ester intermediate (II). This transformation proceeds via substitution of the bromide moiety by the nucleophilic amino group of the pyrimidine scaffold. Subsequently, intermediate II was converted into the corresponding hydrazide (III) through reaction with hydrazine hydrate in ethanol under reflux for 4 h. The obtained intermediate III was then condensed with a series of substituted benzaldehydes under acidic conditions for 3 h in the presence of ethanol and acetic acid to yield the corresponding acylhydrazone intermediates (IV). This step allows structural diversification through variation in the substituents (R) on the aromatic ring.

The final cyclization step for the synthesis of the target pyrimidine–oxadiazole derivatives (1–10) was carried out via oxidative cyclodehydration of the corresponding acylhydrazone intermediate (IV). The intermediate was refluxed in 1,4-dioxane in the presence of iodine (I_2_, catalytic amount) and potassium carbonate (K_2_CO_3_) for 10–12 h. This method is consistent with reported procedures where iodine acts as an efficient oxidizing and cyclizing agent for the formation of 1,3,4-oxadiazoles from acylhydrazones [28]. All synthesized compounds were obtained in good to excellent yields and were characterized by ^1^H NMR, ^13^C NMR, and mass spectrometry. The physical properties and spectral data of the synthesized derivatives are summarized in Table 1.

### 2.2. Inhibitory Effects on α-Amylase and α-Glucosidase Enzymes

**Figure 2:**
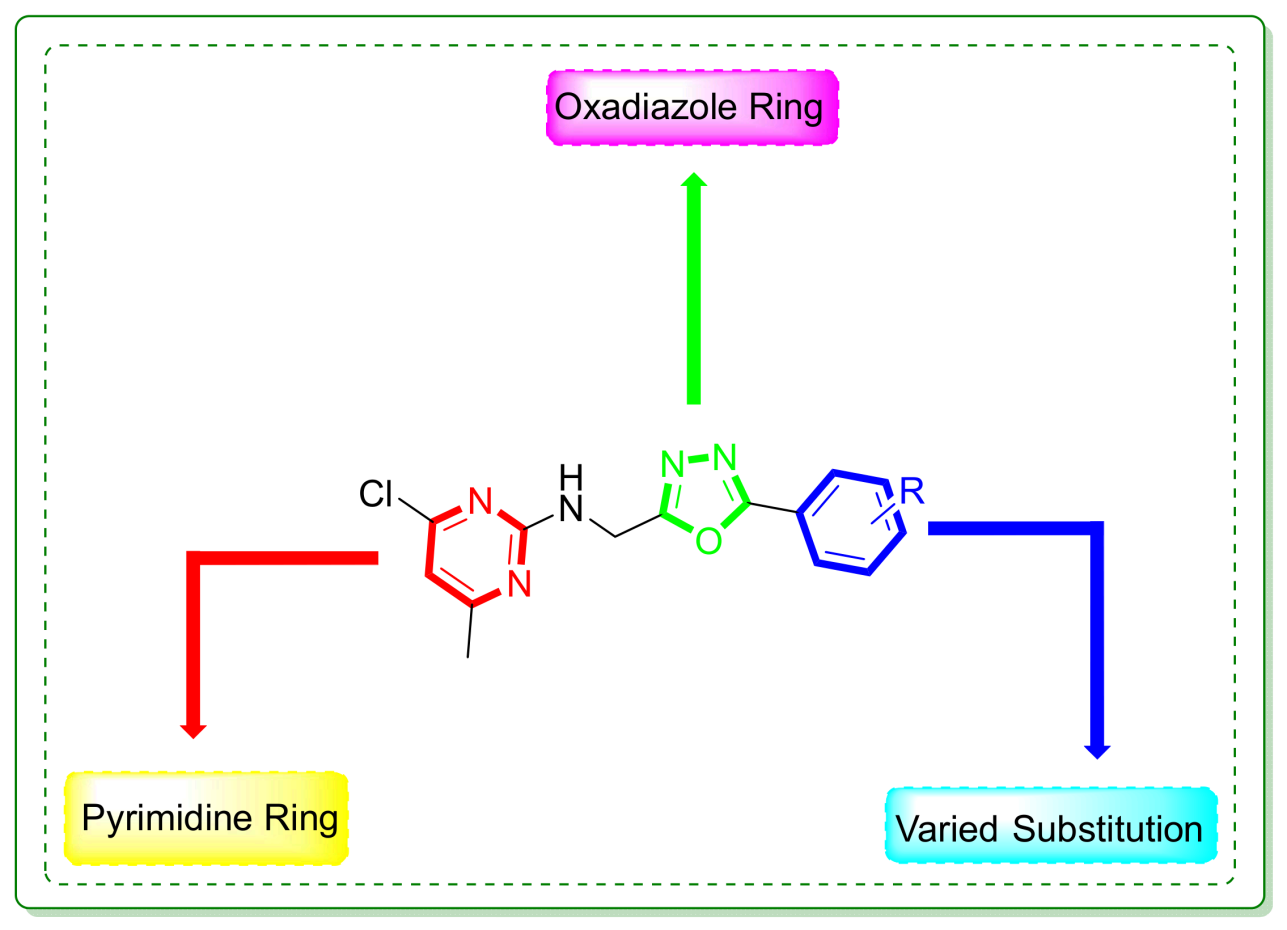
Represents the core structure of the synthesized compounds.

To identify potential diabetes treatments, a series of novel compounds was designed and successfully synthesized (1–10) (Figure 2) through a multi-step process. These molecules were then checked to see whether they block our target enzymes or not, i.e., α-amylase and α-glucosidase are involved in carbohydrate breakdown and blood sugar regulation, making them key targets for diabetes management. The analogs displayed a broad spectrum of inhibition against both enzymes, with their activity influenced by substituent groups attached to specific positions on the aromatic ring (Table 2). The electronic nature of these groups whether electron-withdrawing or donating played a pivotal role in modulating their potency. When compared to the reference drug Acarbose, the synthesized analogs exhibited notably lower IC_50_ values, indicating stronger inhibitory potential. For instance, variations in substitution patterns correlated with differences in activity, highlighting how structural modification could enhance efficiency. While all analogs proved significant enzyme inhibition, the interchanging between substituent position and electronic properties explained the observed variability in results.

#### Structure–Activity Relationship (SAR)

**Figure 3:**
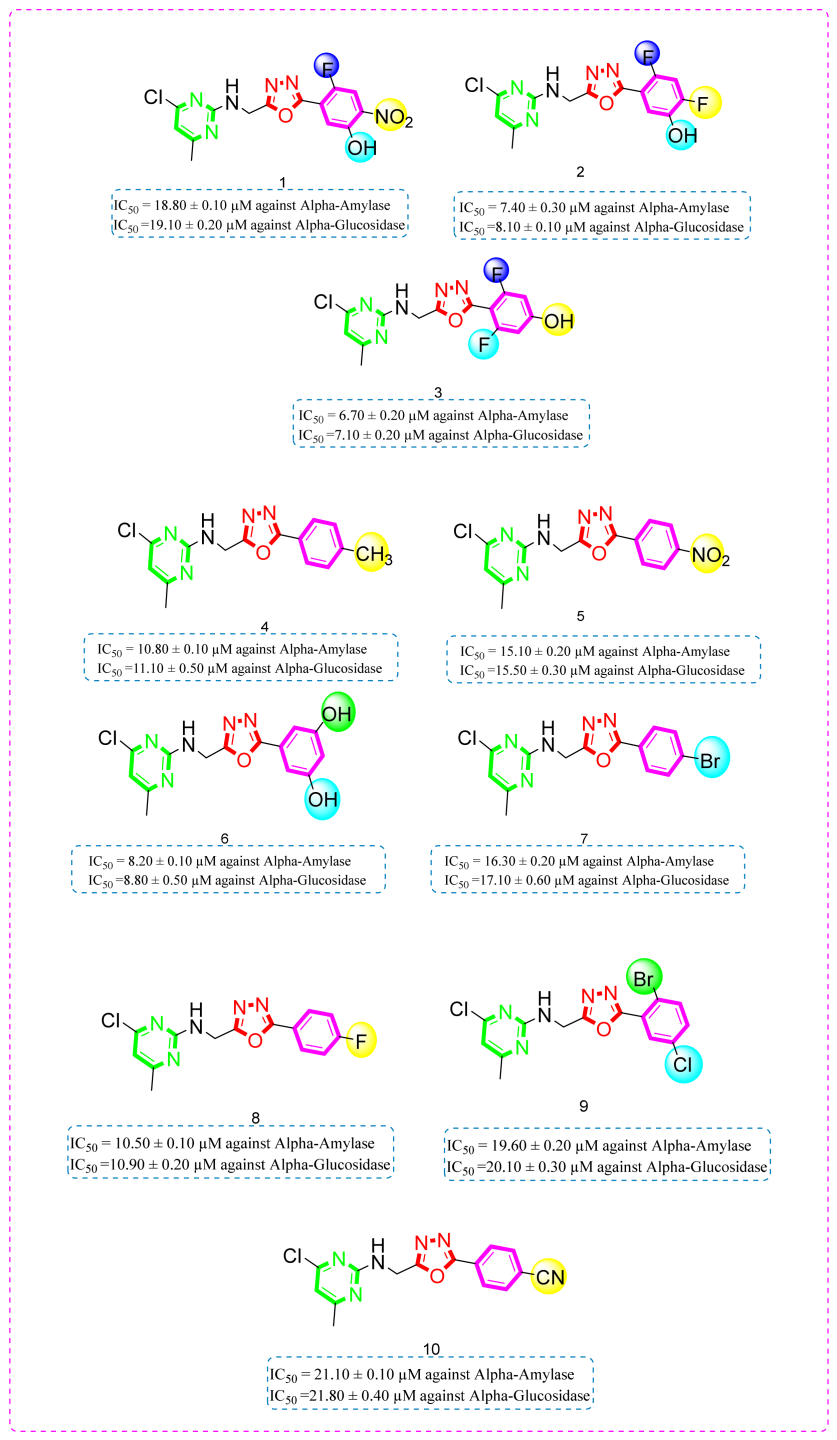
Represents the SAR study of synthesized analogs.

The structure–activity relationship (SAR) analysis indicates that most synthesized analogs exhibited measurable inhibitory activity against both α-amylase and α-glucosidase enzymes, suggesting that the pyrimidine–oxadiazole scaffold is generally favorable for enzyme inhibition (Figure 3). The observed differences in activity were evaluated mainly in terms of variations in functional group type and substitution pattern on the aromatic ring.

Overall, the activity trend suggests that electronic effects, hydrogen-bonding potential, and steric contributions of substituents collectively influence inhibitory potency; however, these interpretations are based on a limited compound set and should be considered as trend-based observations rather than definitive mechanistic conclusions.

Among all derivatives, analog 3 showed the most potent activity with IC_50_ = 6.70 ± 0.20 µM (α-amylase) and 7.10 ± 0.20 µM (α-glucosidase). Its relatively higher activity may be associated with the presence of fluorine and hydroxyl substituents, which likely contribute to favorable polarity and interaction capability, along with reduced steric demand compared to bulkier substituents. The docking analysis of compound **3** indicated that this compound forms stable interactions within the active site, including hydrogen bonding and favorable hydrophobic contacts with key catalytic residues, which may collectively contribute to its enhanced binding stability.

Analog 1 showed comparatively weaker activity, which may be related to its substitution pattern involving adjacent nitro and hydroxyl groups and ortho-positioned fluorine. These features could influence molecular conformation and interaction behavior, potentially reducing optimal binding efficiency relative to more active analogs.

Analog 2 exhibited improved inhibitory activity (IC_50_ = 7.40 ± 0.30 µM for α-amylase and 8.10 ± 0.10 µM for α-glucosidase), suggesting that its substitution pattern may be more favorable for enzyme interaction compared to analog 1. The presence of multiple fluorine substituents and a hydroxyl group may contribute collectively to its enhanced performance. Analog 2 also showed strong docking affinity, in agreement with its good inhibitory activity. Its binding mode suggested stable positioning within the enzyme active site, supported by multiple non-covalent interactions that may enhance complex stabilization.

Analog 4 showed moderate activity, indicating that simple hydrophobic substitution (e.g., methyl group) alone may be less effective in achieving strong inhibition compared to polar or highly electronegative substituents.

Analog 6 also demonstrated relatively strong activity (IC_50_ = 8.20 ± 0.10 µM and 8.80 ± 0.50 µM), suggesting that hydroxyl-containing derivatives may contribute positively to activity, likely due to increased polarity and interaction potential.

Overall, analogs bearing a balanced combination of polar functional groups and moderate steric bulk tended to show better inhibitory profiles, whereas compounds with fewer interactive functionalities or less favorable substitution patterns exhibited reduced activity. The remaining analogs (5, 8, 9, and 10) showed comparatively lower or moderate activity, supporting this general trend.

Importantly, due to the limited size of the dataset, detailed mechanistic interpretations (such as specific binding modes or energy contributions) cannot be conclusively established. Therefore, the SAR presented here should be interpreted as a preliminary structure–activity trend rather than a definitive mechanistic model.

### 2.3. In Vivo Preliminary Toxicological Assessment

**Figure 4:**
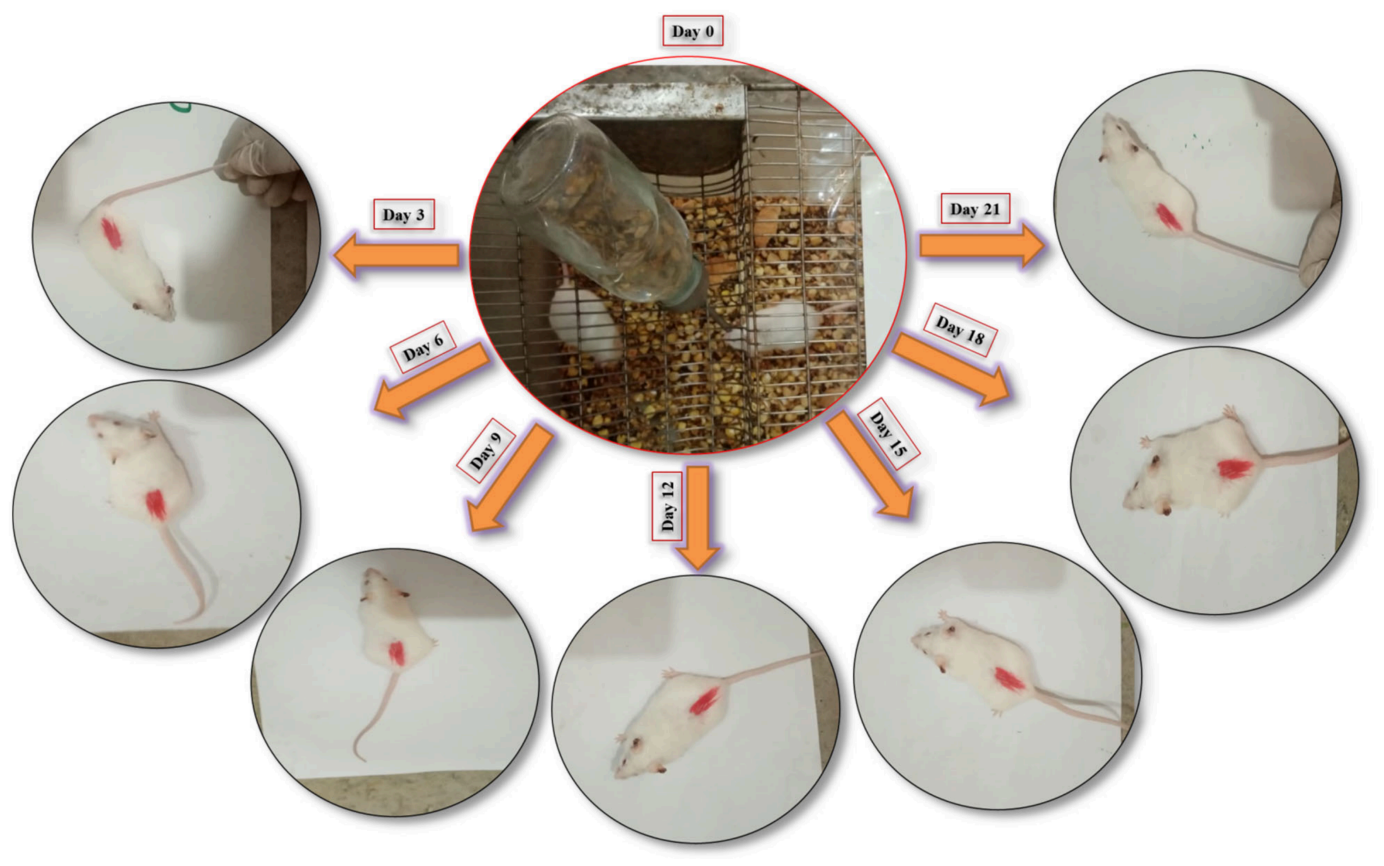
Comparative in vivo cytotoxicity evaluation of compound **3** in test group.

**Figure 5:**
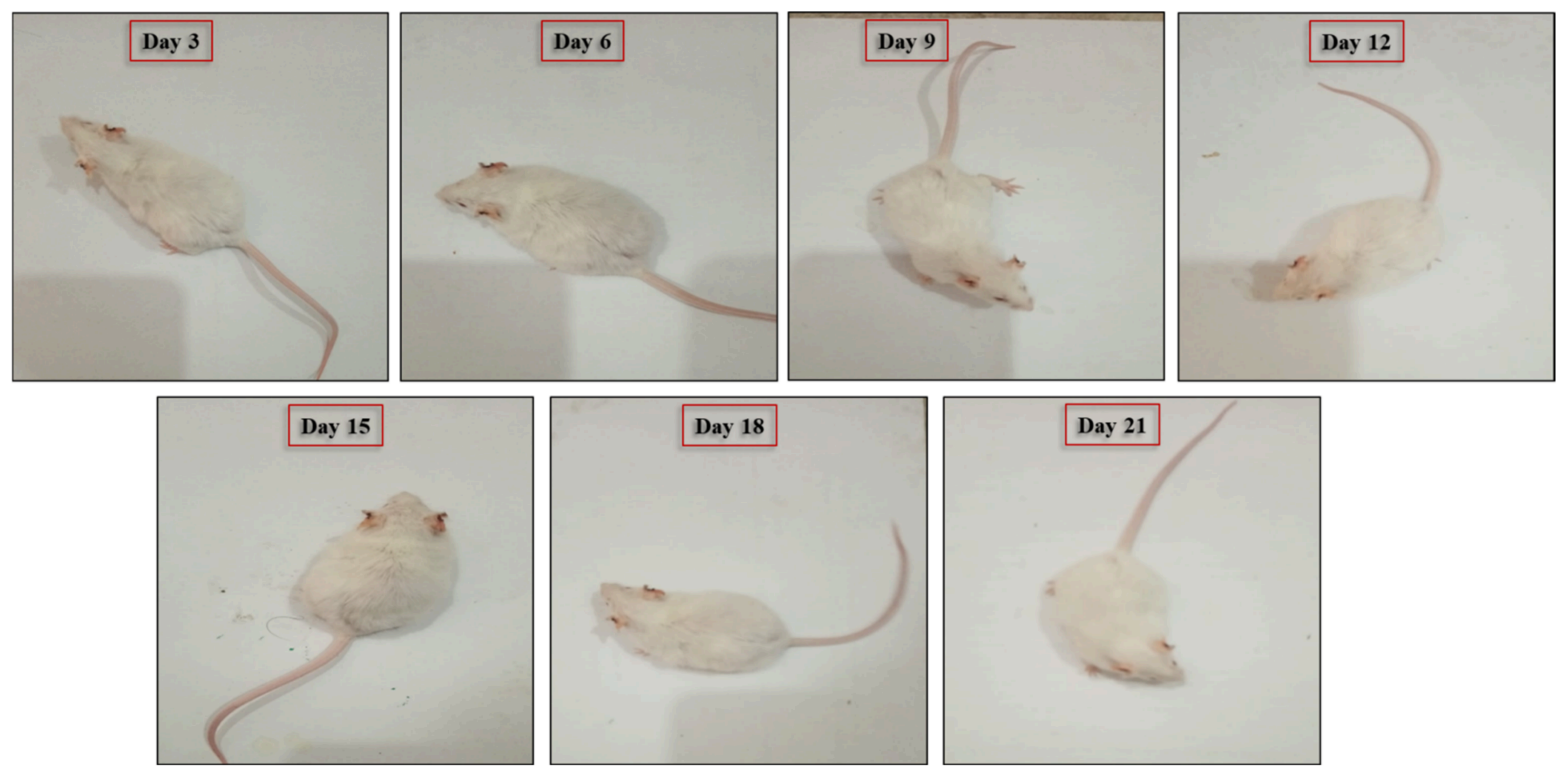
Comparative in vivo cytotoxicity evaluation of compound **3** in control group.

A sub-acute repeated-dose toxicity study was conducted in male Wistar rats to evaluate the in vivo preliminary toxicological profile of the selected lead compound (analog 3). Following a standardized acclimatization period, animals were randomly divided into a treatment cohort and a control cohort (Figure 4 and Figure 5). The test group received the compound via intraperitoneal injection once daily for a duration of 21 consecutive days. Dose selection strategies for preclinical studies recommend choosing a pharmacologically active dose below the maximum tolerated dose (MTD) to ensure safety while maintaining efficacy [29]. In accordance with these principles, 2.7 mg/kg was identified as an optimal dose that produced measurable biological effects without observable toxicity in preliminary studies.

It is important to note that the present in vivo investigation was conducted solely to evaluate the preliminary systemic safety of the developed compounds and not to assess their antidiabetic efficacy. The intraperitoneal route was selected to ensure controlled systemic exposure during toxicity screening. Given that α-amylase and α-glucosidase inhibitors are primarily intended for oral administration with localized gastrointestinal action, future studies will focus on oral dosing to establish pharmacological relevance and therapeutic potential.

Animals were continuously monitored throughout the experimental period for any signs of systemic toxicity or behavioral abnormalities. Daily observations included assessment of locomotor activity, posture, grooming behavior, feeding patterns, and responsiveness to environmental stimuli. Body weights were recorded at regular intervals to monitor general health status, and animals were examined twice daily for morbidity or mortality.

At study termination, blood samples were collected for comprehensive hematological and serum biochemical analysis. Major organs, including liver, kidneys, heart, brain, spleen, and lungs, were excised, weighed, and examined macroscopically, followed by histopathological evaluation to detect any treatment-associated structural alterations.

No mortality or treatment-related adverse effects were observed during the dosing period. All animals remained active and exhibited normal physiological behavior. Body weight gain patterns were comparable between treated and control groups. Biochemical, hematological, and histological findings collectively indicated an absence of systemic toxicity. Overall, analog 3 demonstrated good tolerability following repeated intraperitoneal administration, with no evidence of organ-specific cytotoxicity (Table 3, Table 4, Table 5, Table 6, Table 7, Table 8, Table 9, Table 10, Table 11 and Table 12).

#### 2.3.1. Hematological Evaluation

Hematological profiling revealed that repeated exposure to compound **3** did not induce any adverse effects on blood cell indices (Table 4). Erythrocytic parameters, including hemoglobin concentration, hematocrit, mean corpuscular indices, and red blood cell count, remained within established physiological limits and were comparable between treated and control animals, indicating preserved erythropoietic function.

Total leukocyte counts and differential leukocyte distribution showed only minor variations between groups, all of which fell within normal biological variability. Importantly, no evidence of leukopenia, leukocytosis, or inflammatory cell activation was detected, suggesting that the compound did not elicit immunotoxic or pro-inflammatory responses.

These findings confirm that the compound did not compromise hematological homeostasis or immune integrity under the experimental conditions.

**Table 4:** Hematological parameters following 21-day repeated-dose administration.

Parameter	Test Group	Control
Hemoglobin (g/L)	150.6 ± 6.2	146.1 ± 5.1
RBC (×10^12^/L)	8.8 ± 0.31	8.6 ± 0.27
Hematocrit (L/L)	0.47 ± 0.02	0.45 ± 0.03
WBC (×10^9^/L)	9.8 ± 1.3	10.2 ± 1.1
MCV (fL)	55.2 ± 1.4	54.1 ± 1.6
MCH (pg)	17.1 ± 0.5	16.6 ± 0.6
Lymphocytes (%)	72.5 ± 3.6	75.0 ± 4.2
Monocytes (%)	3.8 ± 0.9	3.5 ± 1.0
Eosinophils (%)	3.5 ± 1.2	3.2 ± 1.4
Basophils (%)	0.6 ± 0.4	0.7 ± 0.5

No statistically significant differences were observed (*p* > 0.05).

#### 2.3.2. Serum Biochemical Analysis

Serum biochemical investigations demonstrated that compound **3** did not adversely affect hepatic or renal function (Table 5). Levels of hepatic enzymes, including ALT and AST, remained within physiological reference ranges and did not differ significantly between treated and control groups. Protein metabolism markers showed only minimal fluctuations, with no concurrent elevation in bilirubin levels.

Renal function indices, including serum creatinine and electrolyte concentrations, remained stable throughout the study period. These findings indicate preserved kidney function and electrolyte balance.

Collectively, the biochemical data confirm the absence of treatment-related hepatic or renal toxicity following repeated administration.

**Table 5:** Serum biochemical parameters after repeated dosing.

Parameter	Test Group	Control
ALT (IU/L)	35 ± 6	37 ± 5
AST (IU/L)	70 ± 8	74 ± 9
Total protein (g/L)	56.2 ± 2.6	57.1 ± 2.3
Albumin (g/L)	36.0 ± 1.5	36.4 ± 1.6
Globulin (g/L)	20.2 ± 1.7	20.7 ± 1.8
Total bilirubin (µmol/L)	1.4 ± 0.3	1.3 ± 0.4
Creatinine (µmol/L)	17.6 ± 2.9	16.2 ± 3.1
Sodium (mmol/L)	144 ± 2	145 ± 3
Potassium (mmol/L)	4.9 ± 1.4	4.7 ± 1.2
Chloride (mmol/L)	103 ± 3	104 ± 2

#### 2.3.3. Absolute Organ Weight Analysis

Absolute organ weight analysis revealed no treatment-associated abnormalities (Table 6). The weights of metabolically sensitive organs, particularly the liver and kidneys, were comparable between treated and control animals. Minor variations observed among groups were within normal physiological ranges and were not supported by biochemical or histological evidence of toxicity.

**Table 6:** Absolute organ weights (g) after 21 days of treatment.

Group	Control	Test
Liver	1.312 ± 0.165	1.487 ± 0.142
Brain	0.432 ± 0.028	0.451 ± 0.026
Kidneys	0.358 ± 0.031	0.462 ± 0.038
Heart	0.182 ± 0.016	0.190 ± 0.015
Spleen	0.138 ± 0.019	0.131 ± 0.017
Lungs	0.195 ± 0.020	0.201 ± 0.023

#### 2.3.4. Relative Organ Weight Evaluation

Relative organ weight indices further supported the absence of systemic toxicity (Table 7). No significant differences were observed between treated and control groups across all assessed organs. Stable liver and kidney indices indicated preserved metabolic and excretory capacity, while unchanged brain and heart indices suggested no neurotoxic or cardiotoxic effects.

**Table 7:** Relative organ weights (%) following repeated-dose administration.

Group	Control	Test
Liver	5.12 ± 0.41	5.38 ± 0.33
Kidneys	1.51 ± 0.09	1.47 ± 0.11
Heart	0.59 ± 0.05	0.55 ± 0.06
Brain	1.50 ± 0.10	1.56 ± 0.12
Spleen	0.48 ± 0.06	0.45 ± 0.05
Lungs	0.83 ± 0.08	0.86 ± 0.07
Body Weight (g)	226 ± 14	232 ± 13

No statistically significant differences were detected (*p* > 0.05).

#### 2.3.5. Oxidative Stress and Antioxidant Status

To investigate whether repeated exposure to analog 3 induced oxidative damage, key oxidative stress and antioxidant defense markers were quantified in liver homogenates and serum (Table 8). Lipid peroxidation levels, assessed by malondialdehyde (MDA), showed no significant elevation in treated animals compared with controls. Similarly, endogenous antioxidant enzymes, including superoxide dismutase (SOD), catalase (CAT), and reduced glutathione (GSH), remained within normal physiological ranges.

These results indicate that the compound did not disrupt cellular redox balance or induce oxidative stress-mediated cytotoxicity.

**Table 8:** Oxidative stress and antioxidant biomarkers following repeated-dose administration.

Parameter	Test Group	Control
MDA (nmol/mg protein)	1.82 ± 0.21	1.76 ± 0.19
SOD (U/mg protein)	8.9 ± 0.7	9.1 ± 0.6
Catalase (U/mg protein)	52.4 ± 4.1	53.8 ± 4.6
GSH (µmol/g tissue)	6.7 ± 0.5	6.9 ± 0.6

No statistically significant differences were detected (*p* > 0.05).

#### 2.3.6. Pro-Inflammatory Cytokine Profiling

To assess potential inflammatory or immune-mediated toxicity, serum levels of key pro-inflammatory cytokines were quantified (Table 9). Concentrations of TNF-α, IL-6, and IL-1β remained comparable between treated and control animals, indicating the absence of systemic inflammatory responses.

These findings are consistent with stable leukocyte counts observed in hematological analysis.

**Table 9:** Serum inflammatory cytokine levels in treated and control rats.

Parameter	Test Group	Control
TNF-α (pg/mL)	22.6 ± 3.4	21.9 ± 3.1
IL-6 (pg/mL)	18.4 ± 2.8	19.1 ± 2.5
IL-1β (pg/mL)	11.2 ± 1.6	10.8 ± 1.7
CRP (mg/L)	0.89 ± 0.12	0.86 ± 0.10

#### 2.3.7. Behavioral and Neurotoxicity Screening

A functional observational battery was employed to identify potential neurobehavioral effects (Table 10). Parameters such as locomotor activity, grip strength, sensory response, and reflex integrity were scored semi-quantitatively. No deviations from normal behavior were observed in treated animals.

These observations, combined with stable brain weights and histology, suggest the absence of neurotoxic liability.

**Table 10:** Behavioral and neurological assessment scores.

Parameter	Test Group	Control
Locomotor activity	Normal	Normal
Posture & gait	Normal	Normal
Grip strength	Normal	Normal
Startle response	Intact	Intact
Pain response	Normal	Normal
Tremors/convulsions	Absent	Absent

#### 2.3.8. Histopathological Scoring of Major Organs

To enhance objectivity in histological interpretation, a semi-quantitative scoring system was applied to major organs (Table 11). Scores were assigned based on cellular degeneration, inflammation, necrosis, and vascular changes (0 = absent, 1 = minimal, 2 = mild, 3 = moderate).

No pathological alterations were detected in treated animals, with scores comparable to controls.

**Table 11:** Histopathological lesion scoring of vital organs.

Organ	Test Group	Control
Liver	0.2 ± 0.1	0.2 ± 0.1
Kidneys	0.1 ± 0.1	0.1 ± 0.1
Heart	0.0 ± 0.0	0.0 ± 0.0
Brain	0.0 ± 0.0	0.0 ± 0.0
Spleen	0.1 ± 0.1	0.1 ± 0.1
Lungs	0.2 ± 0.1	0.2 ± 0.1

#### 2.3.9. Endocrine and Metabolic Indicators

To exclude endocrine disruption or metabolic imbalance, serum glucose and lipid profile parameters were evaluated (Table 12). No significant differences were observed between groups, indicating preserved metabolic homeostasis.

**Table 12:** Metabolic and endocrine-related serum parameters.

Parameter	Test Group	Control
Glucose (mmol/L)	6.1 ± 0.4	6.0 ± 0.5
Total cholesterol (mmol/L)	1.82 ± 0.18	1.79 ± 0.20
Triglycerides (mmol/L)	0.93 ± 0.11	0.90 ± 0.12
HDL-cholesterol (mmol/L)	0.72 ± 0.08	0.70 ± 0.07
LDL-cholesterol (mmol/L)	0.84 ± 0.10	0.86 ± 0.09

#### 2.3.10. Assessment of Observed Safety Profile

No mortality, abnormal behavior, or significant changes in hematological, biochemical, oxidative stress, inflammatory, organ weight, or histopathological parameters were observed following repeated intraperitoneal administration of analog 3 at a dose of 2.7 mg/kg/day for 21 days. These findings indicate that the compound was well tolerated under the tested experimental conditions.

#### 2.3.11. Integrated Toxicological Conclusion

Incorporation of oxidative stress profiling, cytokine analysis, neurobehavioral screening, histopathological scoring, and metabolic assessment significantly strengthens the toxicological evaluation of analog 3. Collectively, these findings demonstrate that the compound is well tolerated in the short term; these findings should be interpreted with caution. Comprehensive toxicological investigations, including multiple dose levels, extended treatment durations, larger cohorts, and detailed histopathological analyses, are necessary to fully establish the safety profile. Accordingly, the present data provide an initial indication of tolerability rather than definitive evidence of long-term safety or clinical suitability.

### 2.4. Enzyme Kinetics

#### 2.4.1. Assessment of Inhibitory Concentration and Inhibition Efficacy

**Figure 6:**
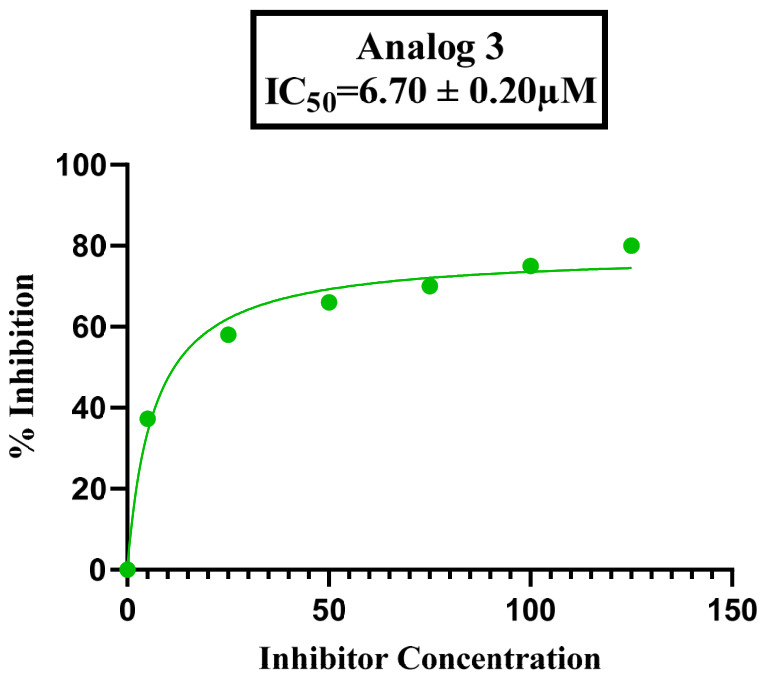
Inhibition curve illustrating the effect of analog 3 on α-amylase activity.

**Figure 7:**
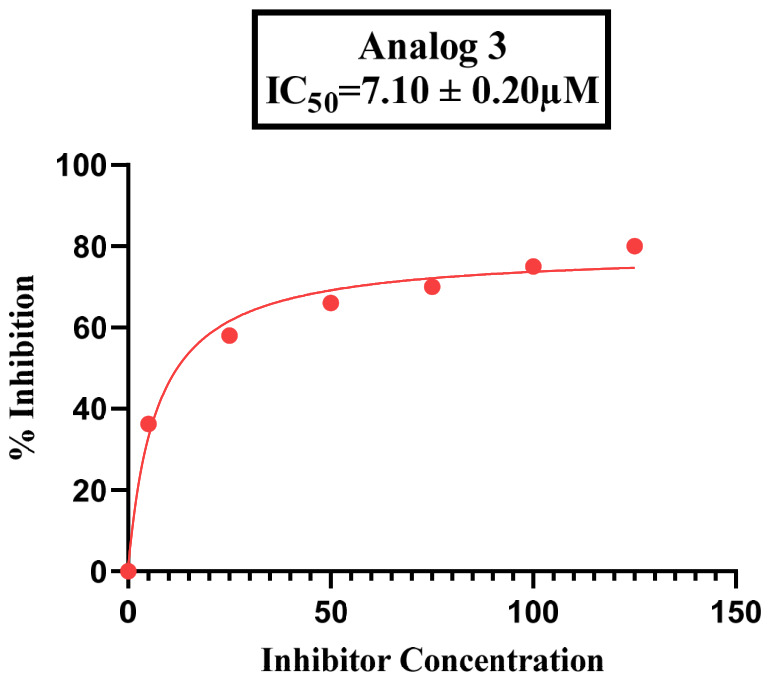
Inhibition curve illustrating the effect of analog 3 on α-glucosidase activity.

To confirm the validity of the in vitro enzymatic activity findings, we tested a group of promising inhibitors through an inhibition rate analysis. The investigation specifically examined both competitive analogs and bioactive candidates identified during the initial in vitro screening. Among these, analog 3 stood out for its ability to disrupt enzyme active sites through targeted binding, showing remarkable effectiveness at micromolar concentrations. As illustrated in Figure 6, Figure 7 and Figures S1 and S2, the data underscores analog 3 notable potency in suppressing alpha-amylase and alpha-glucosidase activity compared to other tested compounds, solidifying its status as the most impactful inhibitor in the series.

**Figure 8:**
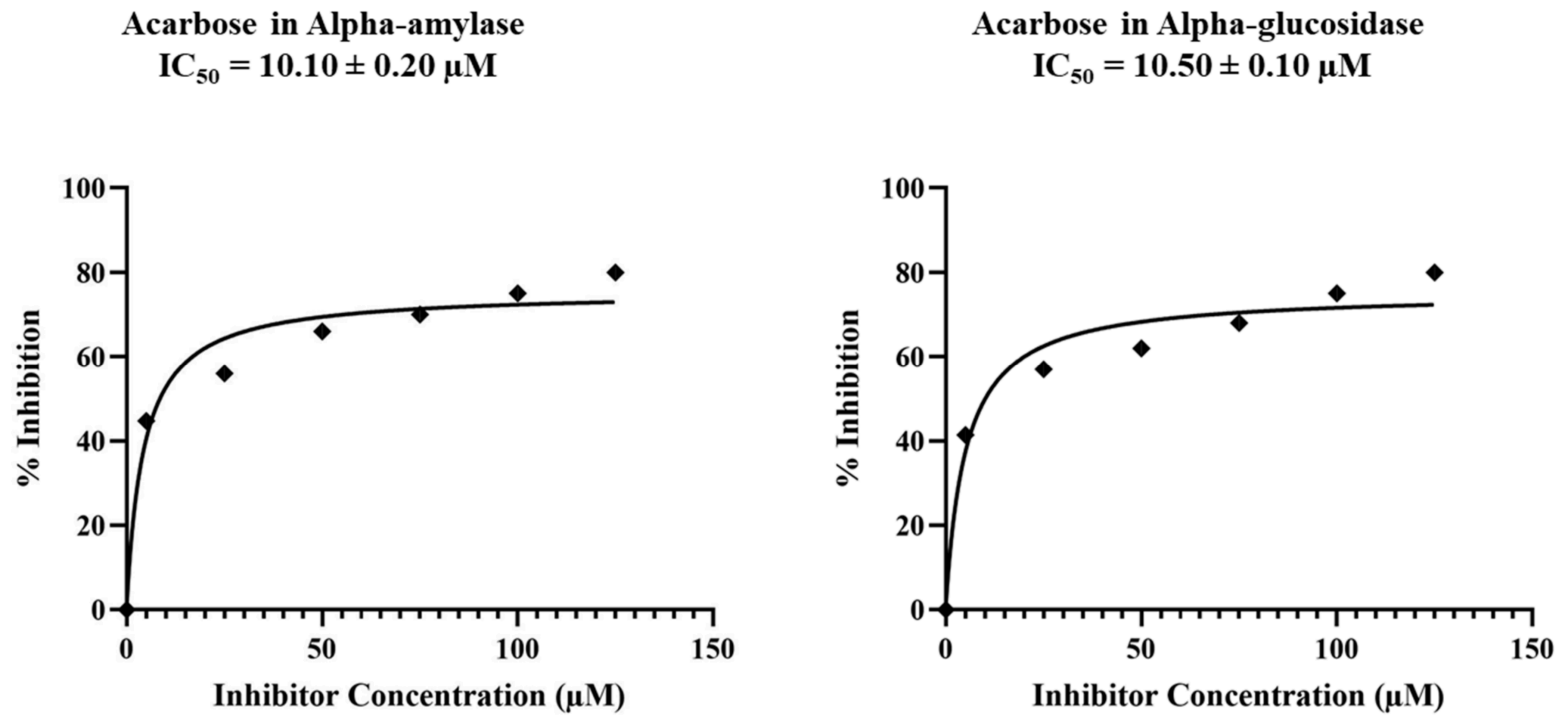
Inhibition curve illustrating the effect of acarbose on α-amylase and α-glucosidase activity.

The inhibition curves were analyzed with appropriate statistical rigor to ensure reproducibility and transparency of the results. All experiments were performed in triplicate (n = 3, independent assays). The dose–response curves were fitted using a nonlinear regression model based on the four-parameter logistic equation. For statistical comparison, the inhibitory activities of the most potent compounds **2** and **3** were evaluated against acarbose (Figure 8).

#### 2.4.2. Kinetic Analysis of Enzyme Inhibition

To assess the inhibitory behavior of lead compounds **2** and **3**, kinetic studies were performed to measure their suppression rates. The inhibition mechanism of the selected analogs was evaluated using Lineweaver–Burk double reciprocal plots (1/V vs. 1/[S]), which provide a reliable approach for distinguishing different types of enzyme inhibition. The kinetic parameters including k_m_, V_max_ and k_i_ are given in Table 13.

**Figure 9:**
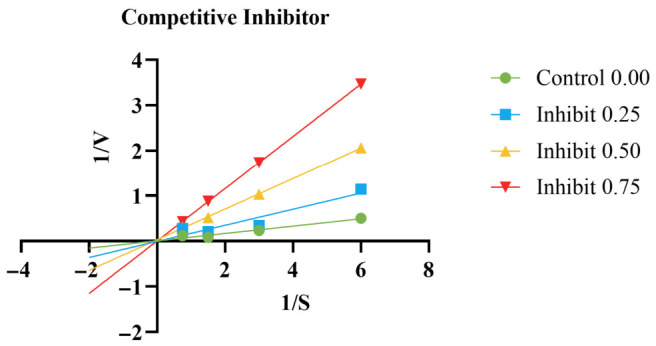
Enzymatic reaction kinetic study of analog 3, as competitive inhibitor.

As shown in Figure 9, analog 3 exhibits a typical pattern of competitive inhibition, where all lines intersect at a common y-intercept, indicating that the maximum reaction velocity (Vmax) remains unchanged while the apparent Km increases with increasing inhibitor concentration. This behavior suggests that analog 3 competes with the substrate for binding at the enzyme active site, thereby reducing substrate affinity without affecting the catalytic turnover at saturation.

**Figure 10:**
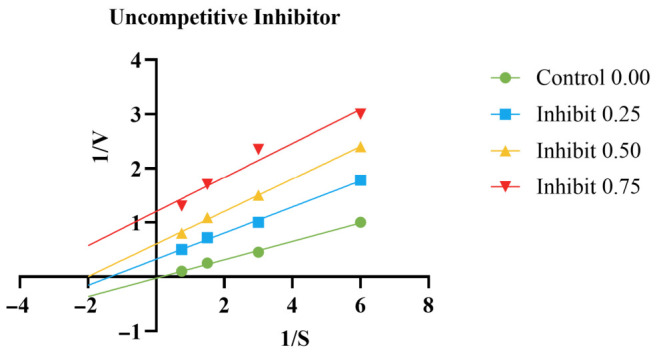
Enzymatic reaction kinetic study of analog 2, as un-competitive inhibitor.

In contrast, analog 2 demonstrates an uncompetitive inhibition pattern, as evidenced by the set of parallel lines observed in the Lineweaver–Burk plot (Figure 10). This indicates a simultaneous decrease in both Km and Vmax, confirming that the inhibitor binds preferentially to the enzyme–substrate complex rather than the free enzyme. The proportional reduction in these kinetic parameters reflects stabilization of the enzyme–substrate complex and suppression of product formation. Overall, these results clearly distinguish the two inhibition mechanisms, with analog 3 acting as a competitive inhibitor and analog 2 as an uncompetitive inhibitor, providing important insight into their enzyme interaction profiles.

### 2.5. Molecular Docking

Molecular docking studies were performed to investigate the binding interactions [30,31,32,33,34] of the synthesized potent compounds **2** and **3** with α-amylase and α-glucosidase enzymes. Molecular docking studies were performed using AutoDock Vina 1.2.7 to evaluate the binding affinity of the synthesized compounds toward the target enzymes. The crystal structures of the target proteins were retrieved from the Protein Data Bank (PDB IDs: 1B2Y for α-amylase and 3W37 for α-glucosidase). Protein structures were prepared by removing co-crystallized ligands, water molecules, and non-essential heteroatoms, followed by the addition of polar hydrogen atoms and assignment of Kollman charges. Protonation states of amino acid residues were adjusted to physiological pH conditions to ensure biologically relevant binding-site representation. Ligand structures were drawn and energy-minimized prior to docking using standard geometry optimization to obtain stable lowest-energy conformations. The exhaustiveness parameter was set to 8, with a maximum of 10 binding modes generated for each ligand.

The grid box was centered on the active site region corresponding to the co-crystallized ligand binding pocket. For α-amylase (PDB ID: 1B2Y), the grid box was defined with center coordinates x = 18.45, y = 25.32, z = 31.78, and dimensions of 60 × 60 × 60 grid points with a grid spacing of 0.375 Å, ensuring complete coverage of the catalytic binding pocket. For α-glucosidase (PDB ID: 3W37), the grid box was centered at x = 12.64, y = 34.91, z = 22.57, with identical dimensions of 60 × 60 × 60 grid points and a grid spacing of 0.375 Å, covering the full active-site cavity and catalytic residues.

To validate the docking protocol, co-crystallized ligands were re-docked into their respective binding sites, and RMSD values below 2.0 Å confirmed the reliability of the docking procedure. Additionally, acarbose was used as a standard reference inhibitor for comparative binding affinity analysis. Docking results were ranked based on binding free energy. The best-ranked poses were selected for detailed interaction analysis. Visualization and interaction profiling were performed using PyMOL and BIOVIA Discovery Studio Visualizer (DSV) (2024) to identify hydrogen bonding, hydrophobic interactions, and key active-site residues. Finally, binding energies of all compounds, along with the reference standard, were tabulated for comparative structure–activity relationship (SAR) analysis and correlation with experimental enzyme inhibition data.

**Figure 11:**
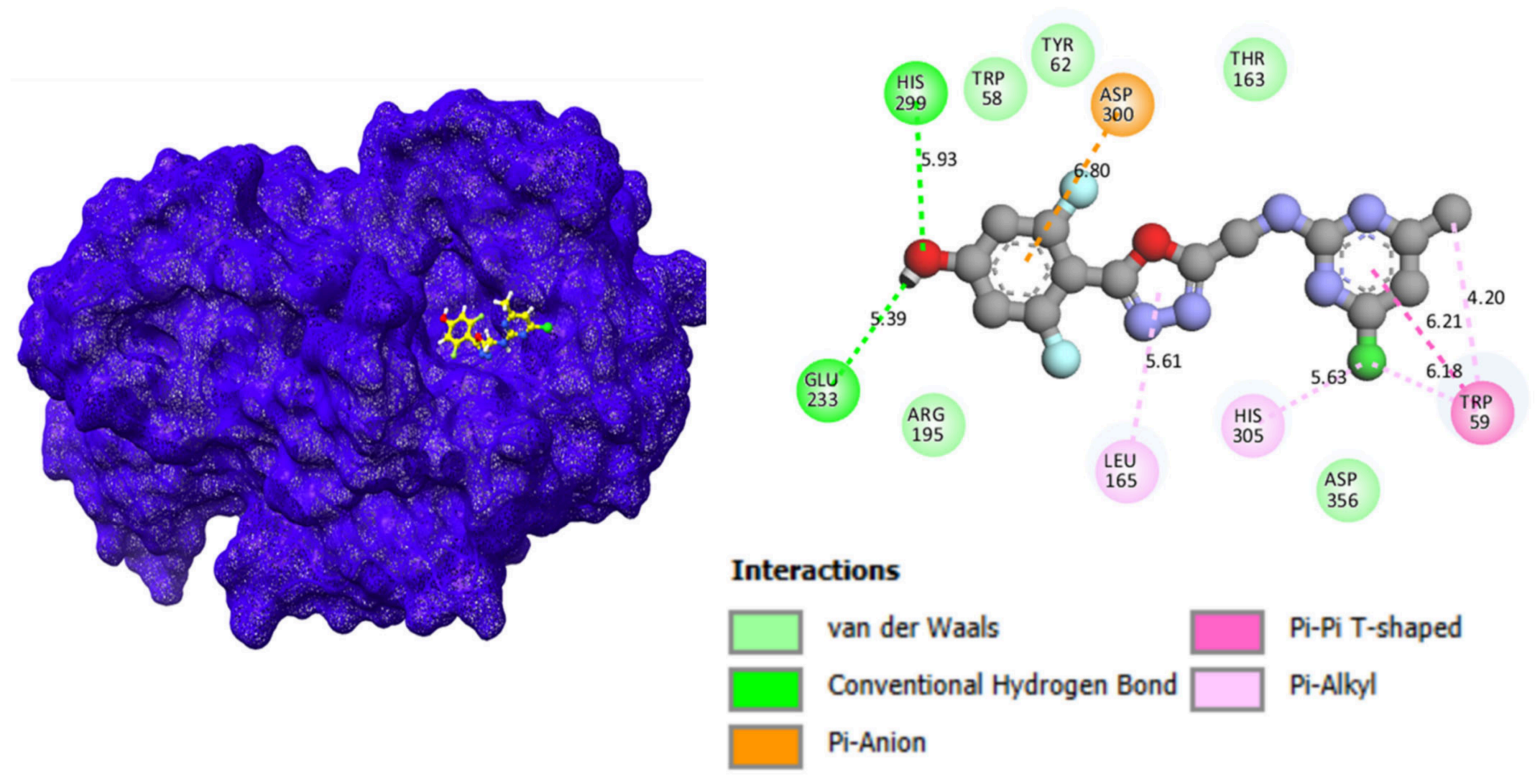
Binding interactions of compound **3** with α-amylase at the receptor binding region.

**Figure 12:**
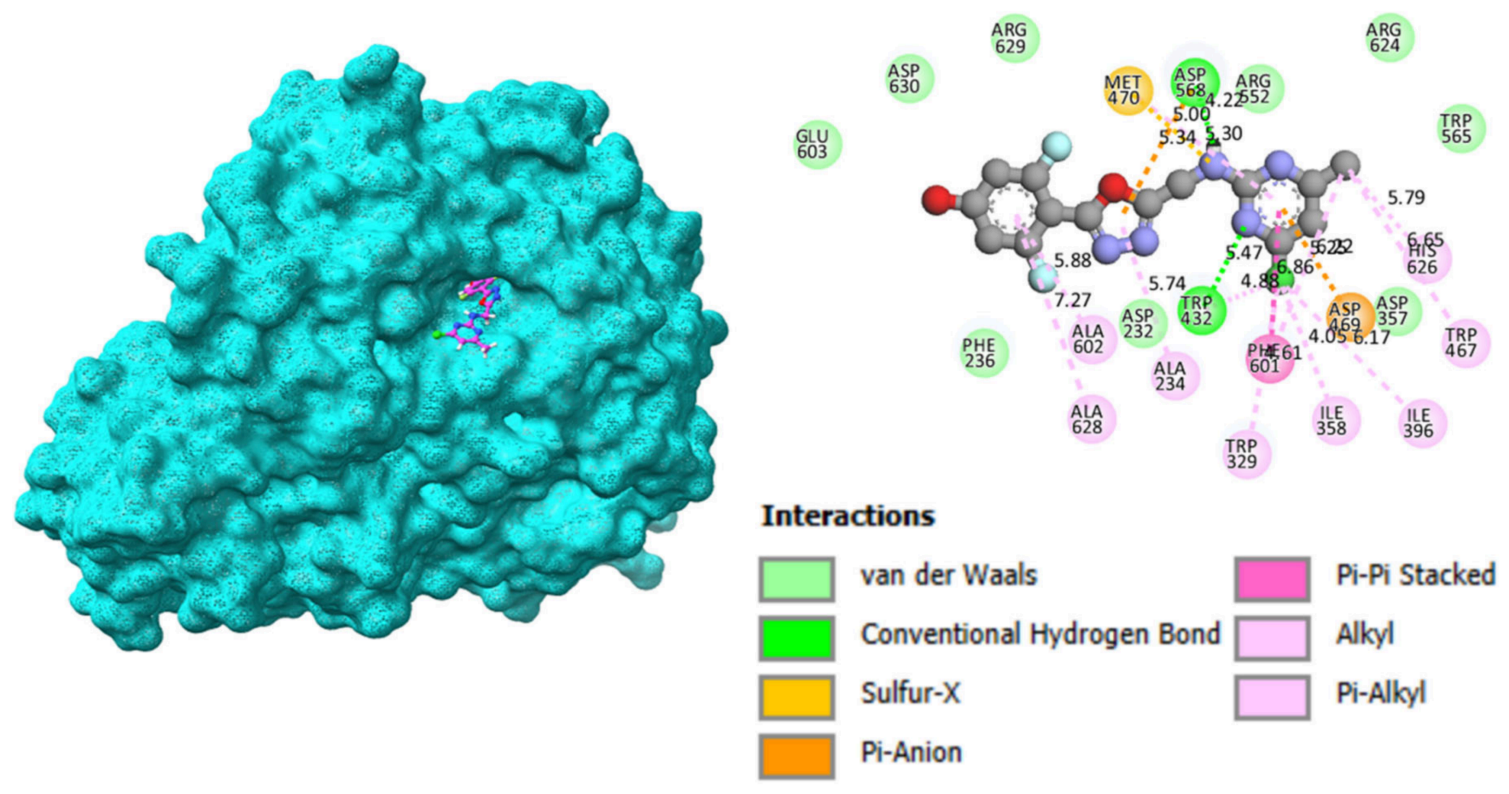
Binding interactions of compound **3** with α-glucosidase at the receptor binding region.

**Figure 13:**
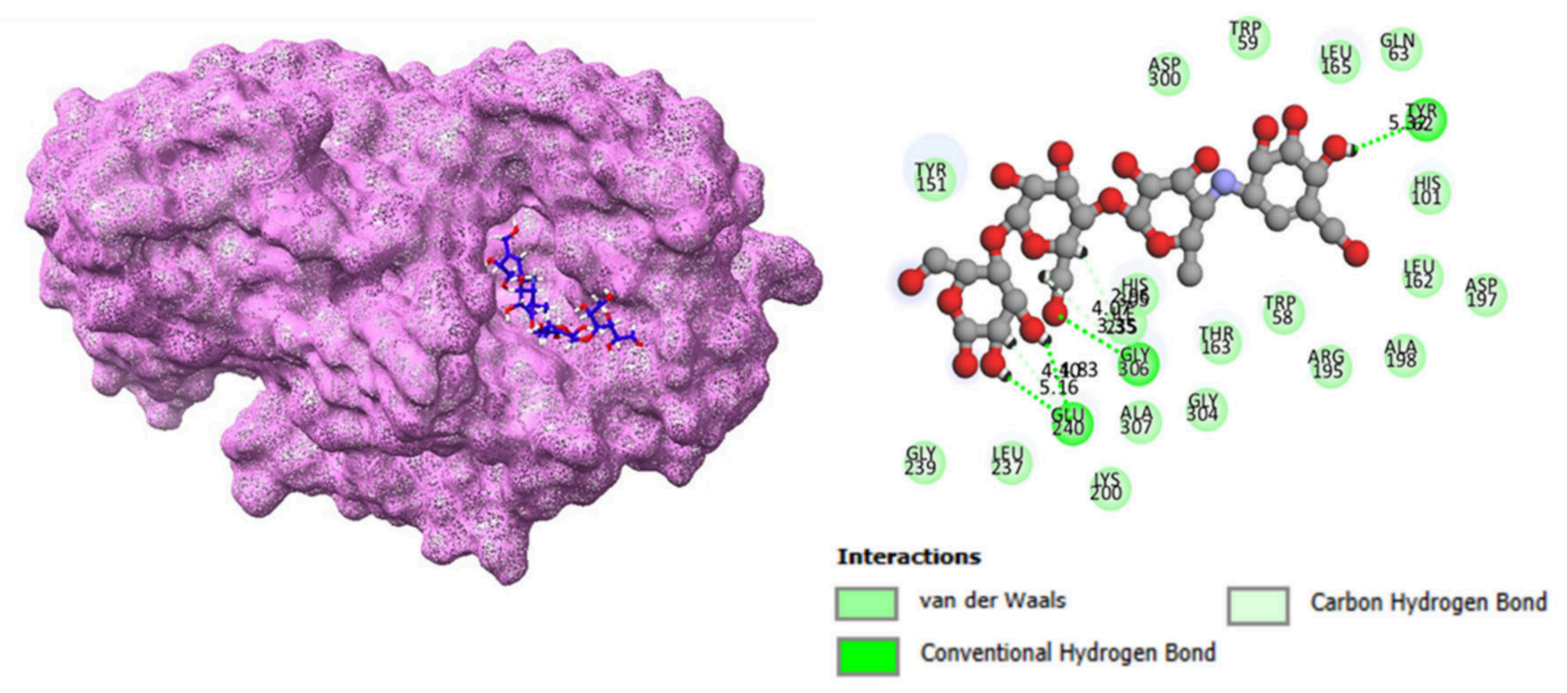
Binding interactions of acarbose with α-amylase at the receptor binding region.

**Figure 14:**
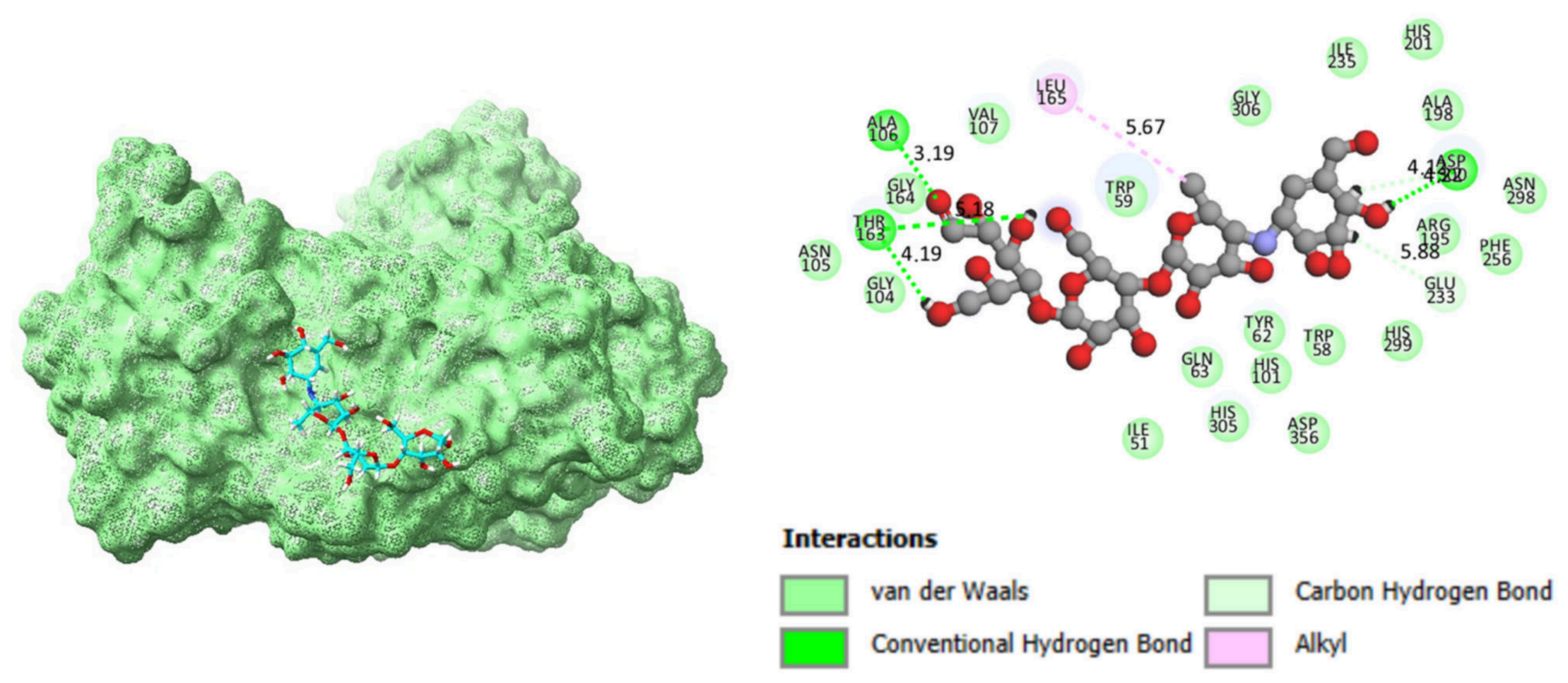
Binding interactions of acarbose with α-glucosidase at the receptor binding region.

Incorporating different substituents enhanced the enzyme-blocking effects of the compounds. Our computational studies revealed critical insights into the key interactions found in these compounds with the amino acid residues comprising the enzymes’ active sites. The inhibitory potency of these compounds was influenced by multiple interaction types. Notably, analog 3 displayed distinct binding modes attributable to its variable binding orientations. The pyrimidine-based oxadiazole moiety formed crucial side-chain donor interactions, while the ligand’s acidic component participated in stabilizing electron-donor interactions with the target site. The exposed phenyl ring of the oxadiazole moiety demonstrated pronounced electron-rich character, which facilitated more efficient binding with the target enzymes. Additionally, the analog’s hydrophobic and basic characteristics, combined with its donor/acceptor interaction profile, established its structural framework as particularly favorable for modulating the enzymatic activity. Results are depicted in Figure 11, Figure 12 and Figures S3 and S4. Binding interactions of potent compound **2** and **3** in comparison to standard drug acarbose (Figure 13 and Figure 14) are given in Table 14. RMSD values below 2.0 Å are given in Table 15.

### 2.6. DFT Analysis

#### 2.6.1. Molecular Electrostatic Potential (MEP)

**Figure 15:**
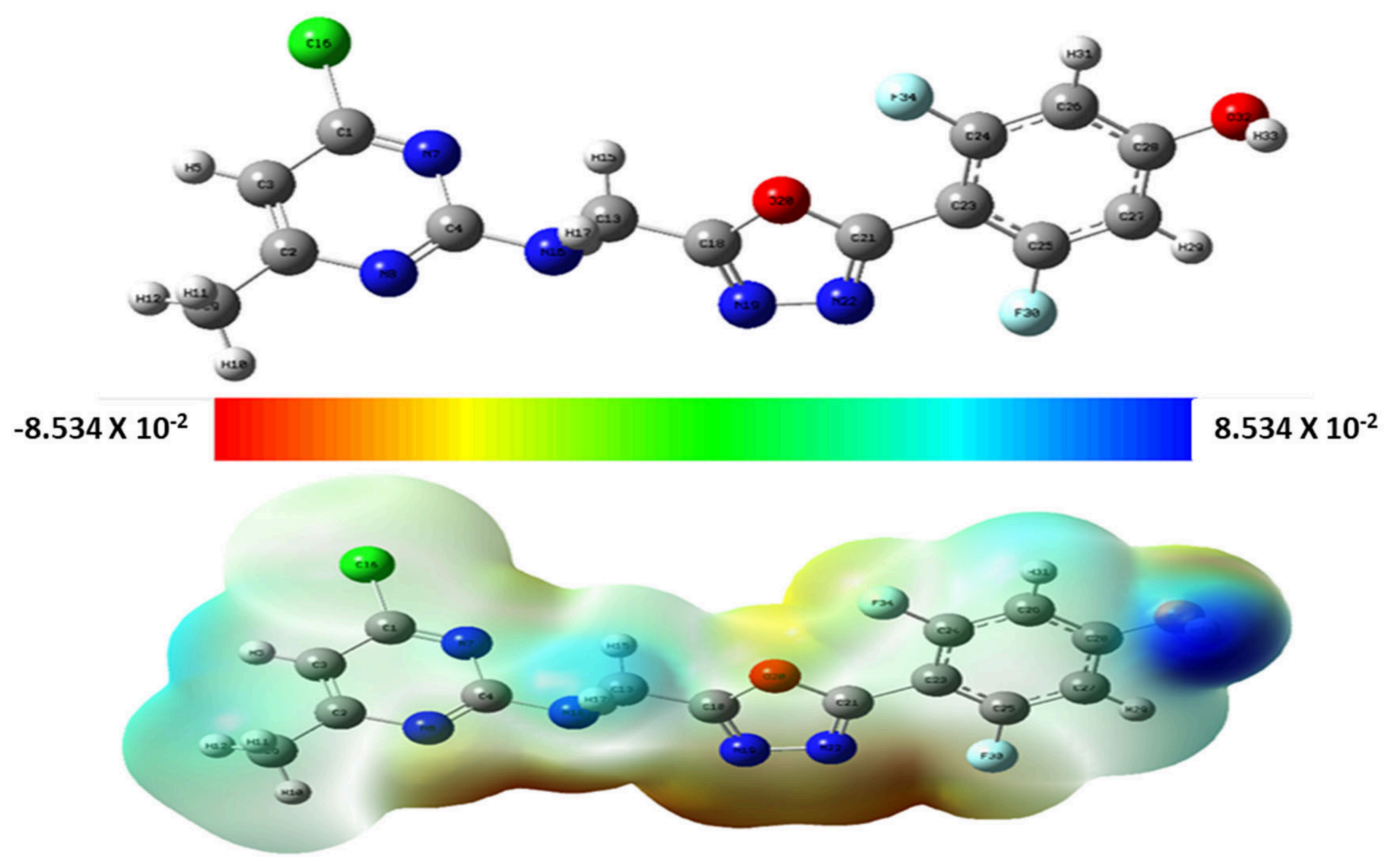
The MEP profile and electronic configuration of analog 3 reveal distinct electrophilic and nucleophilic zone.

Molecular electrostatic potential (MEP) chart act like a “charge blueprint” for molecules, showing how electrostatic forces are distributed across their surfaces [35,36,37,38,39]. Areas in green represent regions with weaker charge density (positive potential), which tend to attract electron seeking reactions. In contrast, zones shaded red to yellow highlight the flash point of high electron density (negative potential), marking where nucleophilic reactions are most likely to occur. When analyzing compounds **2** and **3**, their distinct functional groups and electron-sharing patterns created unique electrostatic fingerprints. Analyses revealed that analogs with electronegative atoms showed amplified electron density at nucleophilic sites, while electrophilic regions were notably extra in charge. These trends, illustrated in Figure 15 and Figures S5 and S6, help link the gap between a compound’s structure and its reactivity, revealing why certain groups show specific chemical behaviors.

The molecular electrostatic potential (MEP) surfaces of analog 2 and analog 3 were generated to visualize the distribution of electronic charge and to identify potential interaction sites within the molecules. The MEP maps provide a qualitative representation of electron-rich (negative potential) and electron-deficient (positive potential) regions, which are relevant for understanding intermolecular interactions with biological targets.

In both analogs, regions of negative electrostatic potential (red–yellow zones) are primarily localized around electronegative atoms such as oxygen and fluorine, indicating potential sites for electrophilic interactions and hydrogen-bond acceptance. Conversely, positive potential regions (green–blue zones) are mainly distributed over hydrogen atoms and less electronegative regions of the molecular framework, suggesting possible hydrogen-bond donor sites.

A comparative analysis shows that analog 3 exhibits a more continuous and extended electron-rich region, particularly around the substituted aromatic ring and heterocyclic core. This enhanced electron density distribution may facilitate stronger electrostatic and hydrogen-bonding interactions within enzyme active sites. In contrast, analog 2 displays a relatively less delocalized charge distribution, which may correspond to comparatively weaker interaction potential.

#### 2.6.2. Frontier Molecular Orbital (FMO) Analysis

To explore how the synthesized compounds interact chemically, we focused on the frontier molecular orbitals (FMOs) of the most promising analogs [40,41,42]. This involved studying two critical regions, i.e., the HOMO (highest occupied molecular orbital), which represents the molecule’s electron-rich zones, and the lowest unoccupied molecular orbital (LUMO), essentially its electron-poor zones. The energy difference between these orbitals which is mainly called HOMO−LUMO gap acts like a reactivity parameter, a smaller gap means electrons can jump more easily between orbitals, making the compound more reactive. A high HOMO energy signals a molecule ready to donate electrons, while a low LUMO energy suggests it is ready to accept electrons. In compounds **2** and **3**, structural modification such as adding electronegative groups pushed HOMO energy higher, turning them into electron-donating powerhouses. Conversely, features that lowered LUMO energy made these molecules ready to take electrons.

For analog 3, the HOMO is predominantly localized over the heterocyclic core and substituted aromatic ring, indicating that these regions may participate in electron donation during intermolecular interactions. The LUMO is distributed over electron-deficient regions, suggesting potential sites for electron acceptance. The calculated orbital energies are:

- HOMO = −0.35036 eV
- LUMO = −0.6685 eV
- ΔE = 0.31814 eV

The relatively small HOMO–LUMO gap indicates moderate electronic flexibility, which may facilitate interaction with biological targets through charge transfer mechanisms.

For analog 2, a similar orbital distribution pattern is observed; however, the degree of orbital delocalization appears comparatively limited. This may contribute to slightly reduced interaction efficiency relative to analog 3, in agreement with experimental observations.

**Figure 16:**
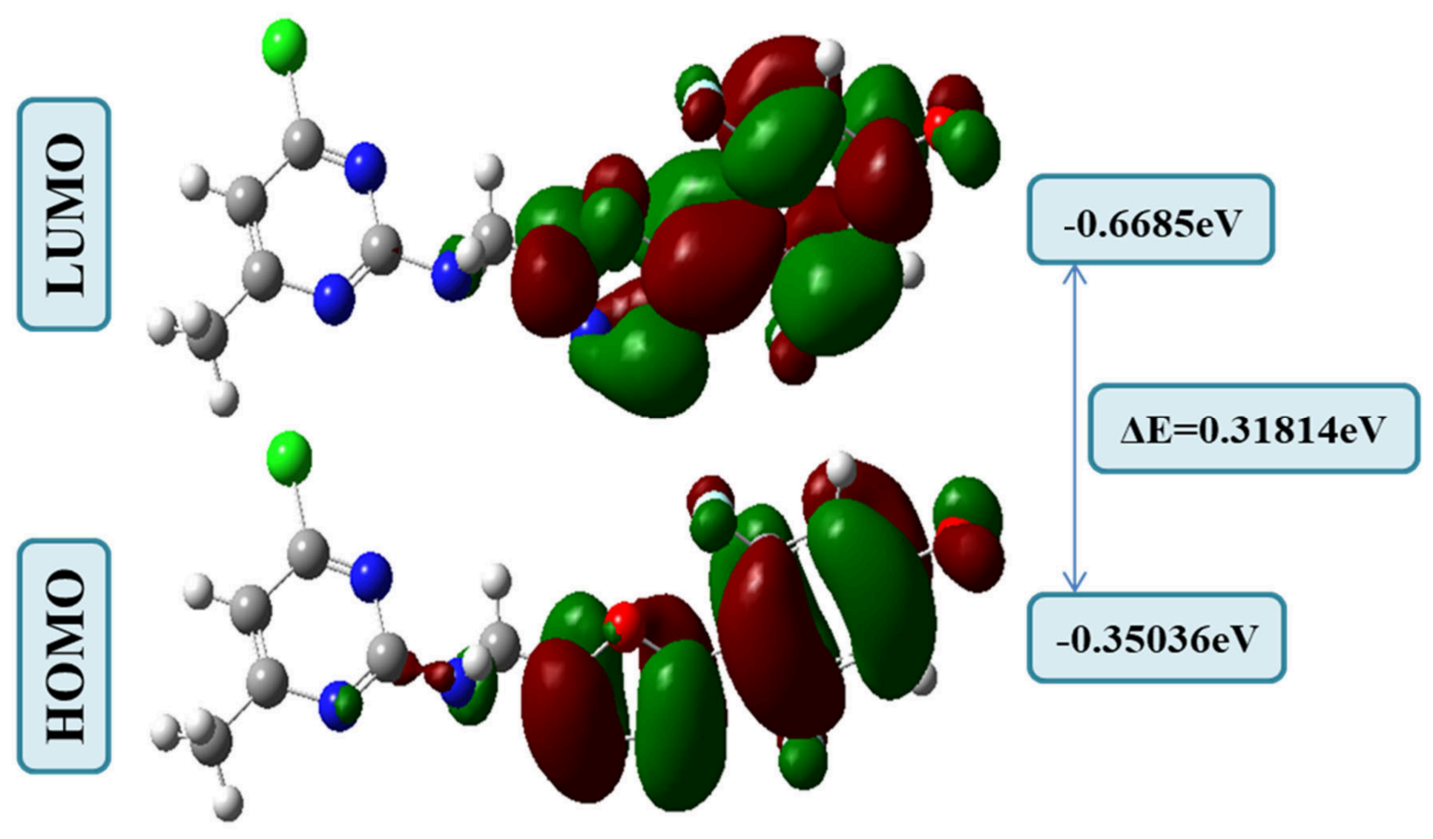
FMO analysis of analog 3.

Overall, the FMO analysis suggests that electronic distribution and orbital localization influence interaction potential with enzyme active sites. These patterns, visualized in Figure 16 and Figure S6, highlight how structural features influence the reactivity and stability of compounds **2**, **3** and **6**, offering critical guidance for refining these molecules in future studies.

### 2.7. ADMET Analysis

**Figure 17:**
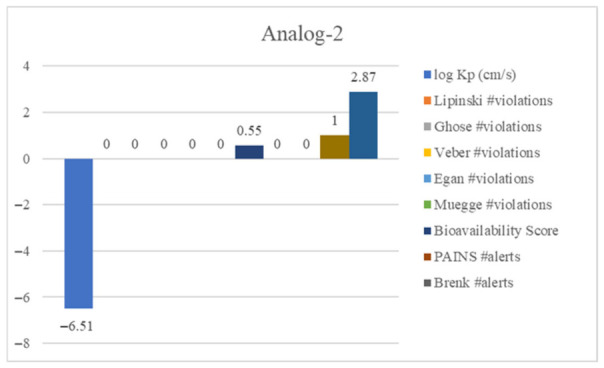
ADMET profile of analog 2.

**Figure 18:**
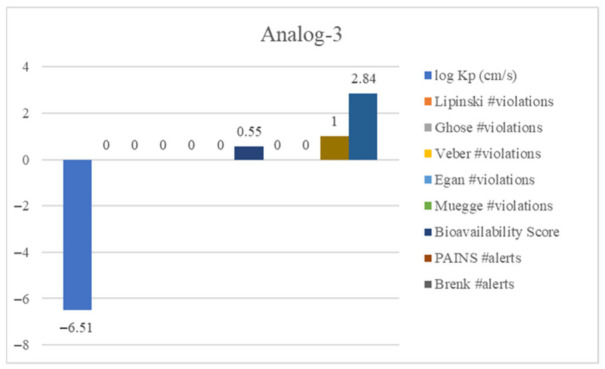
ADMET profile of analog 3.

After synthesizing a drug, researchers’ first priority is ADMET analysis, a critical evaluation of absorption, distribution, metabolism, excretion, and toxicity. Using the Swiss ADME online tool, we evaluated the drug-like properties of the newly synthesized pyrimidine-oxadiazole derivatives through ADMET profiling, aiming to determine their suitability as potential therapeutic agents. ADMET results of compounds **2** and **3** are presented in Figure 17 and Figure 18.

Both analogs exhibited acceptable lipophilicity and bioavailability scores (~0.55), indicating a balanced hydrophilic–lipophilic profile that may support membrane permeability and interaction with biological targets. The log Kp values (~−6.51 cm/s) for both compounds suggest low skin permeability, which is typical for moderately polar molecules and does not negatively impact their potential as orally active agents.

Importantly, neither analog 2 nor analog 3 showed PAINS or Brenk alerts, suggesting a low likelihood of nonspecific or promiscuous binding, which supports the reliability of their observed enzyme inhibition. The synthetic accessibility scores (~2.8–2.9) indicate that both compounds are relatively easy to synthesize, consistent with the experimental procedures.

However, both compounds exhibited one lead-likeness violation, indicating that they do not fully comply with all criteria for ideal lead compounds. This suggests that while they possess promising characteristics, further structural optimization may be required to fully align with drug-likeness guidelines.

From a biological perspective, the favorable balance of polarity and lipophilicity, along with the absence of structural alerts, may contribute to the effective interaction of these compounds with α-amylase and α-glucosidase enzymes. In particular, analog 3, which demonstrated superior inhibitory activity, maintains a similar ADMET profile to analog 2, suggesting that its enhanced activity is more likely driven by molecular interaction efficiency (as supported by docking and DFT analysis) rather than major differences in pharmacokinetic properties.

Overall, the ADMET results indicate that both analog 2 and analog 3 fall within acceptable screening ranges for drug-like molecules, while also highlighting minor limitations such as lead-likeness deviation. These findings support their potential as promising candidates for further optimization, although experimental ADMET studies are required to confirm their pharmacokinetic and safety profiles.

## 3. Materials and Method

### 3.1. General Information

We sourced all necessary chemicals and reagents for synthesizing the pyrimidine-oxadiazole derivatives exclusively from Sigma-Aldrich (St. Louis, MO, USA). Structural elucidation of the synthesized derivatives was performed using NMR spectroscopy.^1^H NMR spectra were recorded at 600 MHz and ^13^C NMR spectra at 150 MHz using a Bruker AM spectrometer (Bruker, Berlin, Germany). NMR signals displayed characteristic splitting patterns: s (singlet), d (doublet), dd (doublet of doublets), t (triplet), dt (doublet of triplets), q (quartet), quint (quintet), sext (sextet), and m (multiplet). Corresponding coupling constants (*J*, Hz) are reported alongside. We acquired HR-EI-MS data using a Finnigan MAT-311A instrument (Finnigan, Bremen, Germany). Thin-layer chromatography (TLC) on pre-coated silica gel 60 F254 plates (Merck, Darmstadt, Germany) monitored reaction advancement and product purity, TLC spots were visualized under UV light at 254 nm and 365 nm wavelengths. Melting points were determined using a Büchi M-560 apparatus (BÜCHI Labortechnik AG, Flawil, Switzerland).

### 3.2. Methodology

#### 3.2.1. Synthesis of Ethyl 2-((4-Chloro-6-methylpyrimidin-2-yl)amino)acetate (II)

A mixture of 4-chloro-6-methylpyrimidin-2-amine (I) (1 mmol), ethyl 2-bromoacetate (1.2 mmol), and triethylamine (1.5 mmol) in ethanol was refluxed for 3 h. The reaction mixture was cooled to room temperature, and the solvent was evaporated under reduced pressure. The residue was poured into cold water, and the precipitate formed was filtered, washed, and dried to afford intermediate II.

#### 3.2.2. Synthesis of 2-((4-Chloro-6-methylpyrimidin-2-yl)amino)acetohydrazide (III)

Intermediate II (1 mmol) was dissolved in ethanol, and hydrazine hydrate (2 mmol) was added dropwise. The reaction mixture was refluxed for 4 h. After completion (monitored by TLC), the mixture was cooled, and the resulting solid was filtered, washed with cold ethanol, and dried to obtain intermediate III.

#### 3.2.3. Synthesis of Acylhydrazone Derivatives (IV)

Intermediate III (1 mmol) was reacted with substituted benzaldehydes (1 mmol) in ethanol in the presence of a catalytic amount of glacial acetic acid. The reaction mixture was refluxed for 3 h. Upon completion, the reaction mixture was cooled, and the resulting precipitate was filtered to yield the corresponding acylhydrazone intermediates (IV).

#### 3.2.4. Synthesis of Pyrimidine-Based 1,3,4-Oxadiazole Derivatives (1–10)

The acylhydrazone intermediate (IV) (1 mmol) was dissolved in 1,4-dioxane, followed by the addition of iodine (I_2_, catalytic amount) and potassium carbonate (K_2_CO_3_, 2 mmol). The reaction mixture was refluxed for 12 h. The progress of the reaction was monitored by TLC. The crude product was purified to afford the final pyrimidine–oxadiazole derivatives (1–10).

### 3.3. Alpha-Amylase Inhibition Assay Procedure

Porcine pancreatic α-amylase (Type VI-B, ≥10 U/mg protein, Sigma-Aldrich, USA) was used as the enzyme source. The enzyme solution was prepared in 20 mM phosphate buffer (pH 6.9) containing 6 mM NaCl. Soluble starch (1.0% *w*/*v*) was used as the substrate. In brief, 50 µL of test compounds at various concentrations were incubated with 50 µL of α-amylase solution for 10 min at 37 °C. Subsequently, 50 µL of 1.0% starch solution was added, and the reaction mixture was further incubated for 15 min. The reaction was terminated using 100 µL of 3,5-dinitrosalicylic acid (DNS) reagent, followed by heating at 95 °C for 5 min. Absorbance was measured at 540 nm.

### 3.4. Alpha-Glucosidase Inhibition Assay Procedure

α-Glucosidase from *Saccharomyces cerevisiae* (≥10 U/mg protein, Sigma-Aldrich, USA) was used as the enzyme source. The enzyme was prepared in 100 mM phosphate buffer (pH 6.8). The substrate p-nitrophenyl-α-D-glucopyranoside (pNPG) was used at a concentration of 5 mM. Briefly, 50 µL of test compounds were incubated with 50 µL of α-glucosidase solution for 10 min at 37 °C. Thereafter, 50 µL of 5 mM pNPG was added and incubated for 20 min. The reaction was terminated by adding 100 µL of 0.1 M Na_2_CO_3_, and absorbance was recorded at 405 nm.

#### Replicates and Statistical Analysis

All experiments were performed in triplicate (n = 3 independent experiments), and results are expressed as mean ± standard error of mean (SEM). IC_50_ values were calculated using nonlinear regression analysis. Statistical significance was determined using one-way ANOVA followed by appropriate post hoc tests, with *p* < 0.05 considered statistically significant. GraphPad Prism (version 8.0) was used for all statistical analyses.

### 3.5. Molecular Docking Study’s Test Methodology

A protein data bank (PDB) was used as a medium for retrieval of the crystalline structure, optimizing the structure by the removal of water molecules, co-factors and hetero-atoms and computing hydrogen bonds, charges and the missing atoms. Potent derivatives used for docking studies were prepared and then optimized by the use of a built and Ligand Preparation module implemented in Discovery Studio 2018 (Dassault Systemes BIOVIA, San Diego, CA, USA). A gold-docking tool was used for docking analysis. Ligand preparation involves generating varied tautomers, bond order assignments and stereochemistry. Furthermore, the amylase active site was surrounded by the receptor grid choosing the centroid of the complex ligand. A radius of 12 Å around the binding site was defined as an enzyme active site. Accomplishment of docking calculations was achieved using Chem PLP’s scoring function [43].

### 3.6. DFT Study Assay

The geometric parameters and energies were computed by density functional theory at the B3LYP/CEP-631G level of theory, using the GAUSSIAN 98W package of the programs [44], on geometries that were optimized at CEP-631G basis set. The high basis set was chosen to detect the energies at a highly accurate level. The atomic charges were computed using the natural atomic orbital populations. B3LYP is the key word for the hybrid functional [45], which is a linear combination of the gradient functionals proposed by Becke [46] and Lee, Yang and Parr [47], together with the Hartree–Fock local exchange function [48].

### 3.7. In Vivo Experiment

All in vivo experiments were conducted in accordance with internationally accepted guidelines for the care and use of laboratory animals and were approved by the Institutional Animal Ethics Committee (IAEC) (Approval No. AUST/Pharmacy/2026/11, approved on 6 January 2026).

## 4. Conclusions

In this study, a novel series of pyrimidine–oxadiazole derivatives was rationally designed, synthesized, and evaluated as dual inhibitors of α-amylase and α-glucosidase. Among the synthesized compounds, analogs 2, 3, and 6 exhibited the most potent inhibitory activity, surpassing the standard drug acarbose. Structure–activity relationship analysis indicated that the nature and position of substituents on the aromatic ring significantly influence enzyme inhibition, providing a basis for further structural refinement. Molecular docking studies supported these findings by demonstrating favorable binding interactions of the active compounds within the enzyme active sites. Preliminary in vivo safety evaluation in male Wistar rats revealed that the lead compound was well tolerated, with no significant toxicity observed in behavioral, hematological, or biochemical parameters. To further advance these compounds, future studies should focus on comprehensive pharmacokinetic evaluation, assessment of oral bioavailability, and validation of antidiabetic efficacy in disease-relevant animal models. Such investigations will be essential to establish their therapeutic potential and support progression toward preclinical development.

## Figures and Tables

**Table 1:** Spectral data of synthesized pyrimidine-based oxadiazole derivatives (1–10).

Compound	Molecular Formula	Structure	Yield (%)	M.p. (°C)	HR-EI-MS (m/z) [M]^+^ (Calcd./Found)	^1^H NMR (600 MHz, DMSO-d6) δ (ppm)	^13^C NMR (150 MHz, DMSO-d6) δ (ppm)
1	C_14_H_10_ClFN_6_O_4_	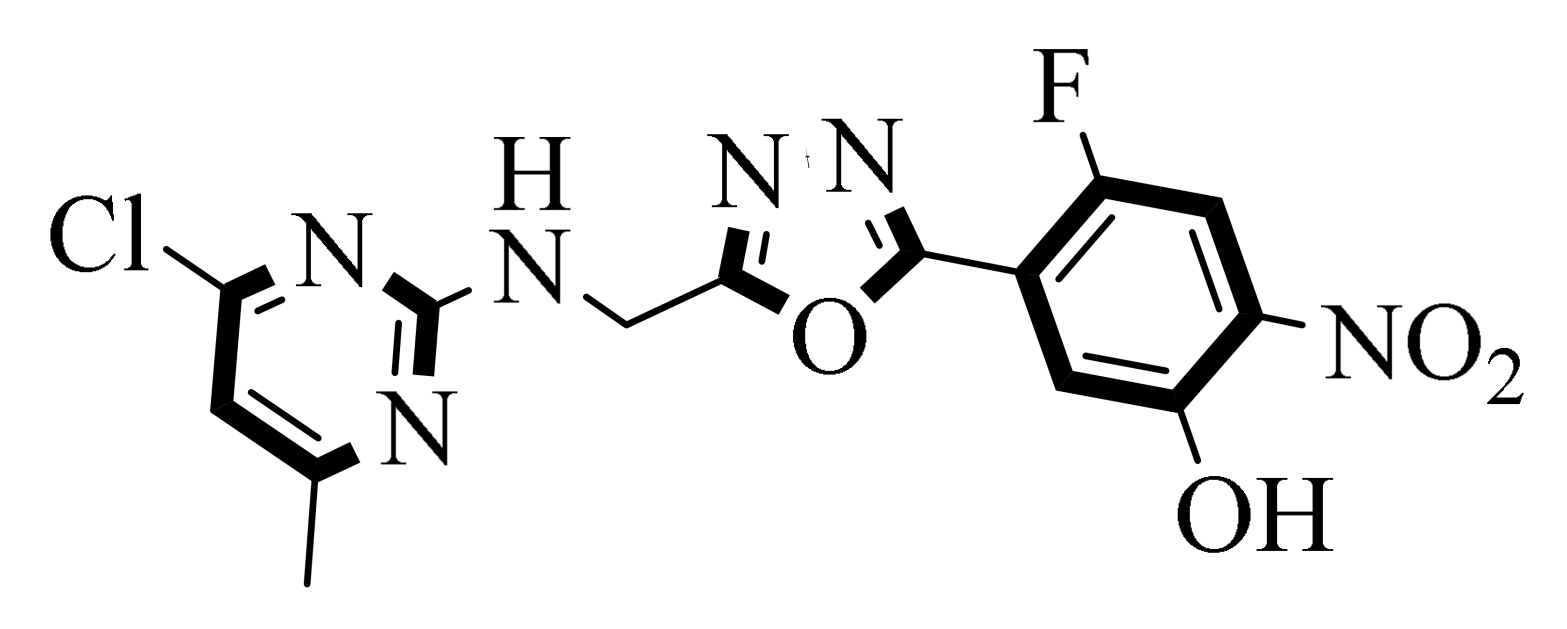	66	182–184	380.72/380.68	10.49 (s, NH), 9.30 (s, OH), 7.72 (s), 7.56 (s), 6.75 (s), 4.32 (s, 2H), 2.33 (s, 3H)	170.9, 164.4, 163.3, 161.1, 160.1, 150.3, 148.7, 136.5, 130.3, 115.9, 112.7, 103.6, 52.0, 23.5
2	C_14_H_10_ClF_2_N_5_O_2_	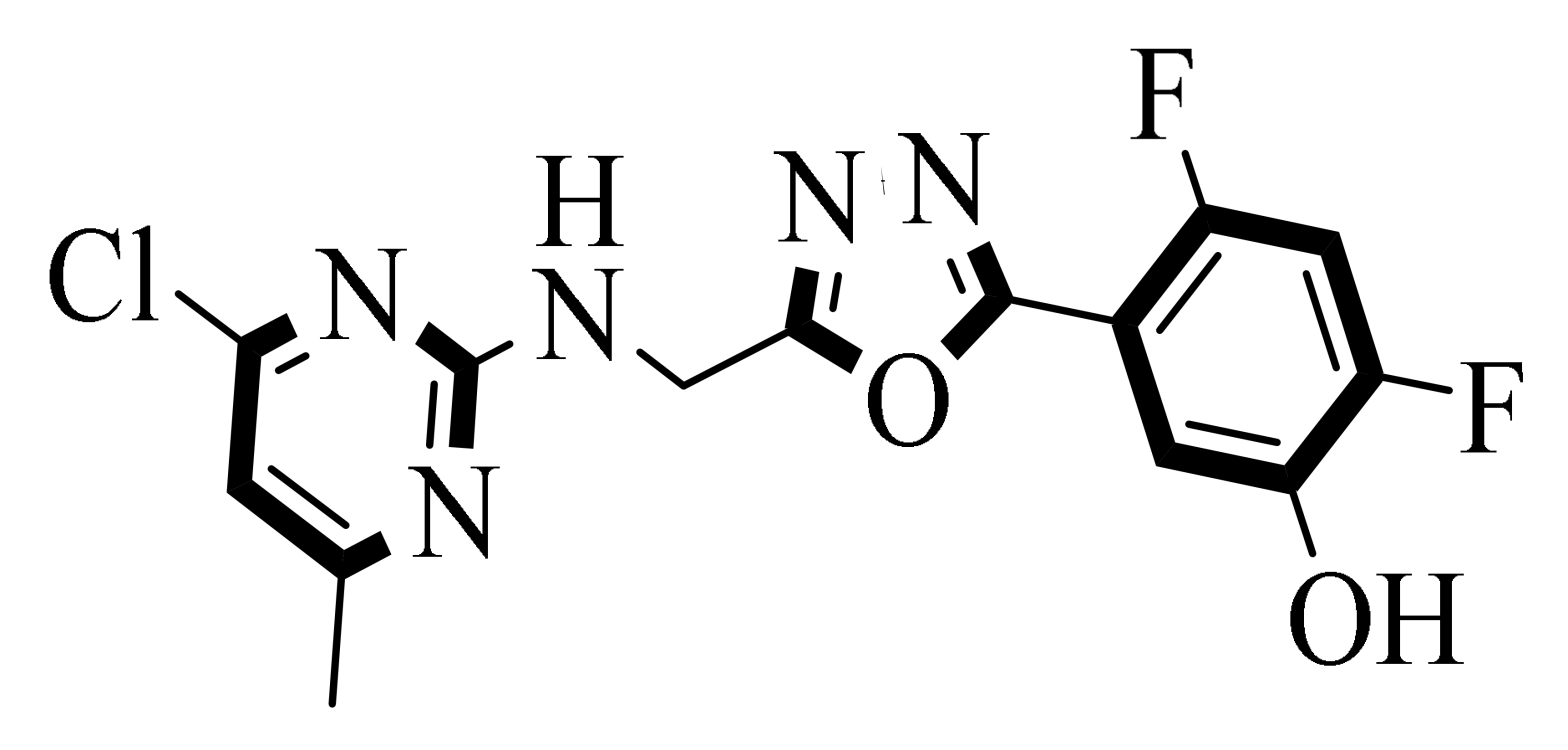	68	185–187	353.71/353.67	10.53 (s, NH), 9.74 (s, OH), 7.26 (s), 6.88 (s), 6.65 (s), 4.31 (s, 2H), 2.22 (s, 3H)	170.3, 164.4, 163.4, 161.7, 160.8, 155.3, 152.1, 142.2, 120.6, 116.8, 106.3, 103.2, 51.0, 23.4
3	C_14_H_10_ClF_2_N_5_O_2_	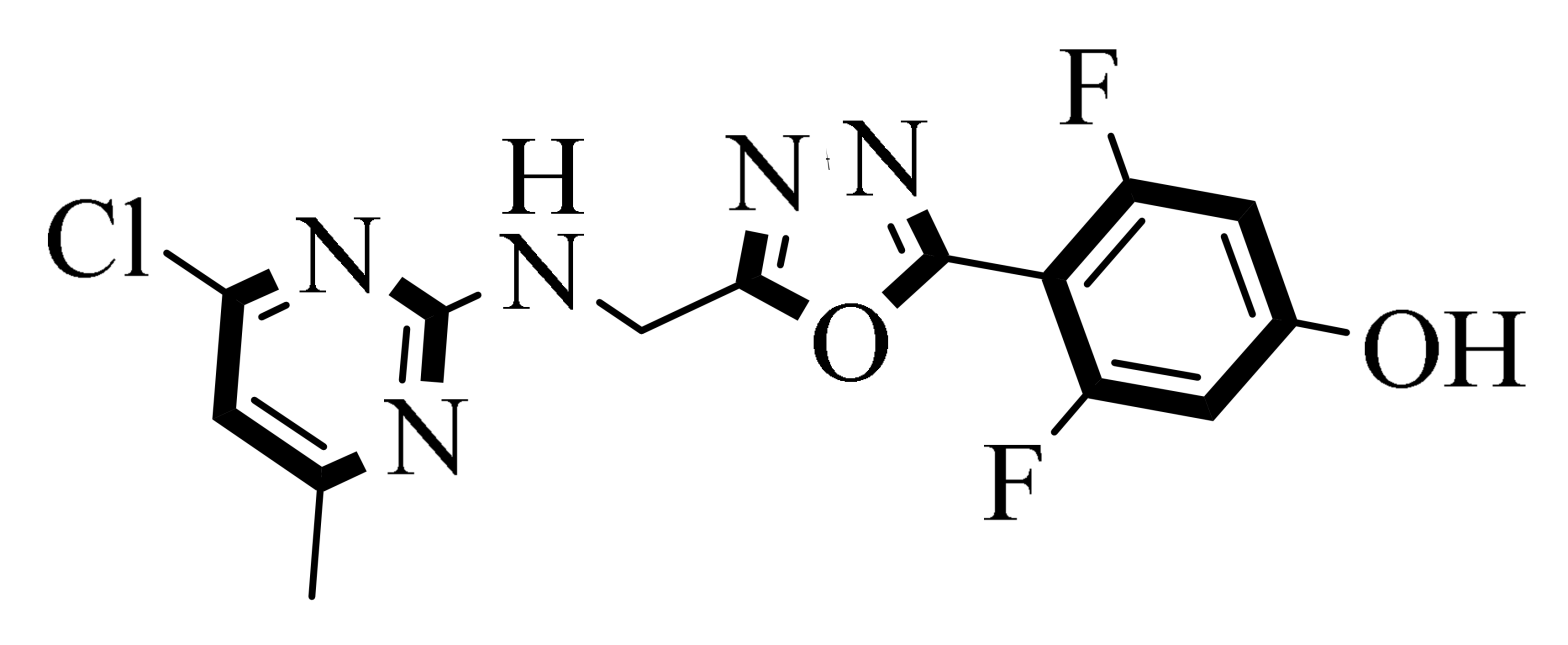	70	188–190	353.71/353.67	10.39 (s, NH), 9.36 (s, OH), 6.96 (s, 2H), 6.70 (s), 4.34 (s, 2H), 2.25 (s, 3H)	170.4, 165.7, 165.5, 164.9, 162.0, 161.9, 161.4, 160.3, 110.7, 103.0, 100.7, 50.6, 23.7
4	C_15_H_14_ClN_5_O	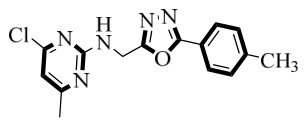	72	191–193	315.76/315.72	9.32 (s, NH), 8.72 (s), 7.51 (d), 7.08 (d), 6.06 (s), 4.13 (s, 2H), 3.82 (s, 6H)	170.2, 164.9, 151.7, 150.5, 145.6, 142.8, 137.5, 135.4, 134.4, 132.9, 132.0, 104.6, 51.3, 30.5, 25.0
5	C_14_H_11_ClN_6_O_3_	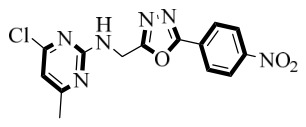	74	194–196	344.73/344.69	9.31 (s, NH), 7.58 (d), 7.03 (d), 6.76 (s), 4.17 (s, 2H), 3.80 (s, 3H)	171.3, 164.9, 151.4, 150.5, 147.5, 145.9, 144.5, 142.8, 137.4, 135.2, 100.4, 51.2, 33.4
6	C_14_H_12_ClN_5_O_3_	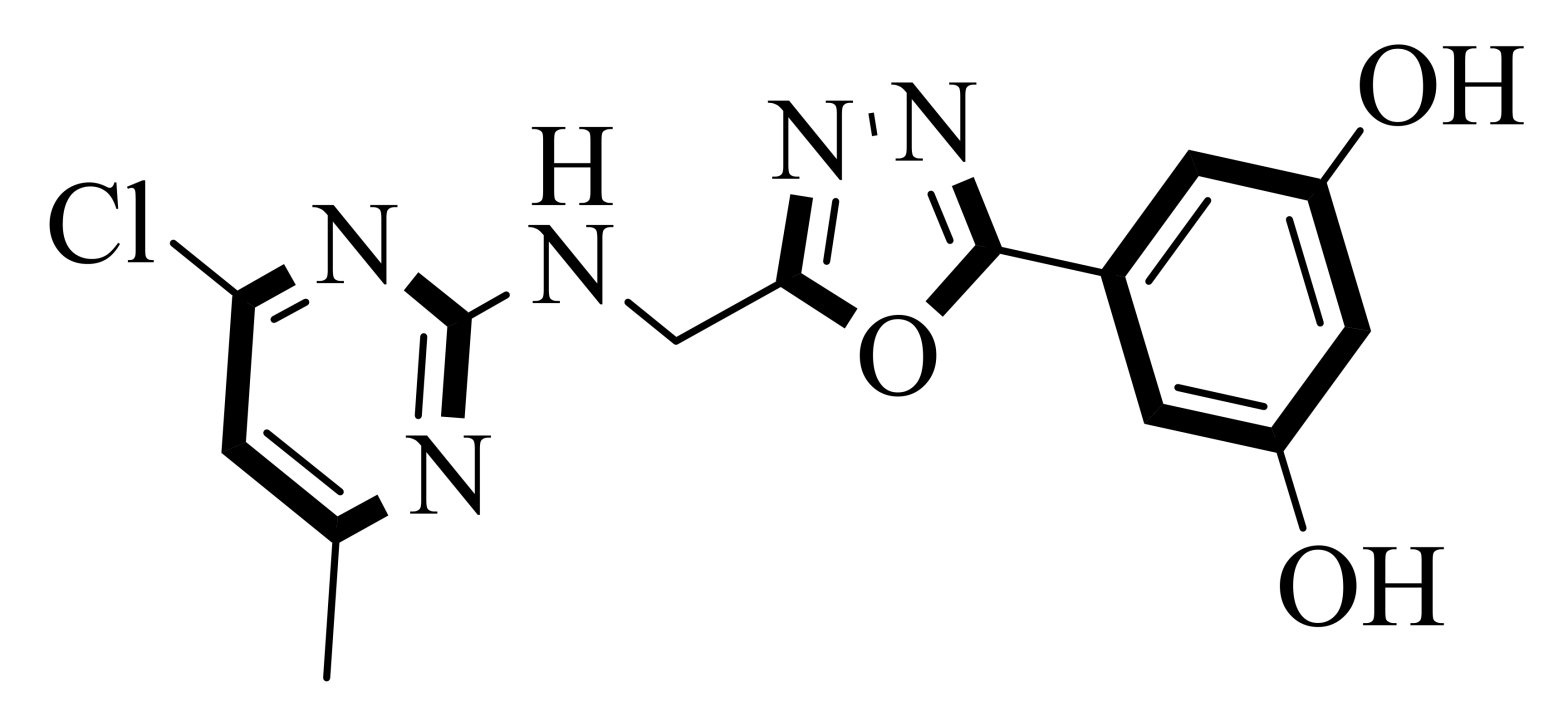	75	197–199	333.73/333.73	10.57 (s, NH), 9.37 (s, OH), 6.87 (s), 6.73 (s), 6.66 (s), 4.34 (s, 2H), 2.22 (s, 3H)	170.6, 165.8, 162.8, 161.9, 160.0, 158.7, 126.6, 105.4, 103.0, 52.6, 23.6
7	C_14_H_11_BrClN_5_O	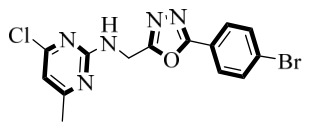	77	200–202	380.63/380.59	8.78 (s, NH), 8.35 (d), 7.93 (d), 4.39 (s, 2H), 3.82 (s, 3H)	172.3, 163.5, 160.4, 153.5, 150.5, 145.9, 142.6, 136.6, 133.4, 132.7, 132.3, 129.6, 55.4, 32.3
8	C_14_H_11_ClFN_5_O	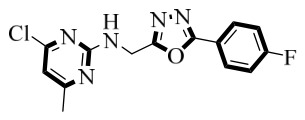	78	203–205	319.72/319.69	8.85 (s, NH), 7.85 (d), 7.56 (d), 6.76 (s), 4.24 (s, 2H), 3.85 (s, 3H)	172.4, 163.5, 160.4, 153.5, 150.1, 145.9, 142.0, 136.5, 133.6, 132.7, 55.5, 32.3
9	C_14_H_10_BrCl_2_N_5_O	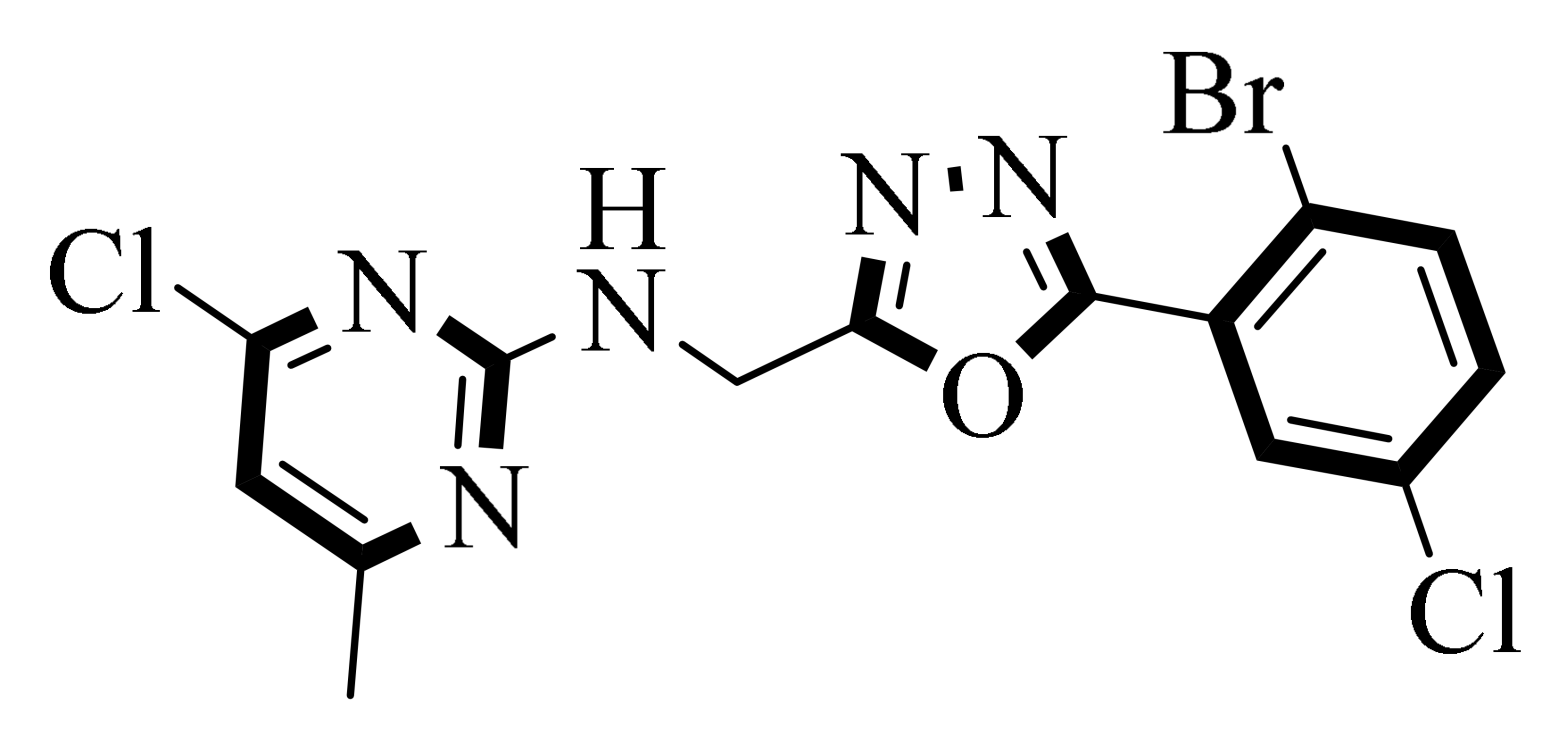	80	206–208	415.07/415.03	10.57 (s, NH), 7.70 (s), 7.53 (d), 7.31 (d), 6.68 (s), 4.34 (s, 2H), 2.20 (s, 3H)	171.3, 164.9, 151.4, 150.5, 147.5, 145.9, 144.5, 142.8, 137.4, 135.2, 102.4, 51.2, 33.4
10	C_15_H_11_ClN_6_O	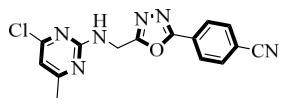	81	209–211	353.74/353.70	10.48 (s, NH), 7.89 (d), 7.42 (d), 6.65 (s), 4.24 (s, 2H), 2.21 (s, 3H)	170.2, 164.9, 151.7, 150.5, 145.6, 142.8, 137.0, 135.4, 132.5, 132.6, 119.7, 111.3, 104.8, 51.3, 25.0

**Table 2:** Various substituent, inhibitory potentials of novel pyrimidine-based oxadiazole derivatives (1–10) against α-amylase and α-glucosidase.

S/No	Compounds	IC_50_ = α-Amylase µM ± SEM	IC_50_ = α-Glucosidase µM ± SEM
1	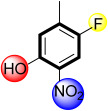	18.80 ± 0.10	19.10 ± 0.20
2	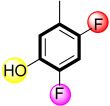	7.40 ± 0.30	8.10 ± 0.10
3	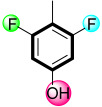	6.70 ± 0.20	7.10 ± 0.20
4	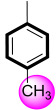	10.80 ± 0.10	11.10 ± 0.50
5	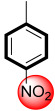	15.10 ± 0.20	15.50 ± 0.30
6	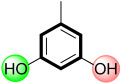	8.20 ± 0.10	8.80 ± 0.50
7	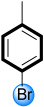	16.30 ± 0.20	17.10 ± 0.60
8	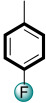	10.50 ± 0.10	10.90 ± 0.20
9	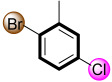	19.60 ± 0.20	20.10 ± 0.30
10	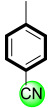	21.10 ± 0.10	21.80 ± 0.40
Standard drug Acarbose		10.10 ± 0.20	10.50 ± 0.10

All IC_50_ values are expressed in micromolar (µM) and are presented as mean ± standard error of the mean (SEM) from three independent experiments (*n* = 3).

**Table 3:** General in vivo parameters used for cytotoxicity assessment of compound **3**.

Parameter	Test Group	Observation
Route of administration	Intraperitoneal	As per protocol
Treatment duration	21 days	Uniform across groups
Daily dose	2.7 mg/kg	Constant dose
Survival	100%	No mortality
Behavioral response	Normal	No distress observed
Food consumption	Unchanged	Comparable to controls
Grooming and mobility	Normal	No impairment
Body weight trend	Physiological	No abnormal fluctuation
Clinical toxicity signs	None	Well tolerated
Serum biochemistry	Within normal limits	No organ dysfunction
Tissue morphology	Preserved	No pathological lesions

**Table 13:** The kinetic parameters including k_m_, V_max_ and k_i_ of analog 2 and 3.

Compound 3				
Parameter	Control (0.00 μM)	0.25 μM	0.50 μM	0.75 μM
Km	5.53	5.78	6.12	6.48
Vmax	0.432	0.432	0.432	0.432
Ki	—	1.76	1.76	1.76
**Compound 2**				
**Parameter**	**Control (0.00 μM)**	**0.25 μM**	**0.50 μM**	**0.75 μM**
Km	6.65	6.32	6.05	5.78
Vmax	0.457	0.435	0.412	0.390
Ki	—	2.24	2.24	2.24

**Table 14:** Binding interactions of potent compound **2** and **3** in comparison to standard drug acarbose.

Active Analog	Receptor	Type of Interaction	Distance (Å)	Docking Score (kcal/mol)
Compound **2** in α-Amylase complex	MET470	Pi-Alkyl	6.40	−9.92
	PHE601	Pi-Pi T-Shaped	6.34	
	ASP469	Halogen	4.88	
	ASP469	Pi-anion	6.63	
	TRP432	Pi-Pi T-Shaped	6.17	
	TRP432	Pi-Pi T-Shaped	7.01	
	TYR243	Alkyl	6.20	
	ALA234	Pi-Alkyl	5.19	
	ALA234	Pi-Alkyl	3.91	
	TRP329	Pi-Lone Pair	3.54	
Compound **2** in α-Glucosidase complex	GLN63	H Bond	5.68	−8.51
	THR163	H Bond	4.46	
	LEU165	Pi-Alkyl	5.59	
	LEU165	Alkyl	5.73	
	TRP59	Pi-Pi T-Shaped	5.22	
	TRP59	Pi-Pi Stacked	4.61	
	TRP59	C-H Bond	5.28	
	ALA198	Pi-Alkyl	4.61	
	LEU162	Alkyl	5.29	
	HIS299	Alkyl	6.14	
	ASP197	Pi-Anion	7.96	
	HIS101	Pi-Pi Stacked	6.69	
	TYR62	Pi-Alkyl	4.38	
	TYR62	C-H Bond	4.95	
Compound **3** in α-Amylase complex	GLU233	H Bond	5.39	−12.72
	HIS299	H Bond	5.93	
	ASP300	Pi-anion	6.80	
	TRP59	Pi-Pi T-Shaped	6.21	
	TRP59	Pi-Alkyl	4.20	
	TRP59	Pi-Alkyl	6.18	
	HIS305	Pi-Alkyl	5.63	
	LEU561	Pi-Alkyl	5.61	
Compound **3** in α-Glucosidase complex	ALA628	Alkyl	7.27	−11.81
	ALA234	Pi-Alkyl	5.74	
	ALA602	Pi-Alkyl	7.27	
	TRP432	H Bond	5.47	
	TRP432	Pi-Alkyl	4.88	
	TRP329	Pi-Alkyl	6.62	
	PHE601	Pi-Pi Stacked	6.86	
	PHE601	Pi-Alkyl	5.25	
	ILE358	Pi-Alkyl	4.05	
	ILE396	Pi-Alkyl	6.17	
	ASP469	Sulfur-X	5.25	
	TRP467	Pi-Alkyl	6.65	
	HIS626	Pi-Alkyl	5.79	
Acarbose in α-Amylase complex	GLU240	H Bond	5.16	−7.21
	GLU240	H Bond	4.10	
	GLU240	C-H Bond	4.83	
	GLY306	H Bond	3.25	
	ILE235	C-H Bond	4.02	
	ILE235	C-H Bond	3.24	
	TYR62	H Bond	5.32	
Acarbose in α-Glucosidase complex	THR163	H Bond	4.19	−7.88
	THR163	H Bond	5.18	
	ALA106	H Bond	3.19	
	LEU165	Alkyl	5.67	
	ASP220	C-H Bond	4.12	
	ASP220	H Bond	4.20	
	GLU233	C-H Bond	5.88	
	ASP-468	Pi-anion	6.36	

**Table 15:** Docking validation results (redocking RMSD).

Protein (PDB ID)	Co-Crystallized Ligand	RMSD (Å)
α-Amylase	Acarbose	1.42
α-Glucosidase		1.67

## Data Availability

The datasets generated during and/or analyzed during the current study are available from the corresponding author upon reasonable request.

## Supplementary Materials

The following supporting information can be downloaded at: https://www.mdpi.com/article/10.3390/ph19071085/s1, Figure S1: Graph inhibition curve of analog 2 for α-amylase; Figure S2: Graph inhibition curve of analog 2 for α-glucosidas; Figure S3: Structural basis of analog-2 interaction with α-amylase at the receptor binding region; Figure S4: Structural basis of analog-2 interaction with α-glucosidase at the receptor binding region; Figure S5: The MEP profile and electronic configuration of analog 2 reveal distinct electrophilic and nucleophilic zone. Figure S6: FMO analysis of analog-2; Spectral analysis; Figure S7: Proton spectral analysis of compound-**1**; Figure S8: Carbon spectral analysis of compound-**1**; Figure S9: HR-mass spectral analysis of compound-**1**; Figure S10: Proton spectral analysis of compound-**2**; Figure S11: Carbon spectral analysis of compound-**2**; Figure S12: HR-mass spectral analysis of compound-**2**; Figure S13: Proton spectral analysis of compound-**3**; Figure S14: Carbon spectral analysis of compound-**3**; Figure S15: HR-mass spectral analysis of compound-**3**; Figure S16: Proton spectral analysis of compound-**4**; Figure S17: Carbon spectral analysis of compound-**4**; Figure S18: HR-mass spectral analysis of compound-**4**; Figure S19: Proton spectral analysis of compound-**5**; Figure S20: Carbon spectral analysis of compound-**5**; Figure S21: HR-mass spectral analysis of compound-**5**; Figure S22: Proton spectral analysis of compound-**6**; Figure S23: Carbon spectral analysis of compound-**6**; Figure S24: HR-mass spectral analysis of compound-**6**; Figure S25: Proton spectral analysis of compound-**7**; Figure S26: Carbon spectral analysis of compound-**7**; Figure S27: HR-mass spectral analysis of compound-**7**; Figure S28: Proton spectral analysis of compound-**8**; Figure S29: Carbon spectral analysis of compound-**8**; Figure S30: HR-mass spectral analysis of compound-**8**; Figure S31: Proton spectral analysis of compound-**9**; Figure S32: Carbon spectral analysis of compound-**9**; Figure S33: HR-mass spectral analysis of compound-**9**; Figure S34: Proton spectral analysis of compound-**10**; Figure S35: Carbon spectral analysis of compound-**10**; Figure S36: HR-mass spectral analysis of compound-**10**.
