# Supplementary material for: Medicinally Tuned Pyrimidine–Oxadiazole Hybrids: Synthetic Development, Enzyme-Targeted Evaluation, In Vivo Toxicological Assessment and Computational Investigations Against Diabetes Mellitus
Source: Pharmaceuticals (Basel). 2026 Jul 15;19(7):1085. doi: 10.3390/ph19071085 (PMC13414850; doi:10.3390/ph19071085)
Supplement: Supplementary file 1 [file pharmaceuticals-19-01085-s001.zip › pharmaceuticals-4254075-supplementary.pdf]

pharmaceuticals

MDPI

Supplementary Material

Medicinally Tuned Pyrimidine–Oxadiazole Hybrids: Synthetic Development, Enzyme-Targeted Evaluation, In Vivo Toxicological Assessment and Computational Investigations Against Diabetes Mellitus

Shifa Felemban and M.M. Khowdiary *

Department of Chemistry, Faculty of Applied Science, University College-Al Leith, University of Umm Al-Qura, Makkah 21955, Saudi Arabia

*Corresponding author: *Mmkhowdiary@uqu.edu.sa* (M.M. Khowdiary)

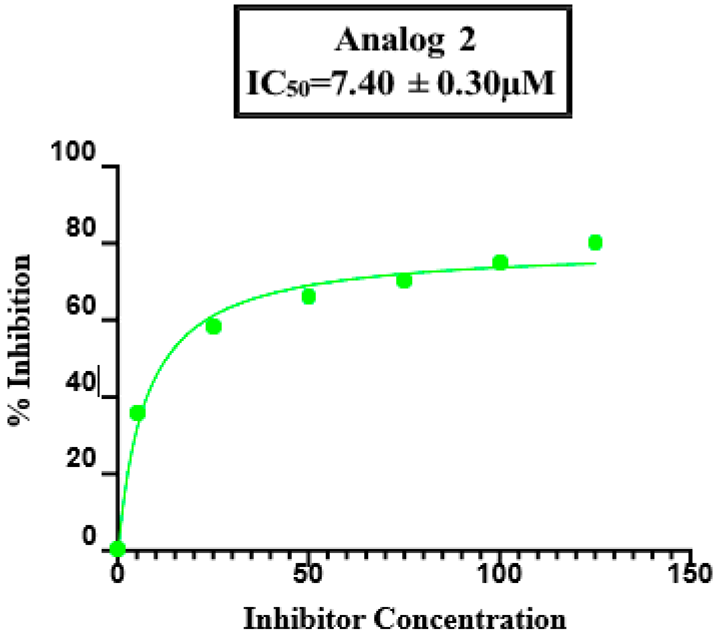

Figure S1. Graph inhibition curve of analog 2 for α-amylase.

 https://doi.org/10.3390/ph19071085

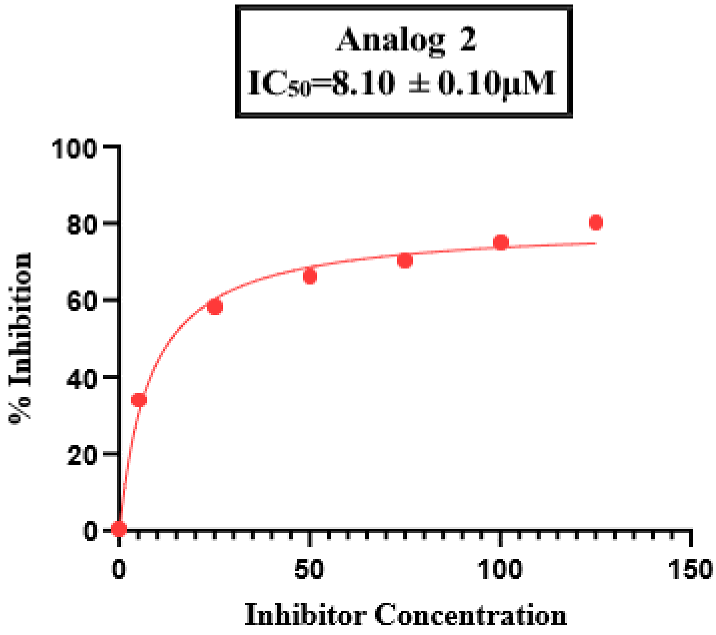

Figure S2. Graph inhibition curve of analog 2 for α-glucosidase.

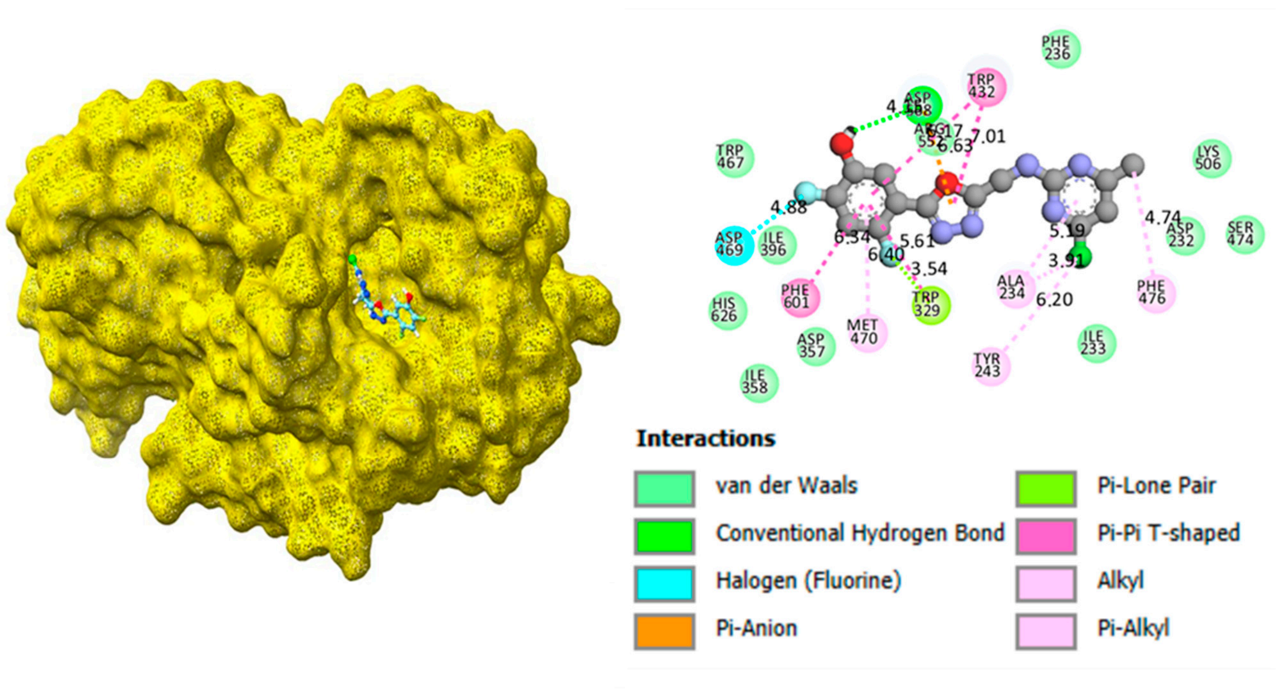

Figure S3. Structural basis of analog-2 interaction with α-amylase at the receptor binding region.

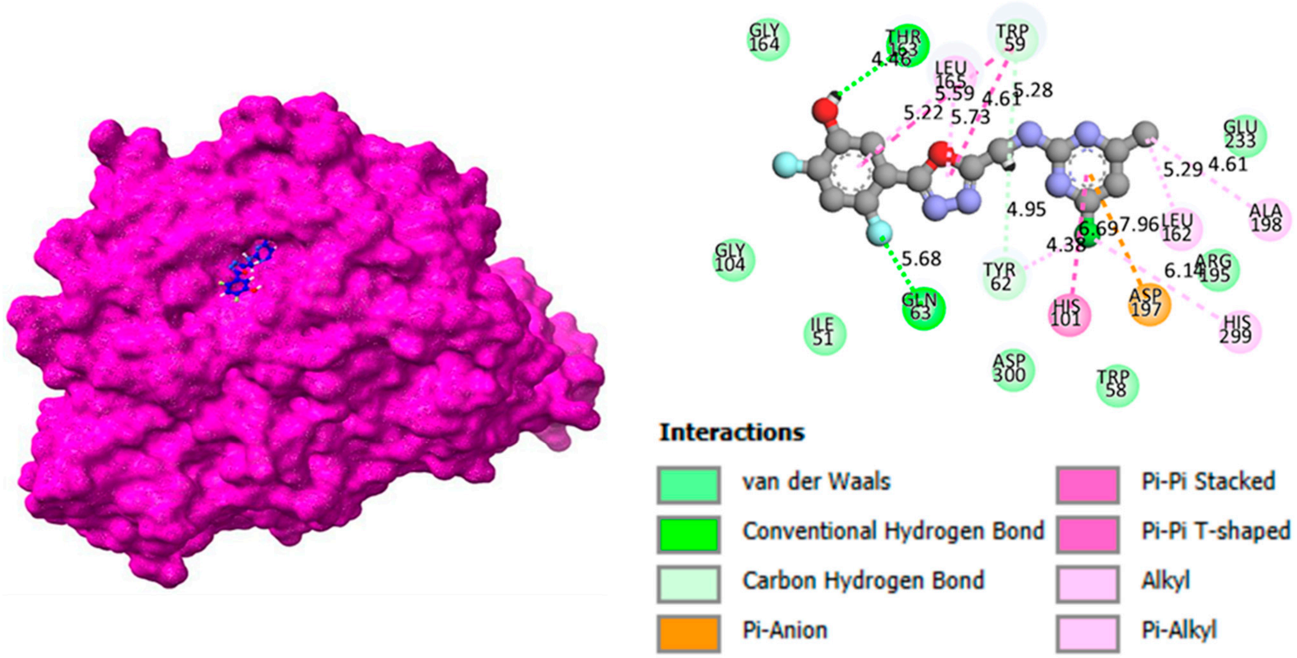

Figure S4. Structural basis of analog-2 interaction with α-glucosidase at the receptor binding region.

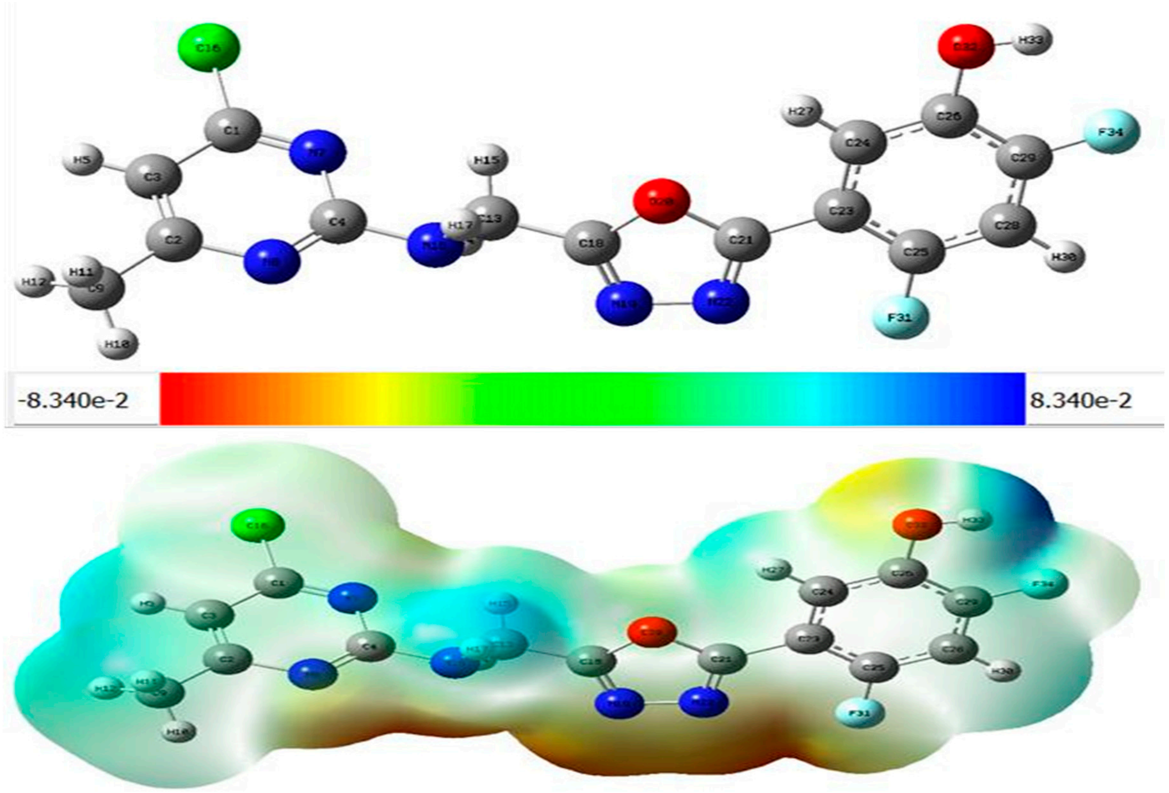

Figure S5. The MEP profile and electronic configuration of analog 2 reveal distinct electrophilic and nucleophilic zone.

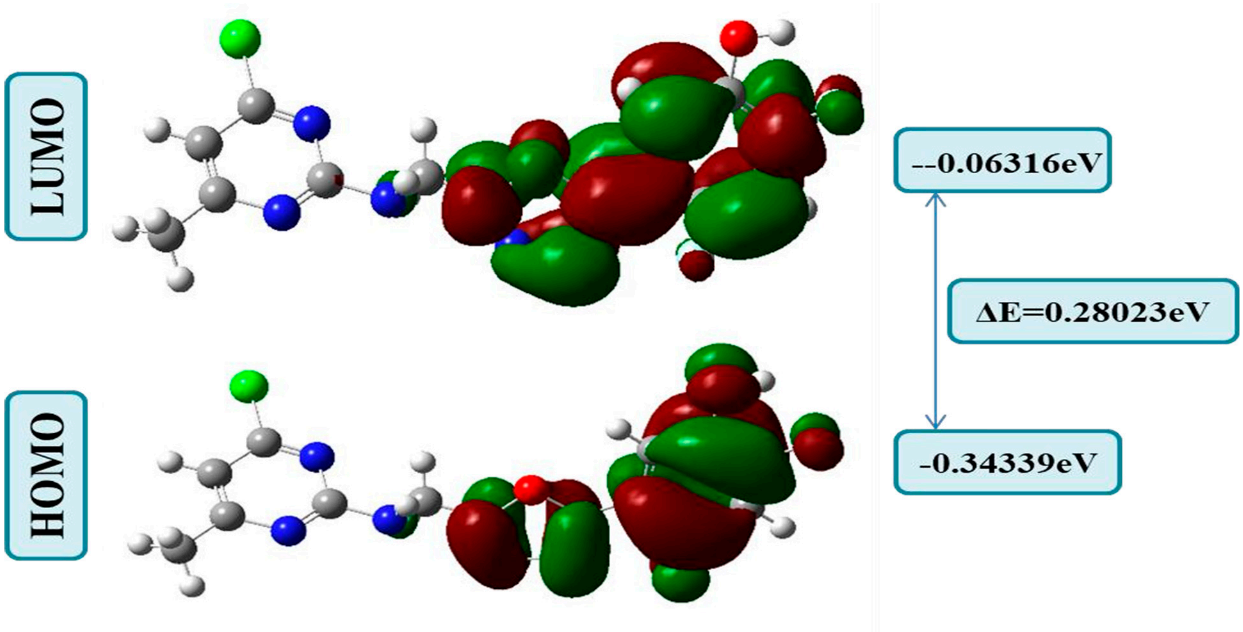

Figure S6. FMO analysis of analog-2.

S.1 Spectral Analysis:

1. 5-(5-(((4-chloro-6-methylpyrimidin-2-yl)amino)methyl)-1,3,4-oxadiazol-2-yl)-4-fluoro-2-nitrophenol

^{1}H NMR (600 MHz, DMSO-*d6*): δ 10.49 (s, 1H, H-NH), 9.30 (s, 1H, H-OH), 7.72 (s, 1H, Ar-H), 7.56 (s, 1H, Ar-H), 6.75 (s, 1H, H-pyrimidine), 4.32 (s, 2H, H-methylene), 2.33 (s, 3H, methyl); ^{13}C NMR (150 MHz, DMSO-*d6)*: δ 170.9, 164.4, 163.3, 161.1, 160.1,150.3, 148.7, 136.5, 130.3, 115.9, 112.7, 103.6, 52.0, 23.5; HR EIMS*: m/z* calcd for C14H10ClFN6O4 $[M]^+$ 380.72 Found 380.68.

1. 5-(5-(((4-chloro-6-methylpyrimidin-2-yl)amino)methyl)-1,3,4-oxadiazol-2-yl)-2,4-difluorophenol

$^{1.}$ H NMR (600 MHz, DMSO-d6): δ 10.53 (s, 1H, H-NH), 9.74 (s, 1H, H-OH), 7.26 (s, 1H, Ar-H), 6.88 (s, 1H, H-pyrimidine), 6.65 (s, 1H, Ar-H), 4.31 (s, 2H, H-methylene), 2.22 (s, 3H, H-methyl); ^{13}C NMR (150 MHz, DMSO-d6): δ 170.3, 164.4, 163.4, 161.7 160.8, 155.3, 152.1, 142.2, 120.6, 116.8, 106.3, 103.2, 51.0, 23.4 ; HR EIMS: m/z calcd for C14H10ClF2N5O2 $[M]^+$ 353.71 Found 353.67.

1. 4-(5-(((4-chloro-6-methylpyrimidin-2-yl)amino)methyl)-1,3,4-oxadiazol-2-yl)-3,5-difluorophenol

^{1}H NMR (600 MHz, DMSO-*d6*): δ 10.39 (s, 1H, H-NH), 9.36 (s, 1H, H-OH), 6.96 (s, 2H, Ar-H), 6.70 (s, 1H, H-pyrimidine), 4.34 (s, 2H, H-methylene), 2.25 (s, 3H, H- methyl); ^{13}CNMR (150 MHz, DMSO-*d6)*: δ 170.4, 165.7, 165.5, 164.9, 162.0, 161.9,161.4, 160.3, 110.7, 103.0, 100.7, 50.6, 23.7 ; HR EIMS*: m/z* calcd for C14H10ClF2N5O2 $[M]^+$ 353.71Found 353.67.

1. 4-chloro-6-methyl-N-((5-(p-tolyl)-1,3,4-oxadiazol-2-yl)methyl)pyrimidin-2-amine

^{1}H NMR (600 MHz, DMSO-*d6*): δ 9.32 (s, 1H, H-NH), 8.72(s, 1H, H-OH), 7.51 (d, *J* = 7.53 Hz, 2H, Ar-H), 7.08 (d, *J* = 7.30 Hz, 2H, Ar-H), 6.06 (s, 1H, H-pyrimidine), 4.13(s, 2H, H-methylene), 3.82(s, 6H, H-methyl); ^{13}C NMR (150 MHz, DMSO-*d6)*: δ 170.2, 164.9,

151.7, 150.5 145.6, 142.8, 137.5, 135.4, 134.4, 132.9, 132.0, 104.6, 51.3, 30.5, 25.0; HR EIMS*: m/z* calcd for C15H14ClN5O [M]$^+$ 315.76 Found 315.72

1. 4-chloro-6-methyl-N-((5-(4-nitrophenyl)-1,3,4-oxadiazol-2-yl)methyl)pyrimidin-2- amine

^{1}H NMR (600 MHz, DMSO-*d6*): *δ* 9.31 (s, 1H, H-NH), 7.58 (d, *J* = 7.65 Hz, 2H, Ar-H, 7.03 (d, *J* = 7.34 Hz, 2H, Ar-H), 6.76 (s, 1H, H-pyrimidine), 4.17 (s, 2H, H- methylene), 3.80 (s, 3H, H-methyl); ^{13}C NMR (150 MHz, DMSO-*d6)*: *δ* 171.3, 164.9,151.4, 150.5, 147.5, 145.9, 144.5, 142.8, 137.4, 135.2, 100.4, 51.2, 33.4; HR EIMS*: m/z* calcd for C14H11ClN6O3 [M]$^+$ 344.73 Found 344.69.

1. 5-(5-(((4-chloro-6-methylpyrimidin-2-yl)amino)methyl)-1,3,4-oxadiazol-2- yl)benzene-1,3-diol

^{1}H NMR (600 MHz, DMSO-*d6*): *δ* 10.57 (s, 1H, H-NH), 9.37 (s, 2H, H-OH), 6.87 (s, 2H, Ar-H),6.73 (s, 1H, H-pyrimidine), 6.66 (s, 1H, Ar-H), 4.34 (s, 2H, H- methylene), 2.22 (s, 3H, H-methyl); ^{13}C NMR (150 MHz, DMSO-*d6)*: *δ* 170.6, 165.8, 162.8, 161.9, 160.0, 158.7, 158.7, 126.6, 105.4, 105.4 ,103.0, 52.6, 23.6; HR EIMS*: m/z* calcd for C14H12ClN5O3 [M]$^+$ 333.73 Found 333.73.

1. N-((5-(4-bromophenyl)-1,3,4-oxadiazol-2-yl)methyl)-4-chloro-6-methylpyrimidin-2- amine

^{1}H NMR (600 MHz, DMSO-*d6*): *δ* 8.78 (s, 1H, H-NH), 8.35 (d, *J* = 7.20 Hz, 2H, Ar-H, 7.93 (d, *J* = 7.43 Hz, 2H, Ar-H), 4.39 (s, 2H, H-methylene), 3.82 (s, 3H, H- methyl) ; ^{13}C NMR (150 MHz,DMSO-*d6)*: *δ* 172.3, 163.5, 160.4, 153.5, 150.5,145.9, 142.6, 136.6, 133.4, 132.7, 132.3, 129.6, 55.4, 32.3; HR EIMS*: m/z* calcd for C14H11BrClN5O [M]$^+$ 380.63 Found 380.59.

4. 4-chloro-N-((5-(4-fluorophenyl)-1,3,4-oxadiazol-2-yl)methyl)-6-methylpyrimidin-2- amine

^{1}H NMR (600 MHz, DMSO-*d6*): *δ* 8.85 (s, 1H, H-NH), 7.85 (d, *J* = 7.76 Hz, 2H, Ar-H, 7.56 (d, *J* = 7.53 Hz, 2H, Ar-H), 6.76 (s, 1H, H-pyrimidine), 4.24 (s, 2H, H- methylene), 3.85 (s, 3H, H-methyl); ^{13}C NMR (150 MHz, DMSO-*d6)*: *δ* 172.4, 163.5, 160.4, 153.5, 150.1, 145.9, 142.0, 136.5, 133.6 132.7, 55.5, 32.3; HR EIMS*: m/z*, calcd for C14H11ClFN5O [M]$^+$ 319.72 Found 319.69.

1. N-((5-(2-bromo-5-chlorophenyl)-1,3,4-oxadiazol-2-yl)methyl)-4-chloro-6-methylpyrimidin-2-amine

^{1}H NMR (600 MHz, DMSO-*d6*): *δ* 10.57 (s, 1H, H-NH), 7.70 (s, 1H, Ar-H), 7.53 (d, *J* = 7.53 Hz, 1H, Ar-H),7.31 (d, *J* = 7.66 Hz, 1H, Ar-H) 6.68 (s, 1H, H-pyrimidine), 4.34 (s, 2H, H-methylene), 2.20 (s, 3H, H-methyl); ^{13}C NMR (150 MHz, DMSO-*d6)*: *δ* 171.3, 164.9, 151.4, 150.5, 147.5, 145.9, 144.5, 142.8, 137.4, 135.2, 102.4, 51.2, 33.4 ;HR EIMS*: m/z* calcd for C14H10BrCl2N5O [M]$^+$ 415.07 Found 415.03.

1. 4-(5-(((4-chloro-6-methylpyrimidin-2-yl)amino)methyl)-1,3,4-oxadiazol-2- yl)benzonitrile

^{1}H NMR (600 MHz, DMSO-*d6*):): *δ* 10.48 (s, 1H, H-NH), 7.89 (d, *J* = 7.72 Hz, 2H, Ar-H), 7.42 (d, *J* = 7.87 Hz, 2H, Ar-H), 6.65 (s, 1H, H-pyrimidine), 4.24 (s, 2H, H- methylene), 2.21 (s, 3H, H-methyl); ^{13}C NMR (150 MHz, DMSO-*d6)*: *δ* 170.2, 164.9,151.7, 150.5, 145.6, 142.8, 137.0, 135.4, 132.5, 132.6, 119.7 111.3, 104.8, 51.3, 25.0; HR EIMS*: m/z* calcd for C15H11ClN6O [M]$^+$ 353.74 Found 353.70.

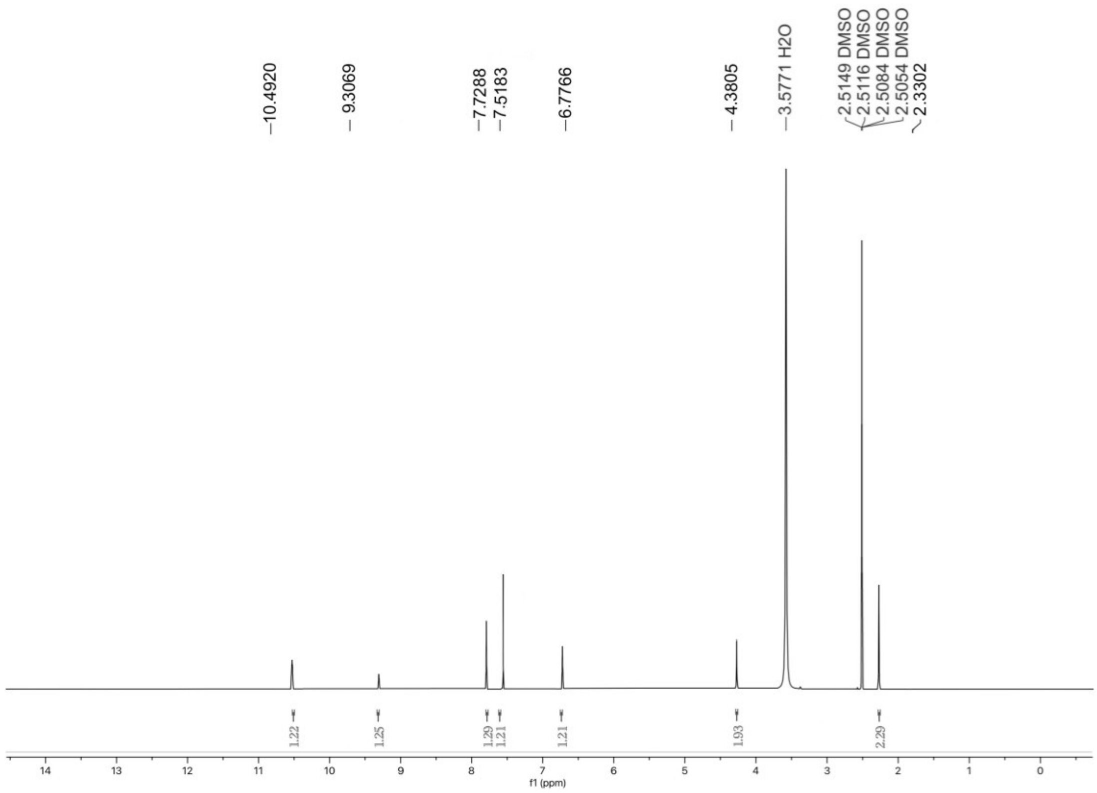

Figure S7. Proton spectral analysis of compound-1.

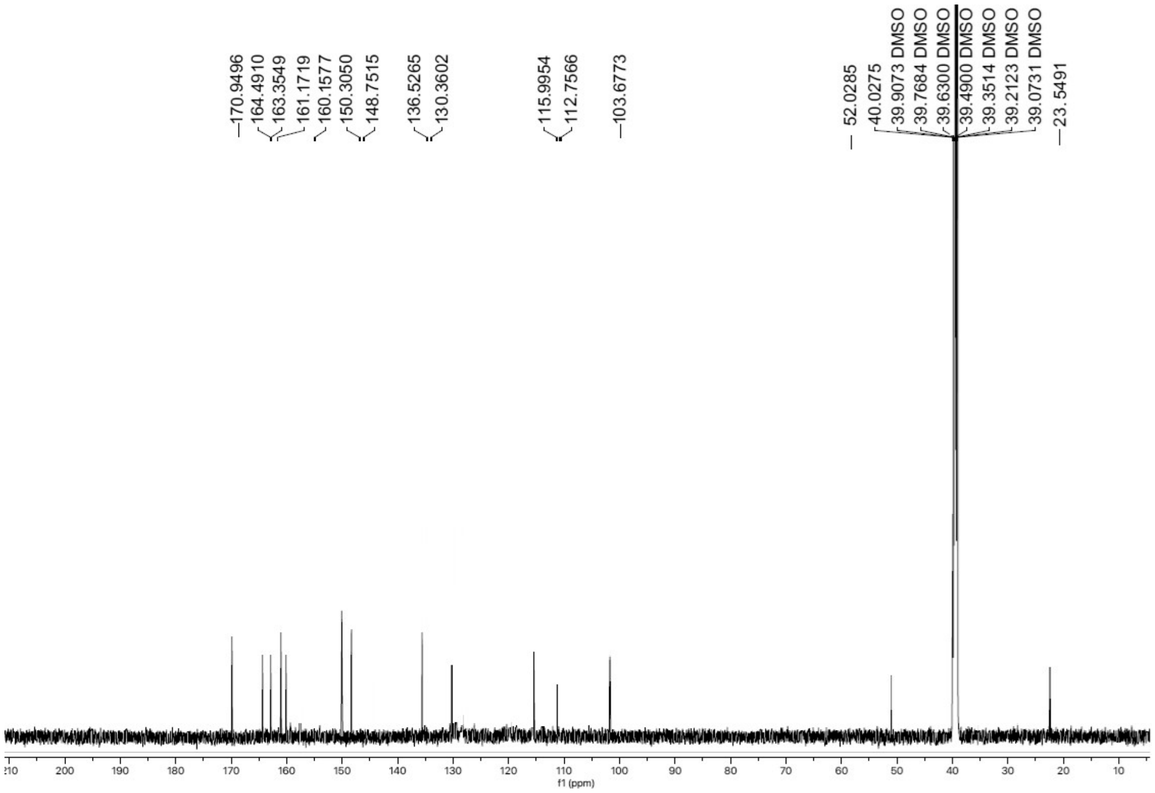

Figure S8. Carbon spectral analysis of compound-1.

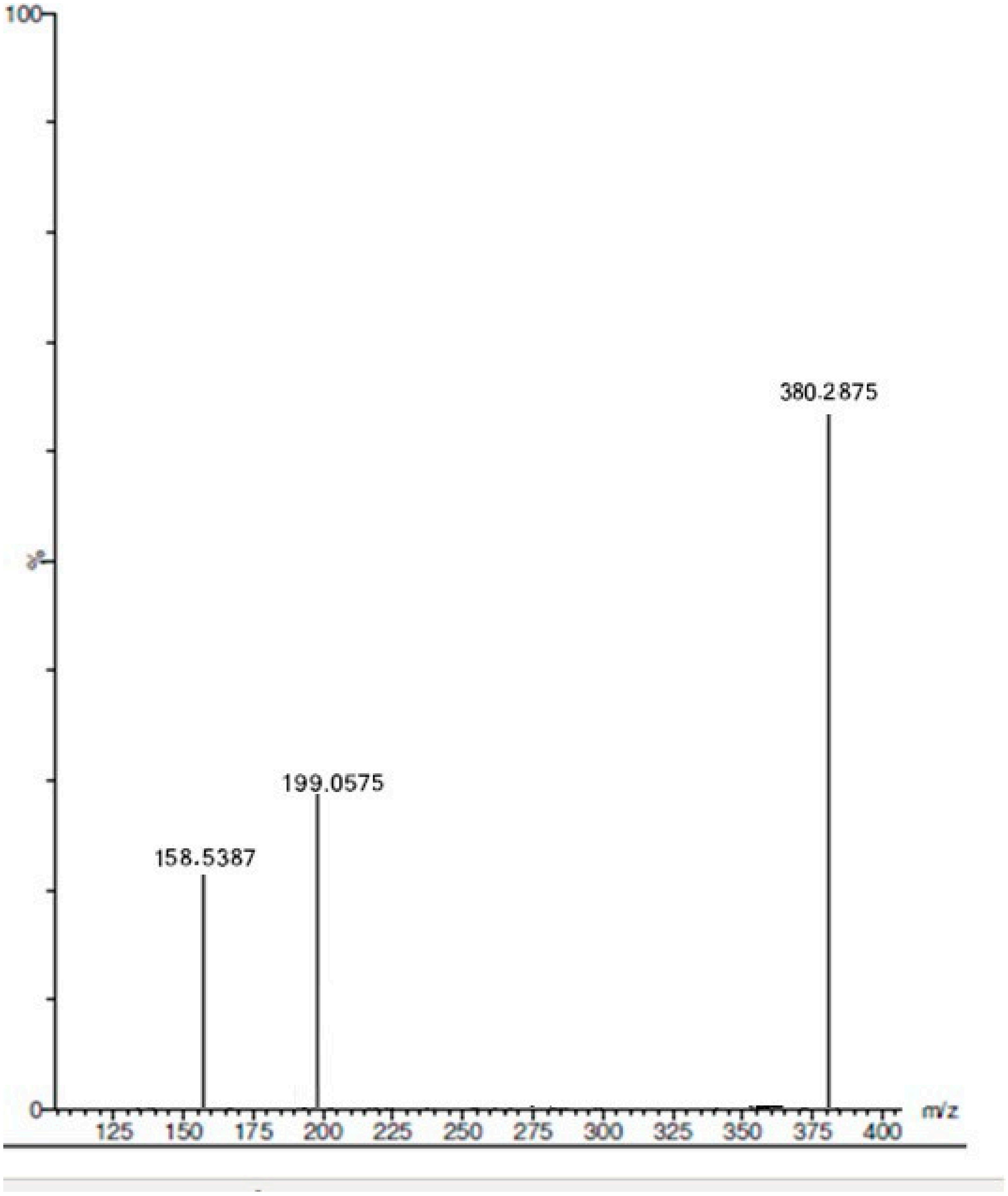

Figure S9. HR-mass spectral analysis of compound-1.

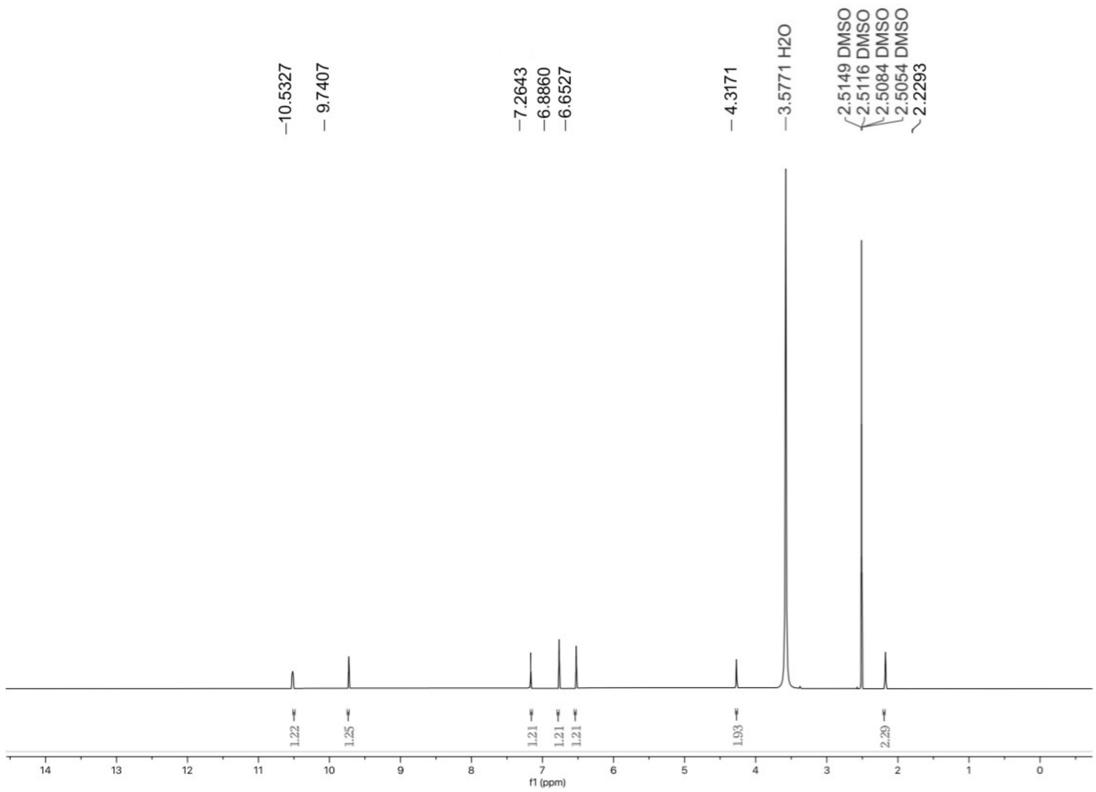

Figure S10. Proton spectral analysis of compound-2.

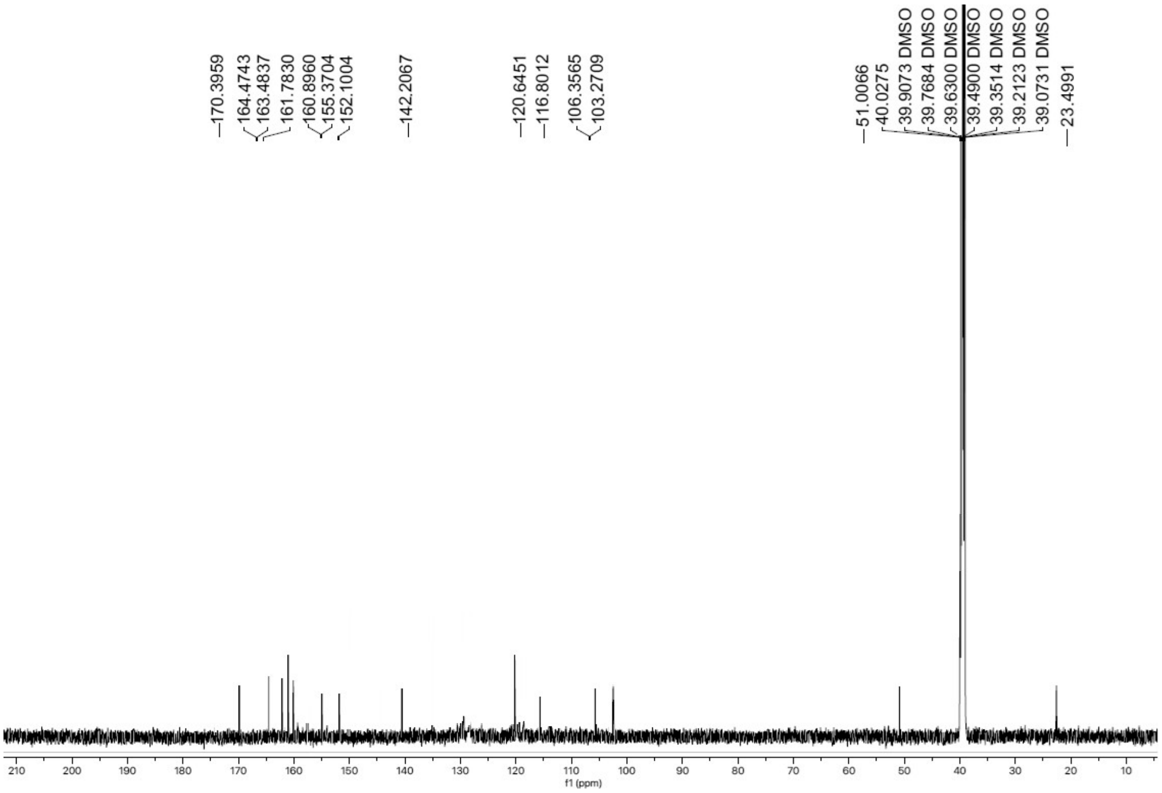

Figure S11. Carbon spectral analysis of compound-2.

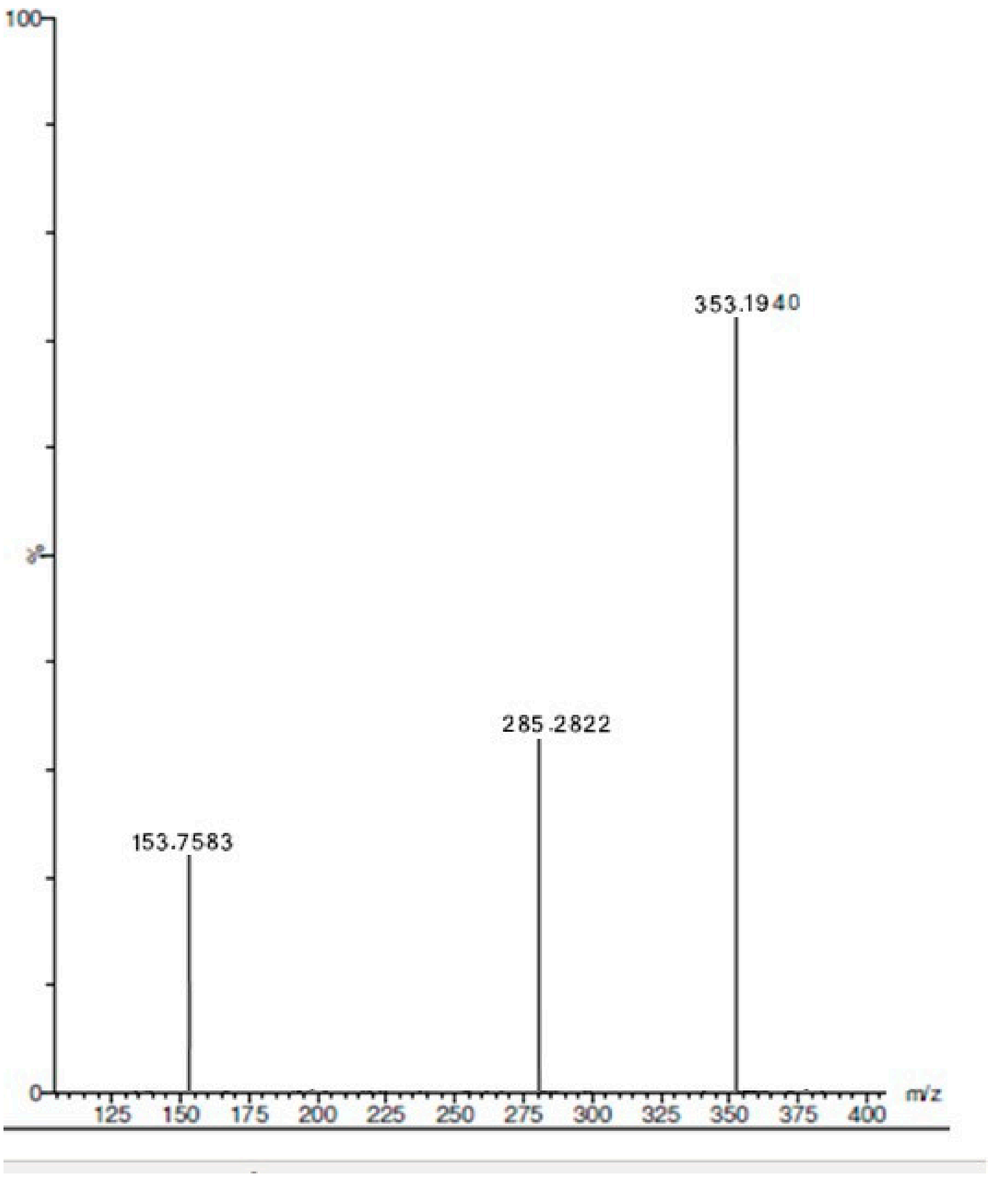

Figure S12. HR-mass spectral analysis of compound-2.

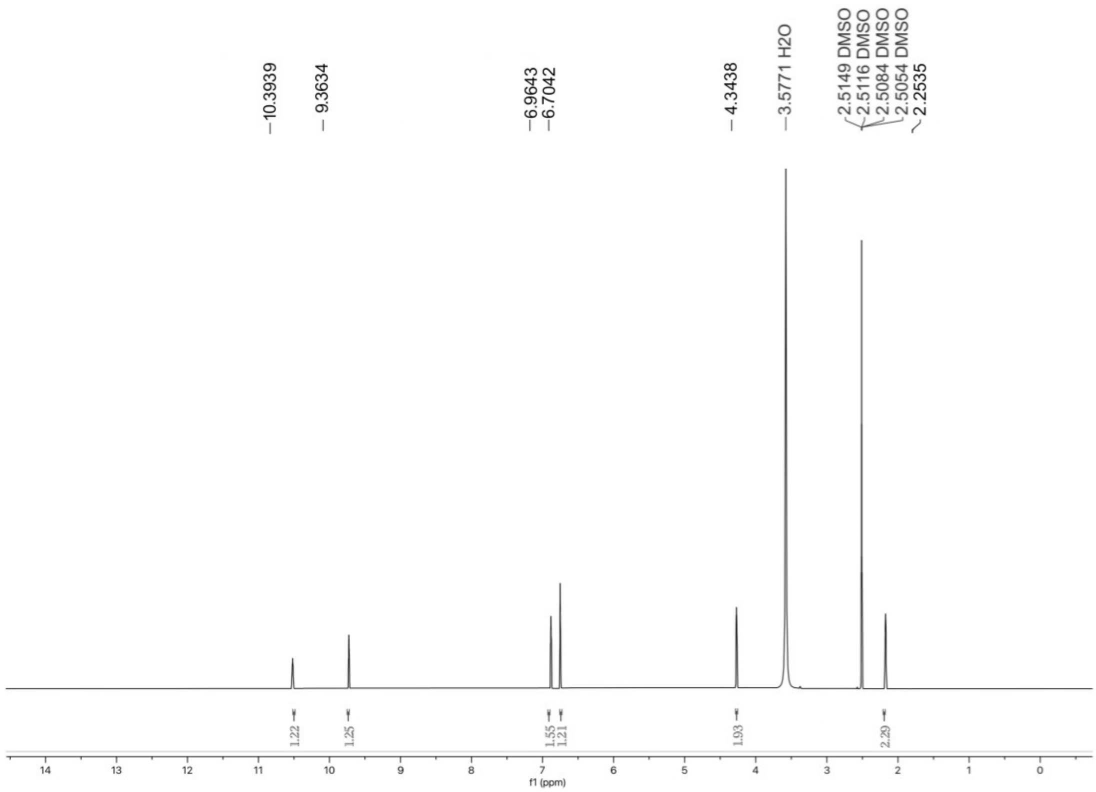

Figure S13. Proton spectral analysis of compound-3.

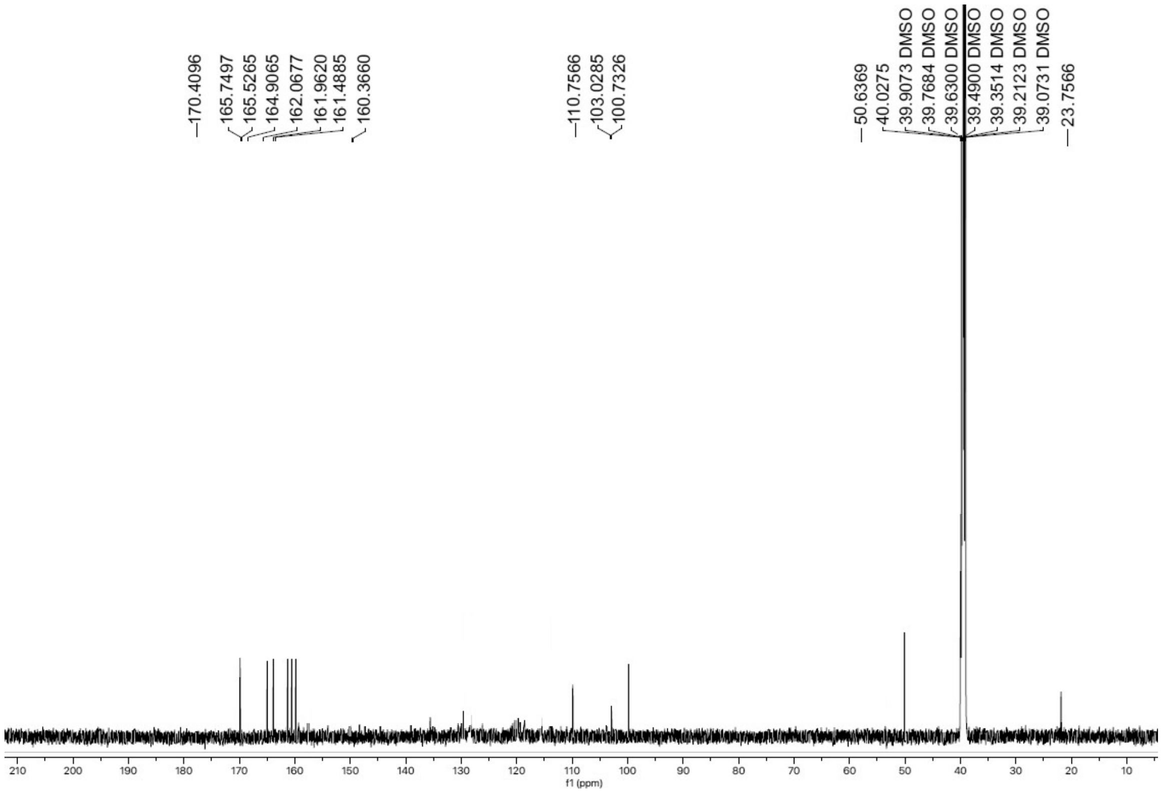

Figure S14. Carbon spectral analysis of compound-3.

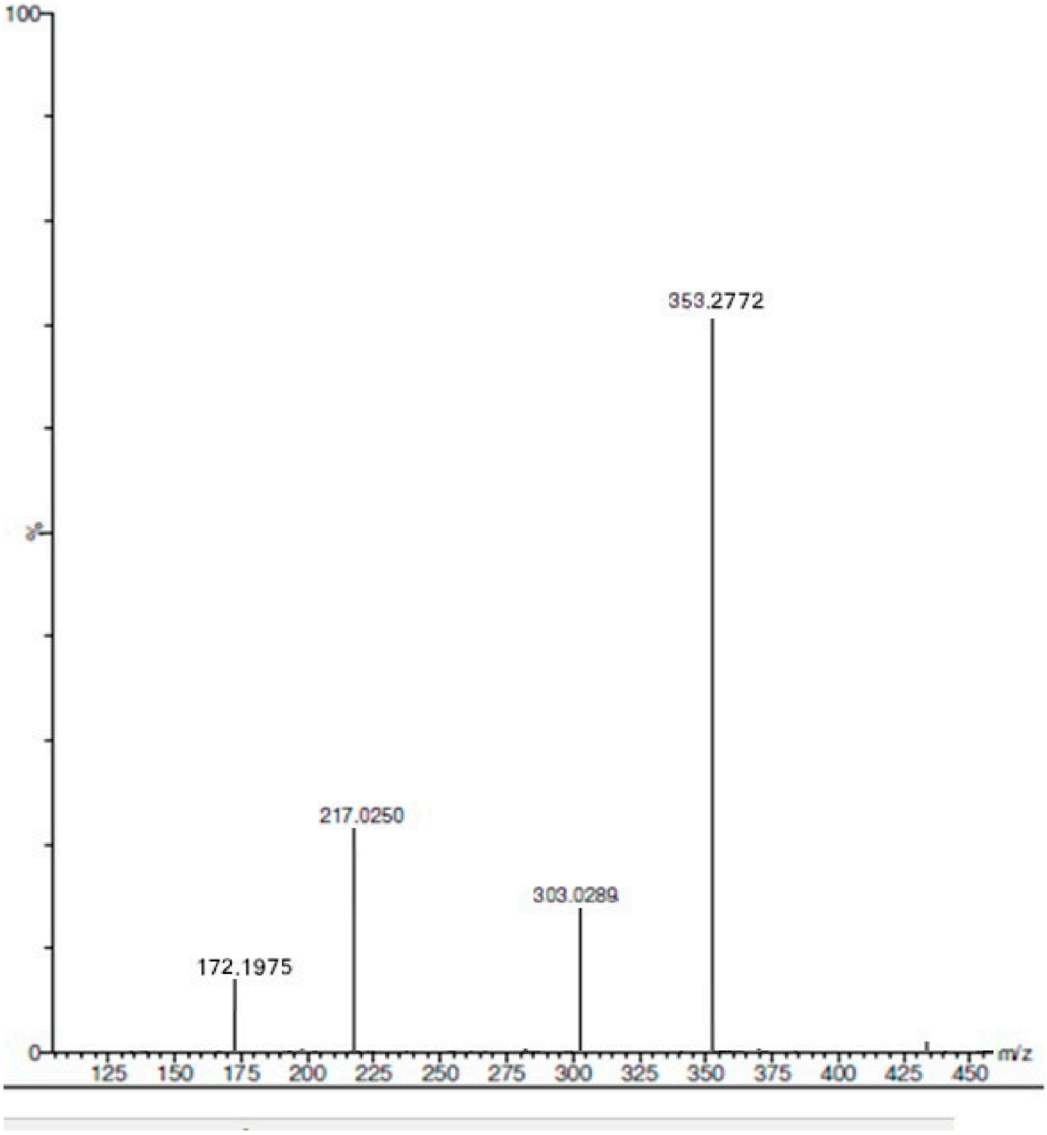

Figure S15. HR-mass spectral analysis of compound-3.

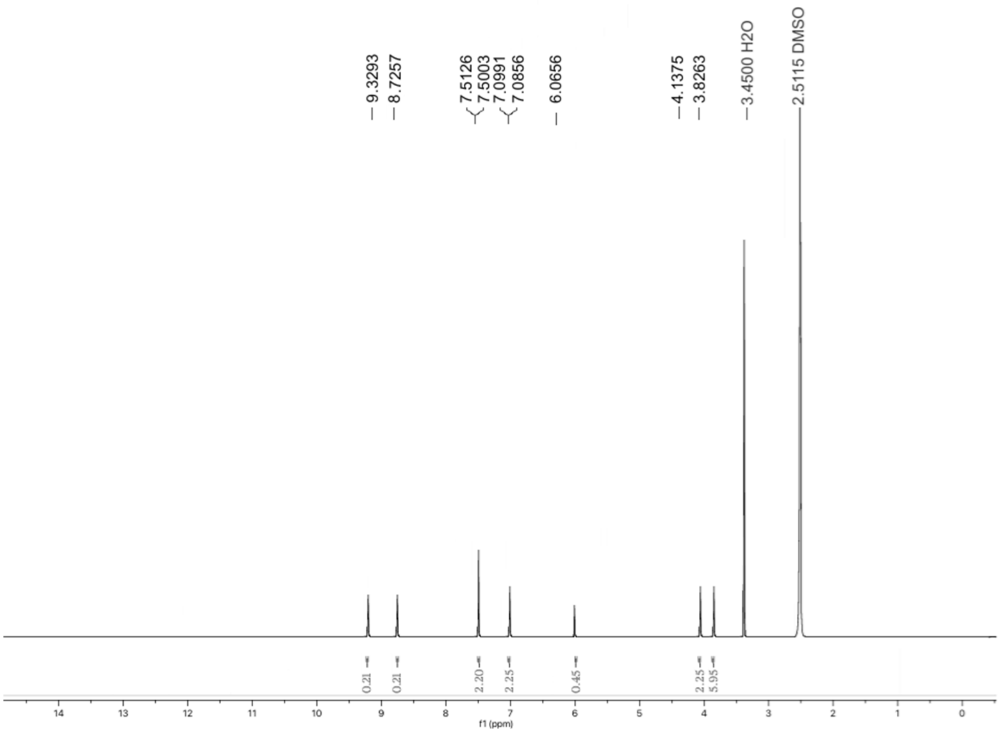

Figure S16. Proton spectral analysis of compound-4.

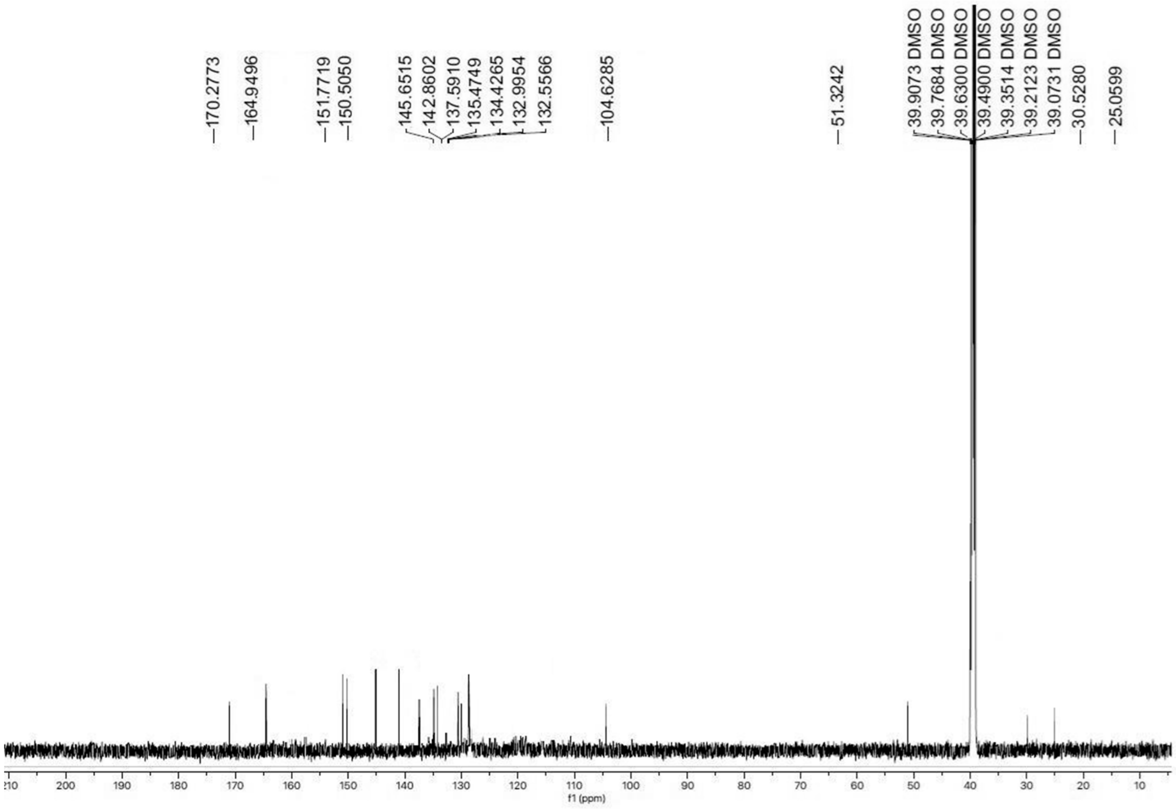

Figure S17. Carbon spectral analysis of compound-4.

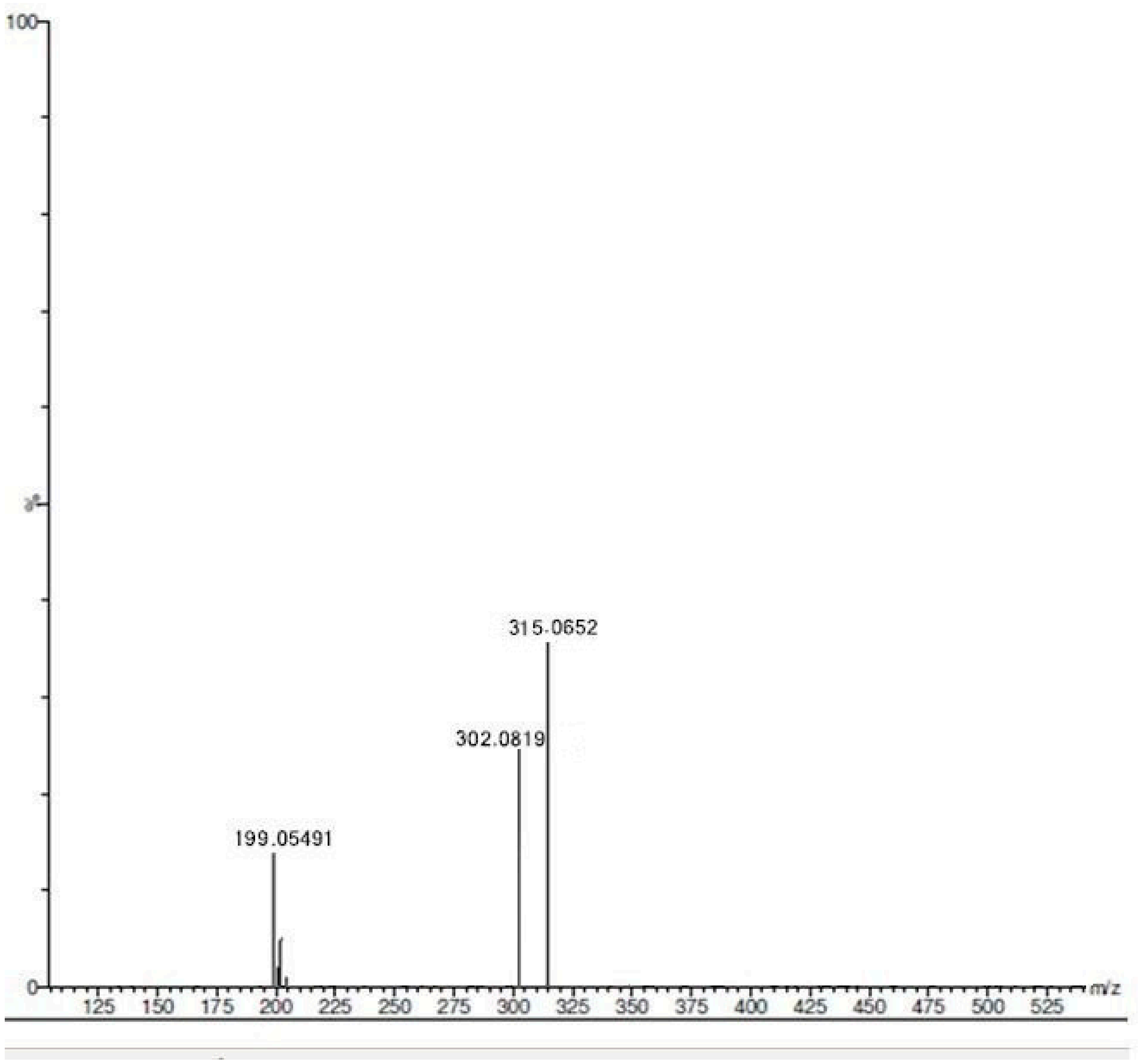

Figure S18. HR-mass spectral analysis of compound-4.

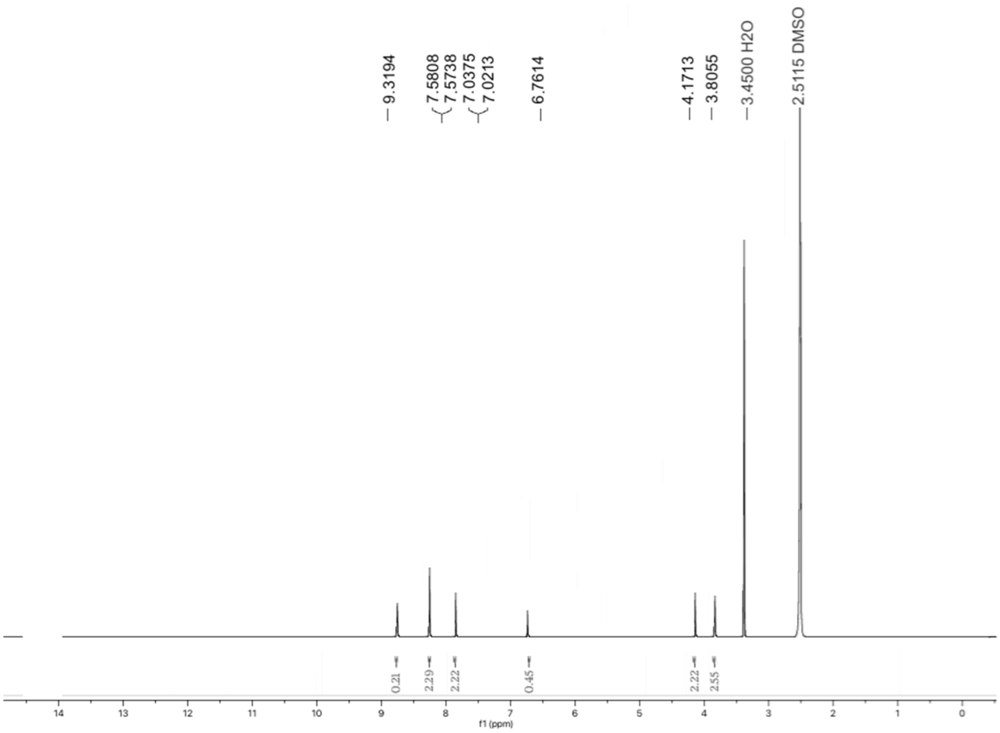

Figure S19. Proton spectral analysis of compound-5.

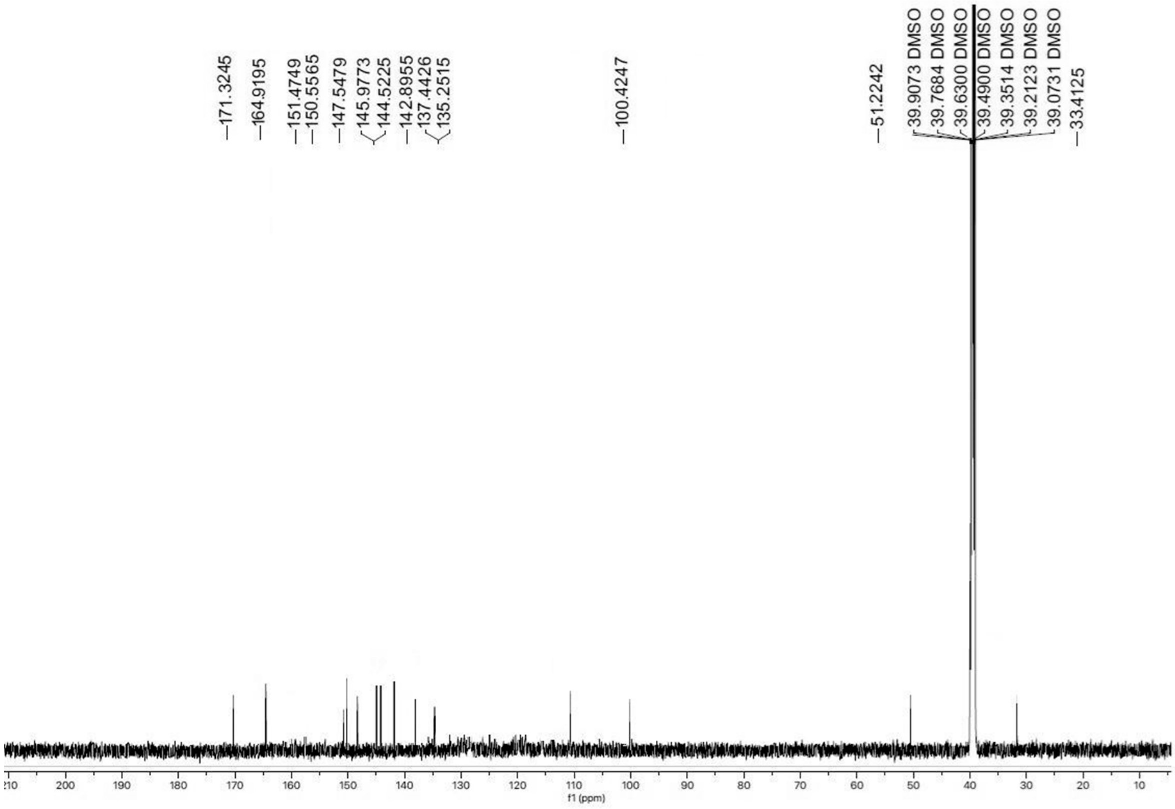

Figure S20. Carbon spectral analysis of compound-5.

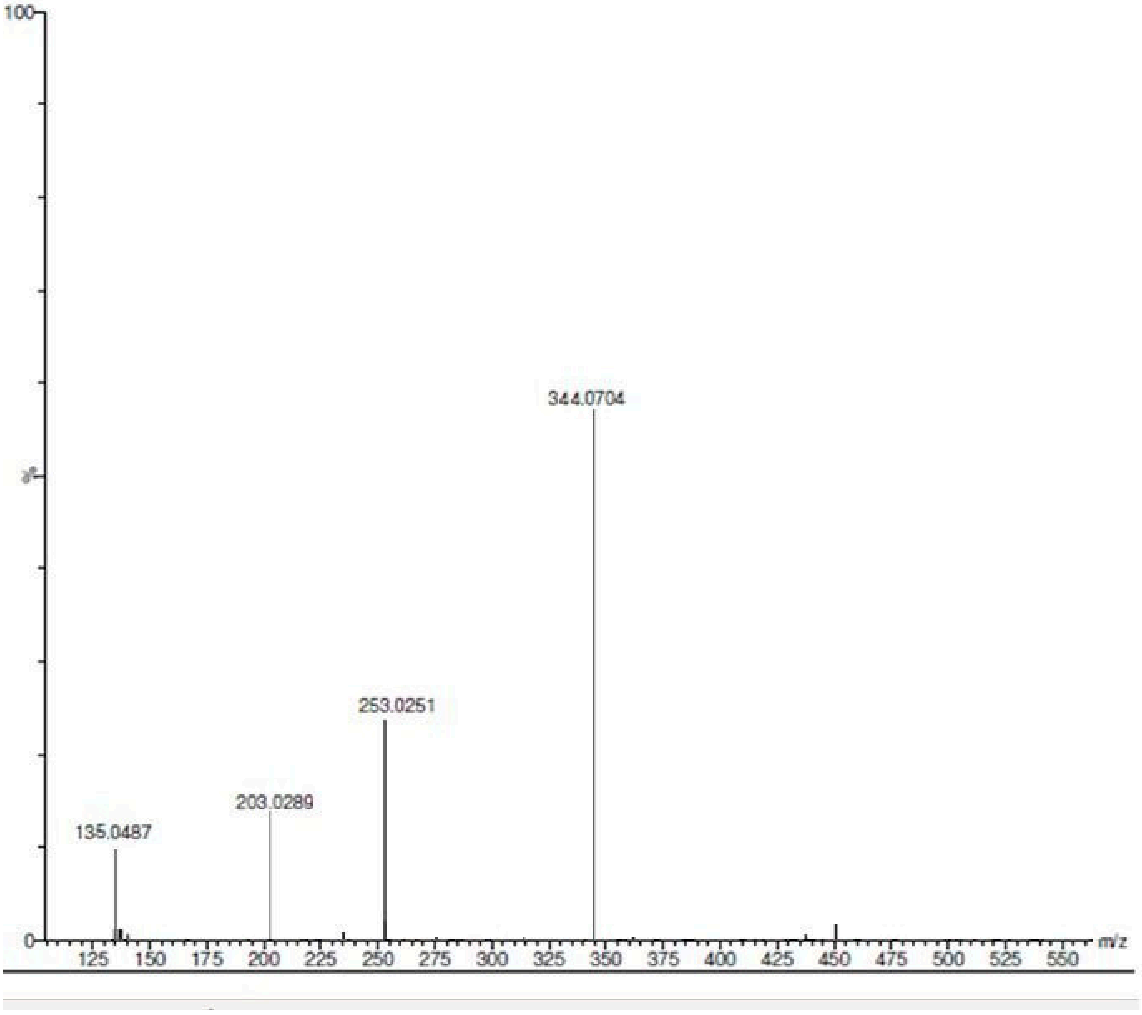

Figure S21. HR-mass spectral analysis of compound-5.

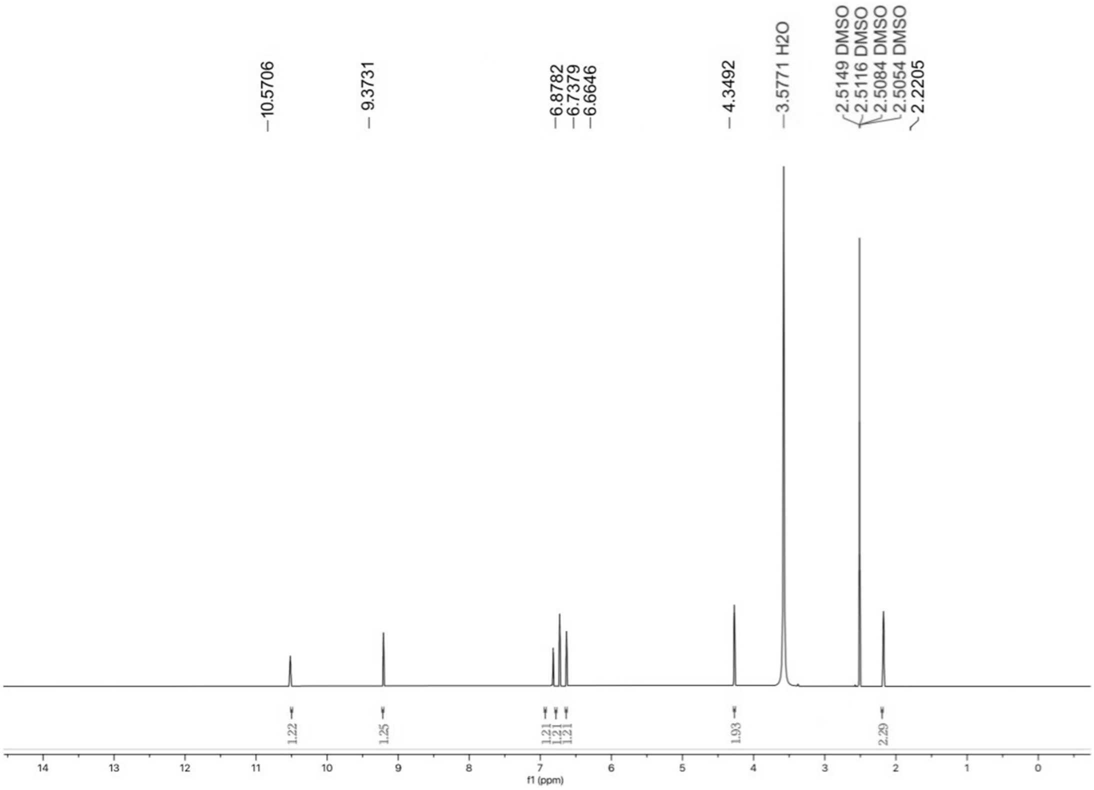

Figure S22. Proton spectral analysis of compound-6.

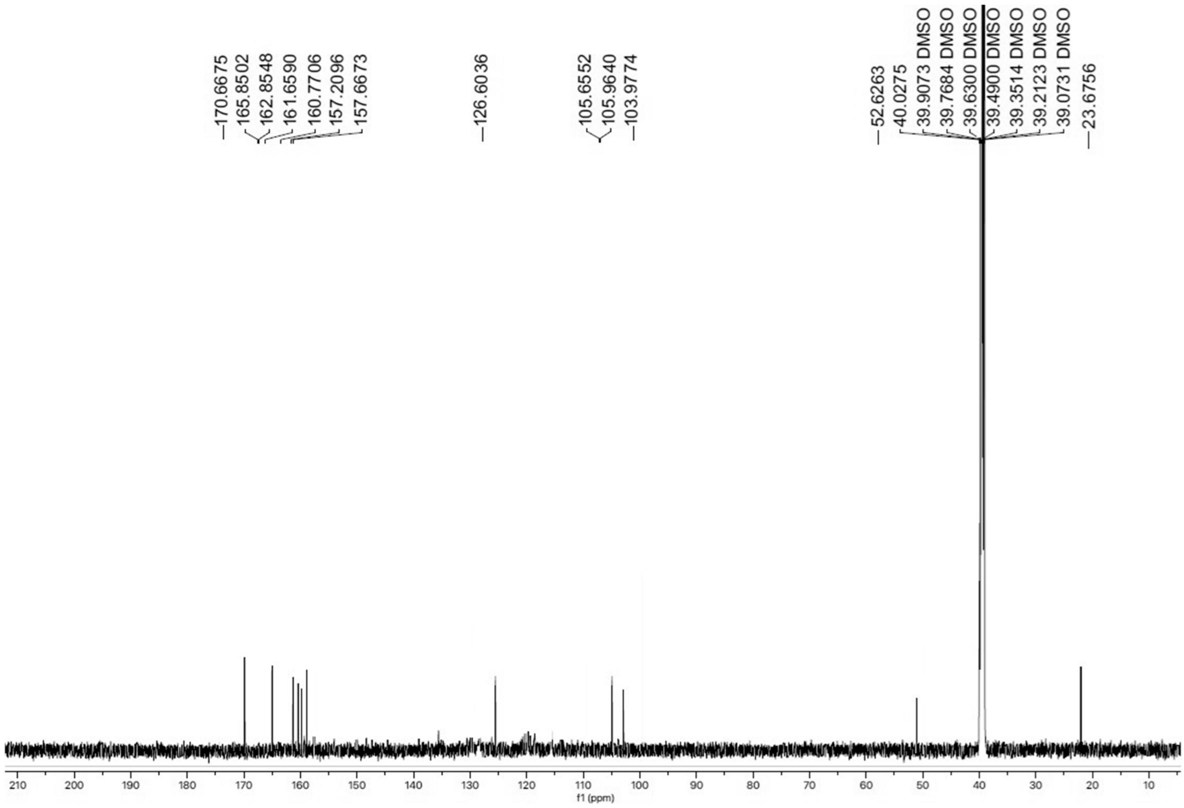

Figure S23. Carbon spectral analysis of compound-6.

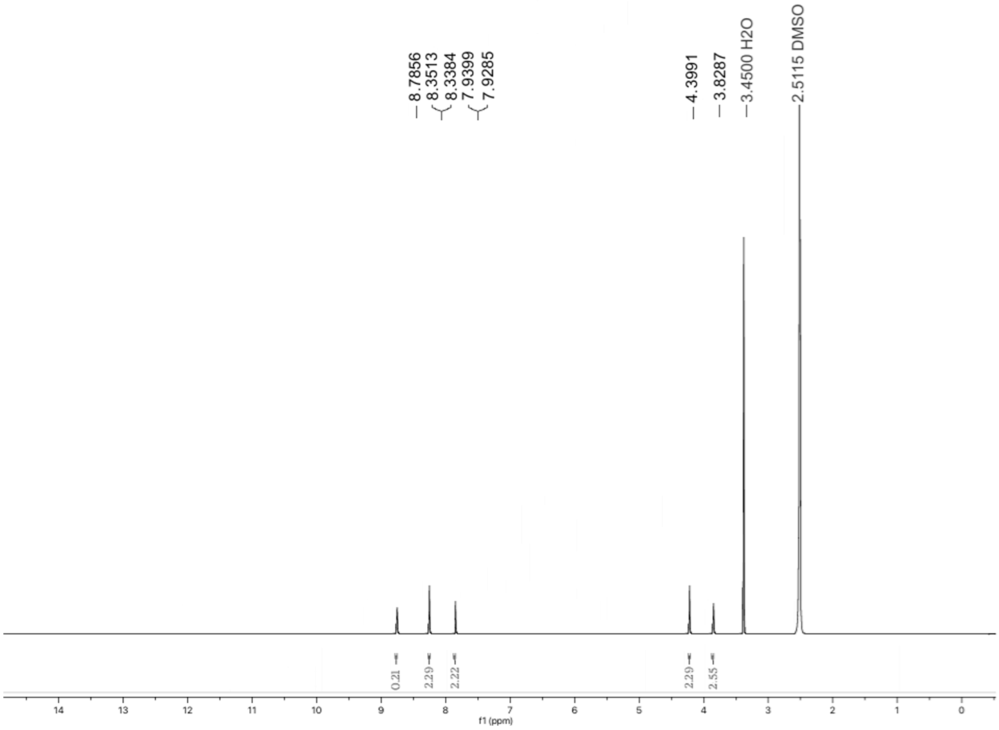

Figure S24. HR-mass spectral analysis of compound-6.

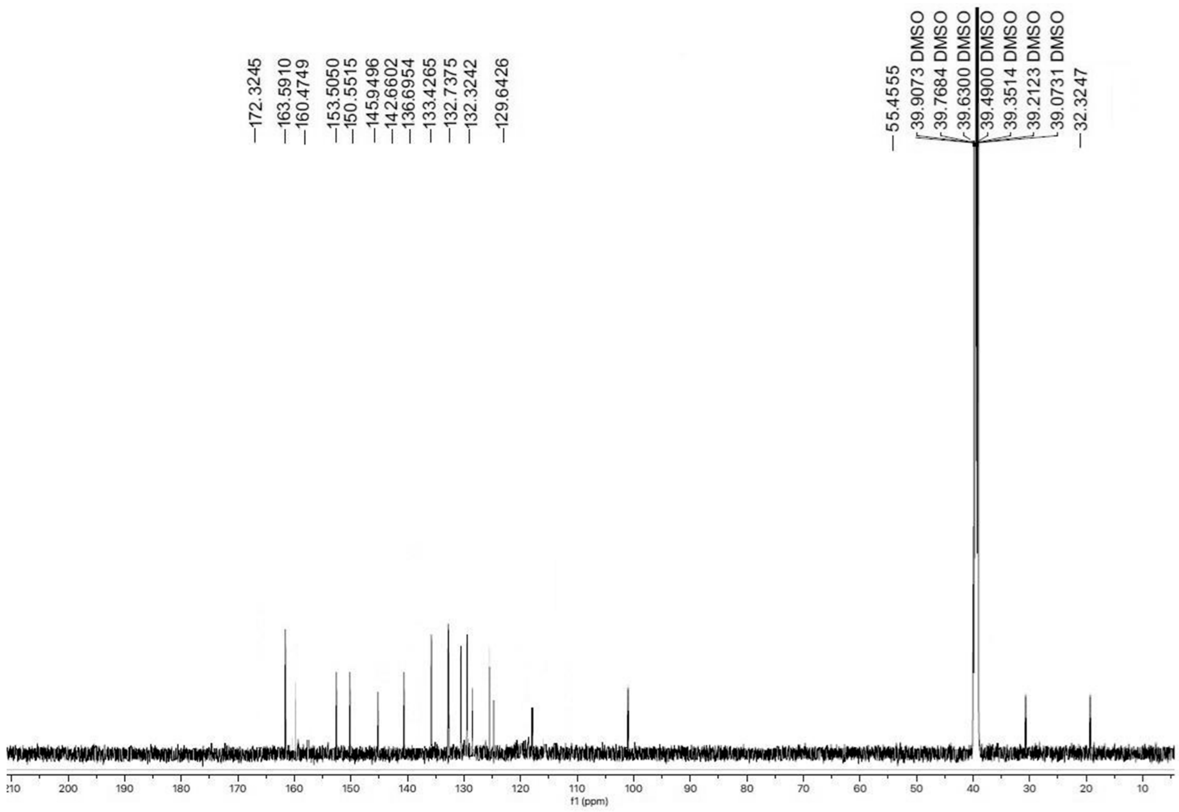

Figure S25. Proton spectral analysis of compound-7.

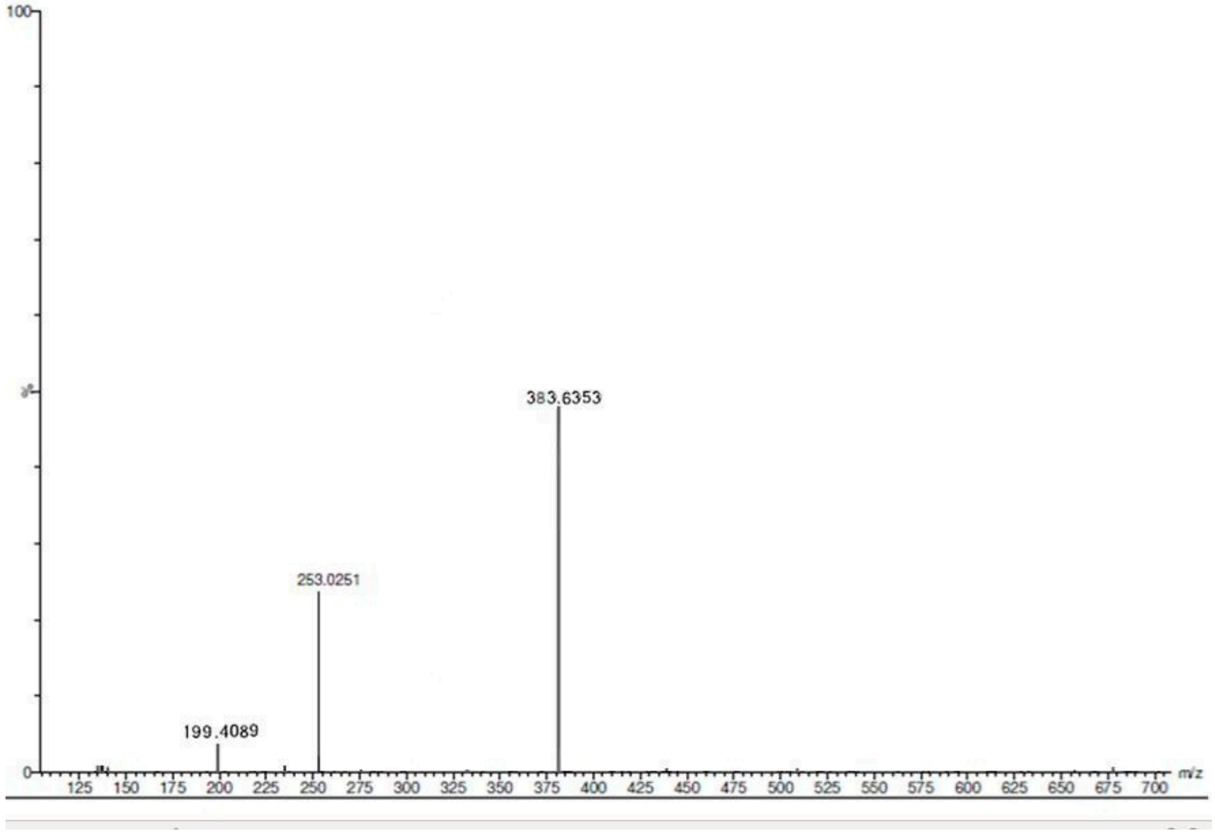

Figure S26. Carbon spectral analysis of compound-7.

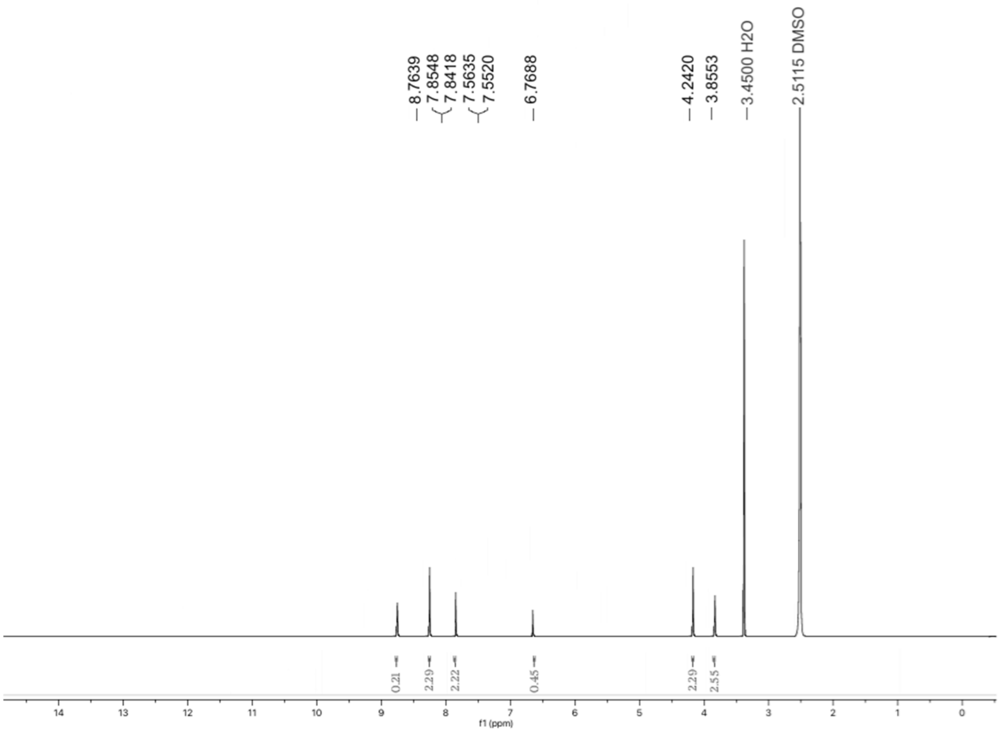

Figure S27. HR-mass spectral analysis of compound-7.

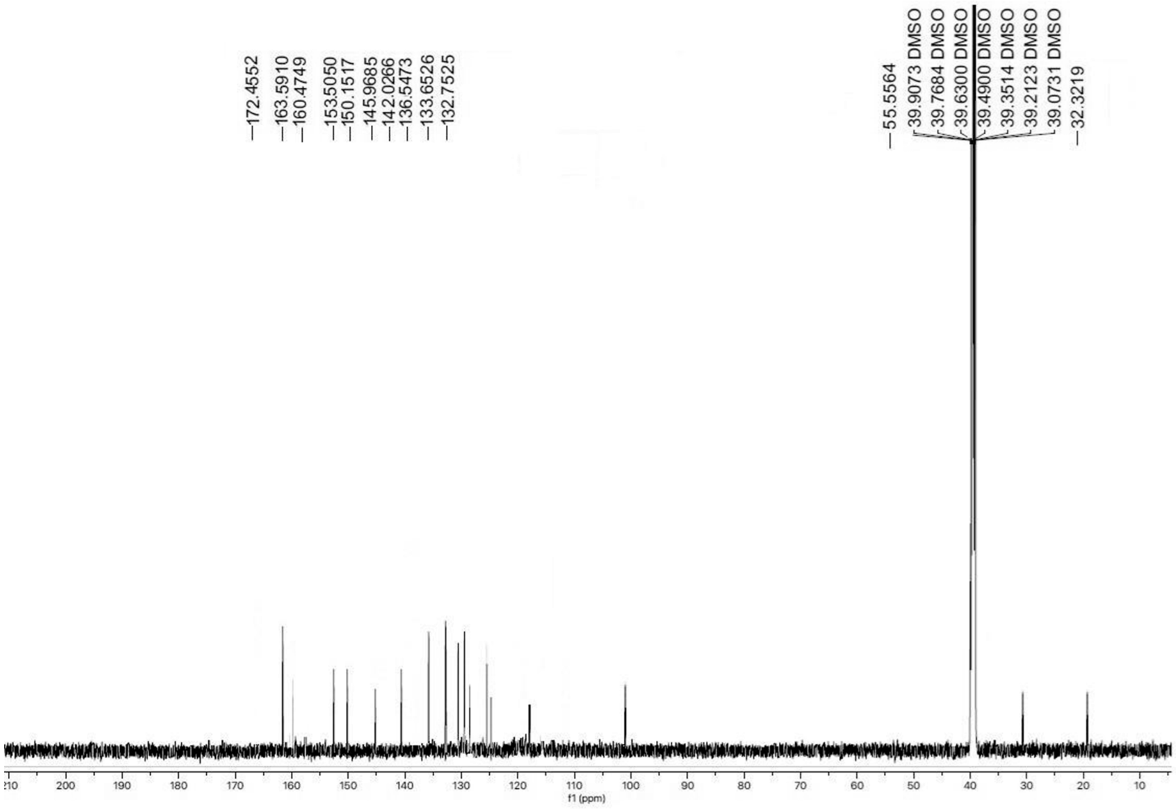

Figure S28. Proton spectral analysis of compound-8.

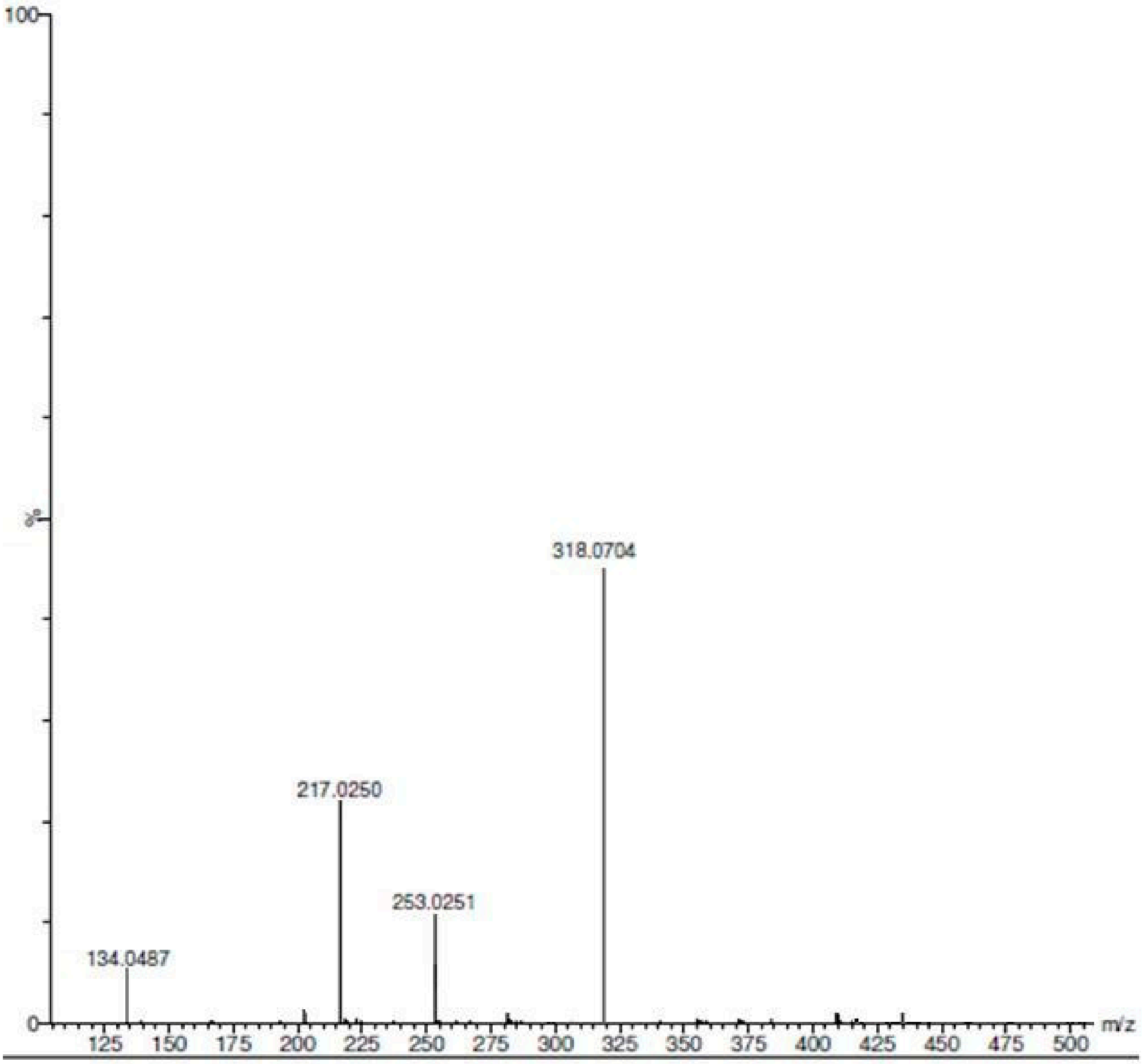

Figure S29. Carbon spectral analysis of compound-8.

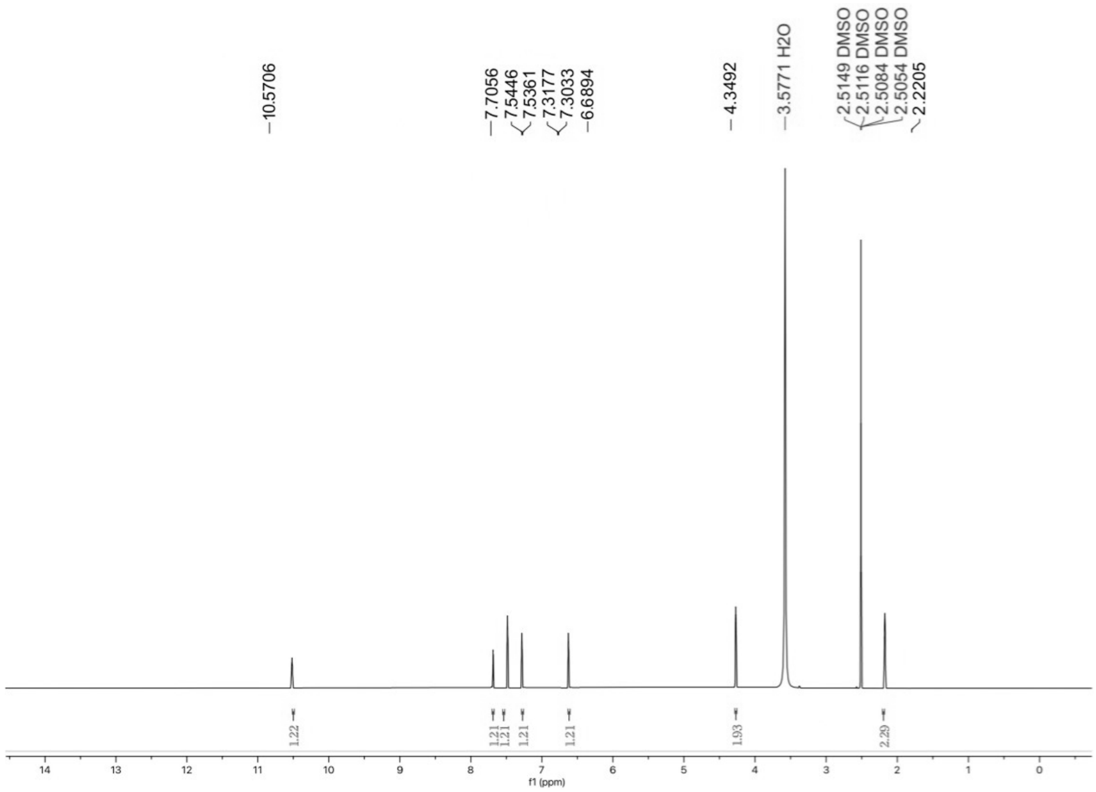

Figure S30. HR-mass spectral analysis of compound-8.

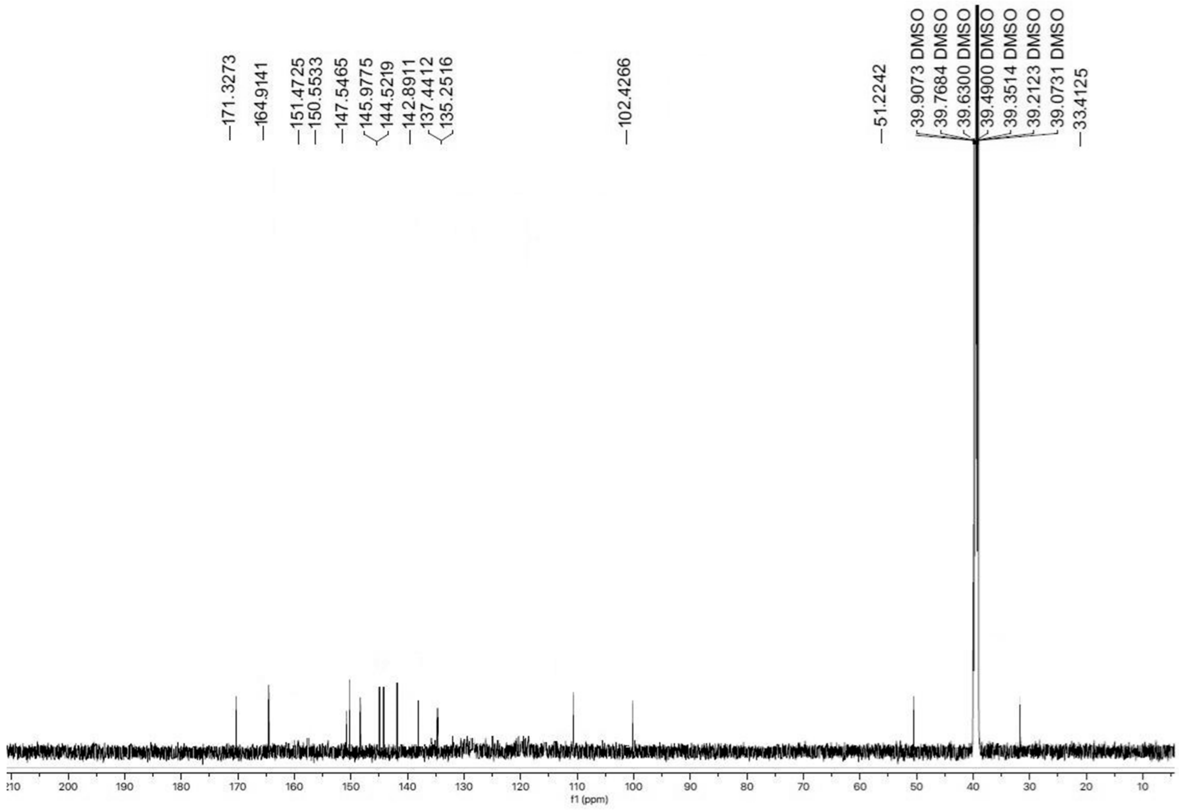

Figure S31. Proton spectral analysis of compound-9.

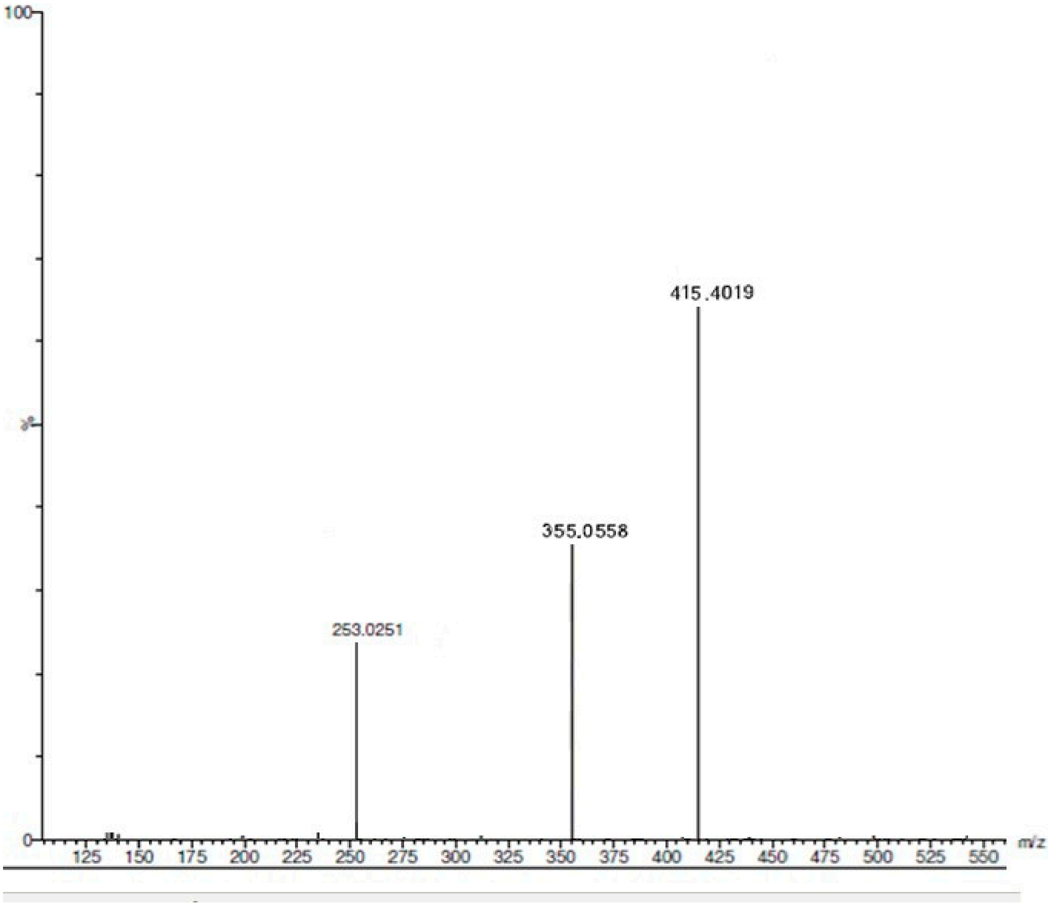

Figure S32. Carbon spectral analysis of compound-9.

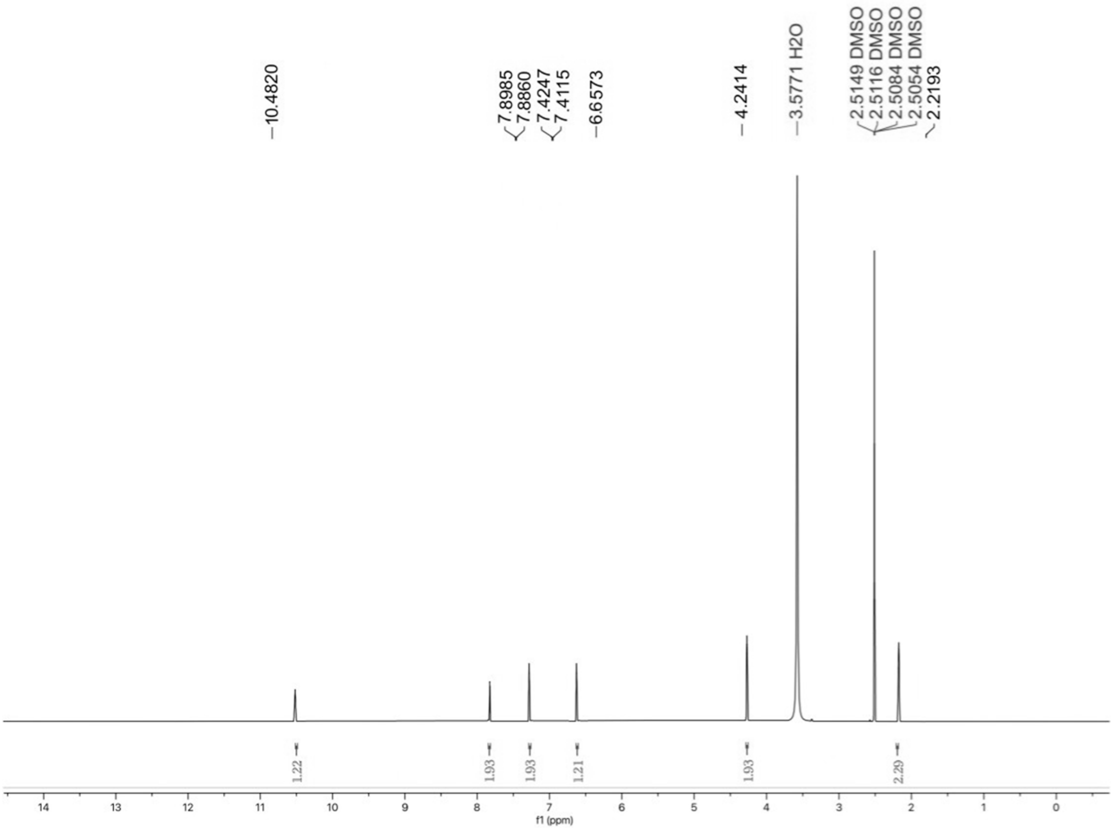

Figure S33. HR-mass spectral analysis of compound-9.

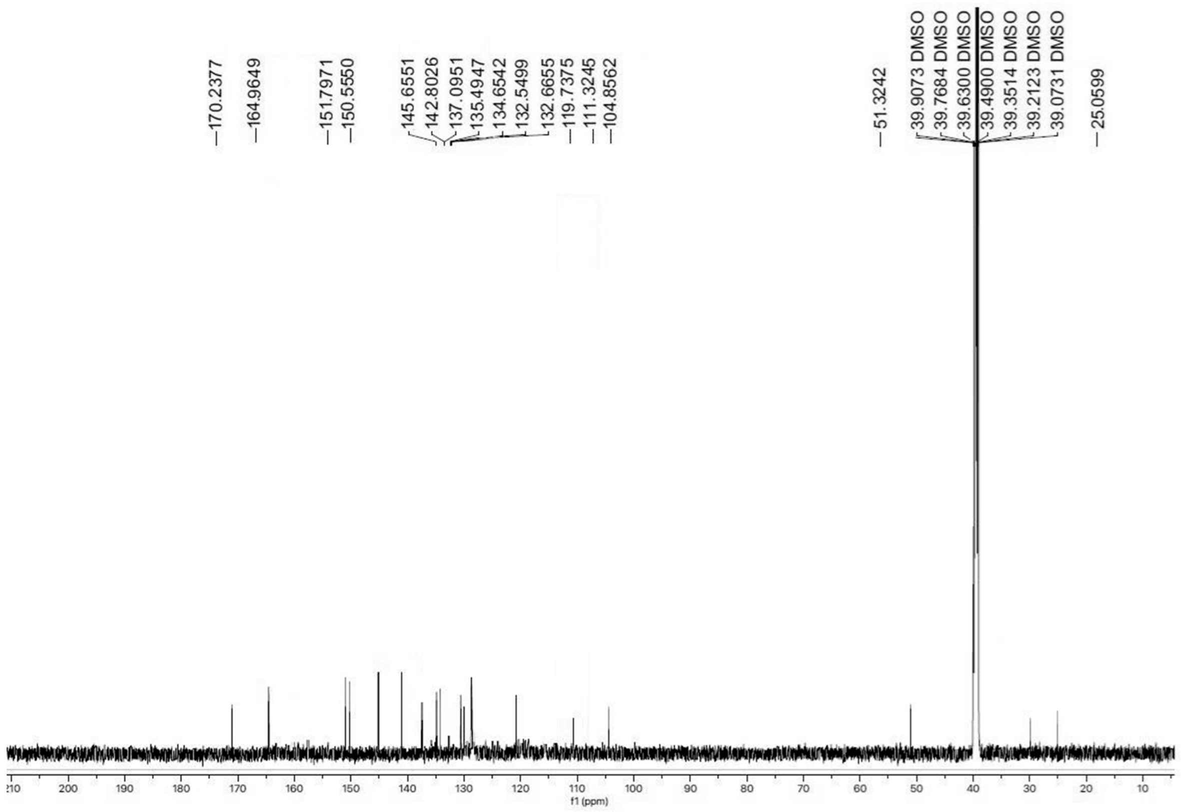

Figure S34. Proton spectral analysis of compound-10.

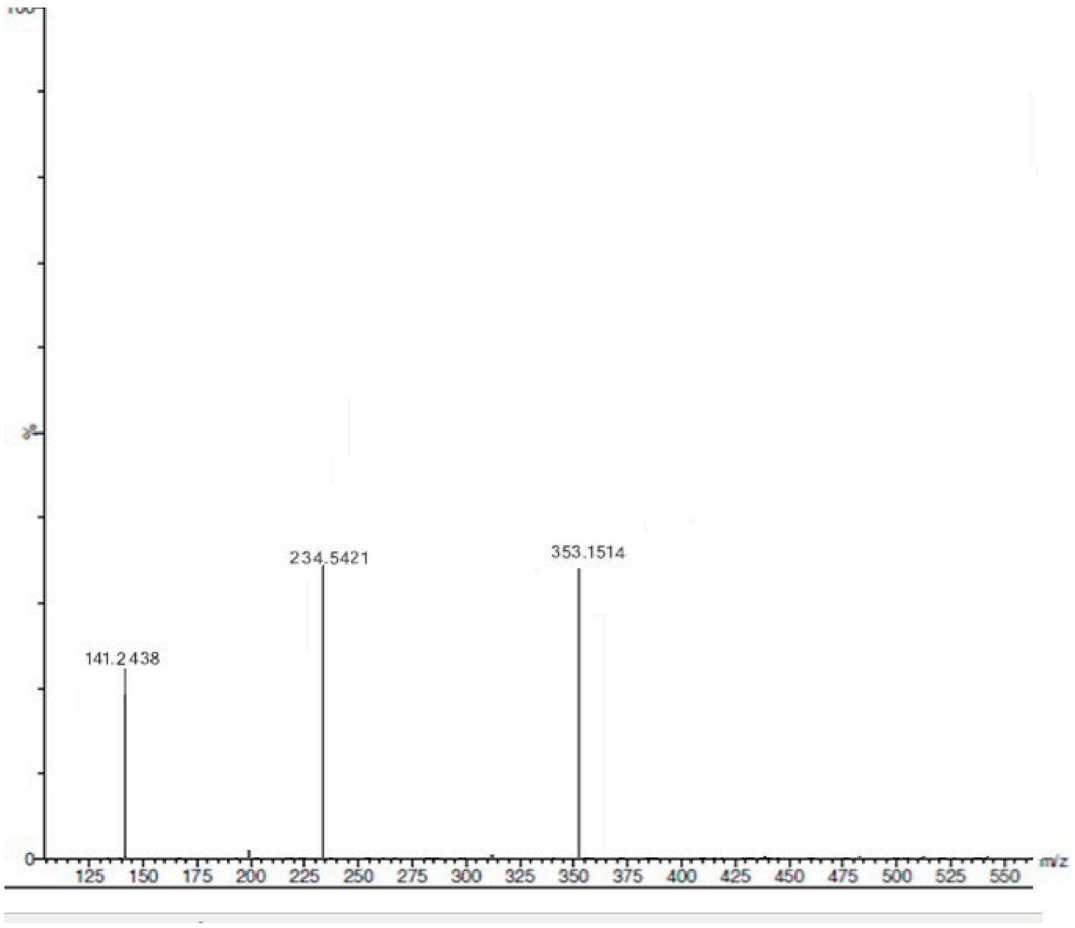

Figure S35. Carbon spectral analysis of compound-10.

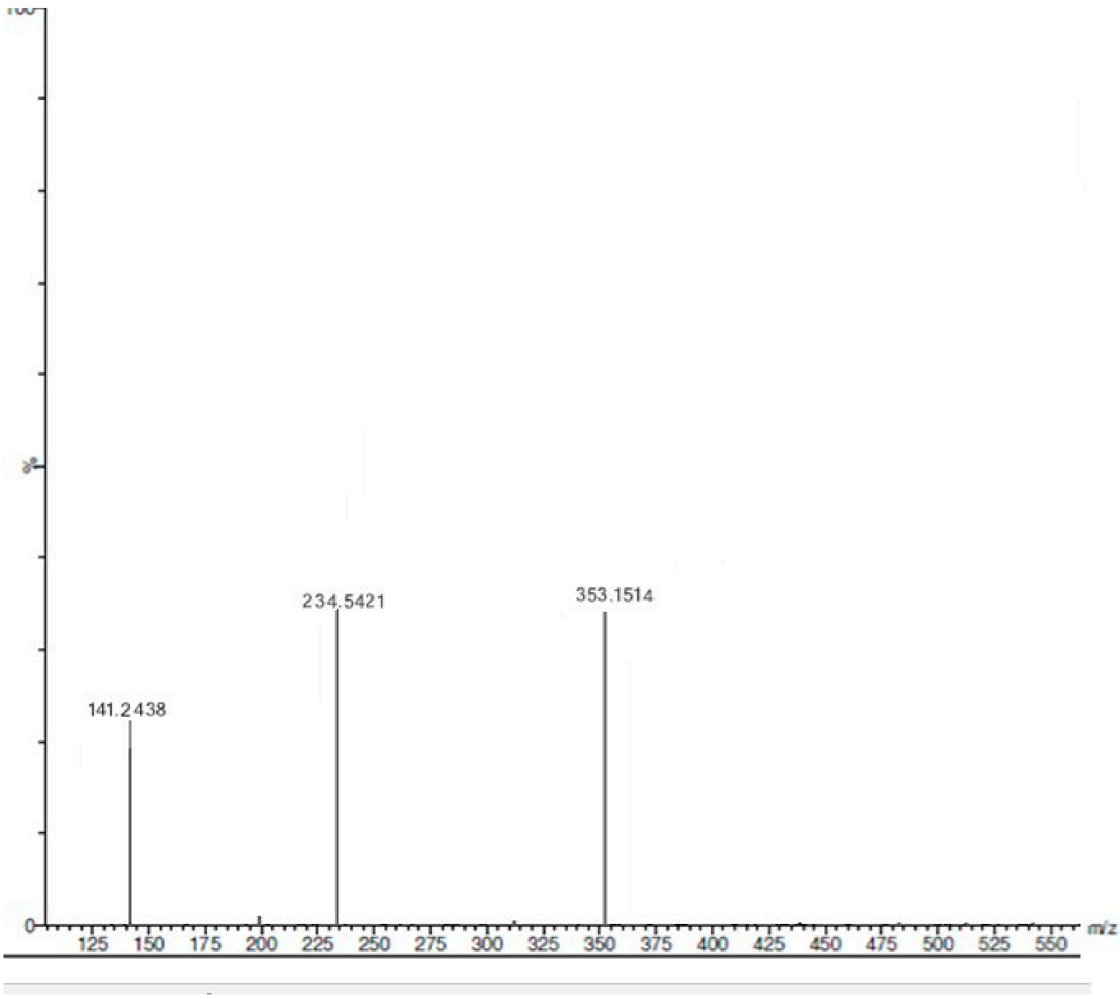

Figure S36. HR-mass spectral analysis of compound-10.

Disclaimer/Publisher's Note: The statements, opinions and data contained in all publications are solely those of the individual author(s) and contributor(s) and not of MDPI and/or the editor(s). MDPI and/or the editor(s) disclaim responsibility for any injury to people or property resulting from any ideas, methods, instructions or products referred to in the content.

https://doi.org/10.3390/ph19071085
